# Compact laboratory-based X-ray microscope enabling nondestructive 3D structure acquisition of mouse nephron with high speed and better user accessibility

**DOI:** 10.1093/jmicro/dfac033

**Published:** 2022-07-02

**Authors:** Naoki Kunishima, Yoshihiro Takeda, Raita Hirose, Satoshi Kume, Mitsuyo Maeda, Akiko Oguchi, Motoko Yanagita, Hirotoshi Shibuya, Masaru Tamura, Yosky Kataoka, Yasuhiro Murakawa, Koichiro Ito, Kazuhiko Omote

**Affiliations:** X-ray Research Laboratory, Rigaku Corporation, Akishima, Tokyo 196-8666, Japan; X-ray Research Laboratory, Rigaku Corporation, Akishima, Tokyo 196-8666, Japan; X-ray Research Laboratory, Rigaku Corporation, Akishima, Tokyo 196-8666, Japan; Laboratory for Pathophysiological and Health Science, RIKEN Center for Biosystems Dynamics Research, Kobe, Hyogo 650-0047, Japan; Center for Health Science Innovation, Osaka City University, Osaka 530-0011, Japan; Multi-Modal Microstructure Analysis Unit, RIKEN-JEOL Collaboration Center, Kobe, Hyogo 650-0047, Japan; RIKEN Center for Integrative Medical Sciences, Yokohama, Kanagawa 230-0045, Japan; Department of Nephrology, Graduate School of Medicine, Kyoto University, Kyoto 606-8501, Japan; Department of Nephrology, Graduate School of Medicine, Kyoto University, Kyoto 606-8501, Japan; Institute for the Advanced Study of Human Biology (ASHBi), Kyoto University, Kyoto 606-8501, Japan; Technology and Development Team for Mouse Phenotype Analysis, RIKEN BioResource Research Center, Tsukuba, Ibaraki 305-0074, Japan; Technology and Development Team for Mouse Phenotype Analysis, RIKEN BioResource Research Center, Tsukuba, Ibaraki 305-0074, Japan; Multi-Modal Microstructure Analysis Unit, RIKEN-JEOL Collaboration Center, Kobe, Hyogo 650-0047, Japan; Laboratory for Cellular Function Imaging, RIKEN Center for Biosystems Dynamics Research, Kobe, Hyogo 650-0047, Japan; RIKEN Center for Integrative Medical Sciences, Yokohama, Kanagawa 230-0045, Japan; Institute for the Advanced Study of Human Biology (ASHBi), Kyoto University, Kyoto 606-8501, Japan; IFOM―the FIRC Institute of Molecular Oncology, Milan 20139, Italy; New Market Development Office, Rigaku Corporation, Akishima, Tokyo 196-8666, Japan; X-ray Research Laboratory, Rigaku Corporation, Akishima, Tokyo 196-8666, Japan

**Keywords:** X-ray microscopy, tomography, 3D rendering, kidney, nephron, biopsy

## Abstract

X-ray microscopes adopting computed tomography enable nondestructive 3D visualization of biological specimens at micron-level resolution without conventional 2D serial sectioning that is a destructive/laborious method and is routinely used for analyzing renal biopsy in clinical diagnosis of kidney diseases. Here we applied a compact commercial system of laboratory-based X-ray microscope to observe a resin-embedded osmium-stained 1-mm strip of a mouse kidney piece as a model of renal biopsy, toward a more efficient diagnosis of kidney diseases. A reconstructed computed tomography image from several hours of data collection using CCD detector allowed us to unambiguously segment a single nephron connected to a renal corpuscle, which was consistent with previous reports using serial sectioning. Histogram analysis on the segmented nephron confirmed that the proximal and distal tubules were distinguishable on the basis of their X-ray opacities. A 3D rendering model of the segmented nephron visualized a convoluted structure of renal tubules neighboring the renal corpuscle and a branched structure of efferent arterioles. Furthermore, another data collection using scientific complementary metal-oxide semiconductor detector with a much shorter data acquisition time of 15 min provided similar results from the same samples. These results suggest a potential application of the compact laboratory-based X-ray microscope to analyze mouse renal biopsy.

## Introduction

As of 2017, the prevalence of chronic kidney disease is estimated at 9.1% of the world’s population [1], indicating the significance of nephrology in medicine. The kidney is a fundamental organ that produces urine from blood. The mammalian kidney plays multiple indispensable roles not only for the excretion of metabolic wastes in blood but also for the control of blood pressure, synthesis of vitamin D, bone mineralization and the promotion of erythrocyte development [2]. The functional unit of the kidney is called the ‘nephron’, which is composed of a renal corpuscle and a renal tubule. The renal corpuscle in which the tubular fluid is produced from blood through an ultrafiltration is composed of a tuft of capillaries called the ‘glomerulus’ and the surrounding origin of the renal tubule called ‘Bowman’s capsule’.

For the clinical diagnosis of human kidney diseases, a histological observation of renal biopsy sample is performed routinely using optical (visible-light) microscopy and electron microscopy [3]. A typical biopsy excised from the kidney looks like an elongated cylinder with a diameter of less than 1.0 mm. The renal biopsy is then aldehyde-fixed and paraffin-embedded for the optical microscopy observation, whereas aldehyde/osmium-fixed and resin-embedded for the electron microscopy observation. Results from both two observation modalities are integrated to make a clinical decision. However, although electron microscopy provides the finest structures of nephron at subcellular resolutions [4], it can visualize only a limited 2D part of the specimen. Although serial block-face scanning electron microscopy [5] can reconstruct a 3D structure based on serial sectioning that integrates a huge number of consecutive sample sections with submicron-scale thicknesses, it may be too laborious to analyze the whole of a renal biopsy. To counter this drawback of electron microscopy, a correlative microscopy with other observation modalities was thought to be helpful. For instance, an optical-microscopy-based 3D reconstruction was developed, in which the 3D structure was reconstructed by serial sectioning from about a thousand consecutive 2.5 μm-thick 2D images of a resin-embedded mouse kidney so that a single nephron could be segmented manually [6,7]. This reconstruction method was successfully applied to confirm the urine concentrating mechanism of rat renal inner medulla [8,9]; efforts toward automated segmentation are in progress [10].

Although the optical-microscopy-based reconstruction contributed significantly to nephron research, this method is still laborious for practical use in clinical diagnosis. It also requires destructive sectioning of samples, resulting in a deterioration of image quality along the direction of sample thickness, which may be misleading to the clinical decision. Furthermore, the sample cannot be reused after optical microscopy observation for electron microscopy, except for special cases including electron microscopy of a paraffin-embedded sample [11] and low-vacuum scanning electron microscopy for a single section [12,13]. Thus, another high-throughput observation modality allowing more versatile sample reuse for electron microscopy would be desirable for medical applications. Nondestructive X-ray microscopy at micron-level of spatial resolution without any serial sectioning would be a promising candidate for such modality [14].

Utilizing higher penetration power of X-ray compared to that of electron beam, X-ray microscopy adopting computed tomography (CT) enables nondestructive, high-throughput and isotropic 3D visualization of bio-specimens at resolutions down to 100 nm [15], although the low contrast of these specimens should be enhanced in many cases by phase retrieval [16–18] or by heavy-atom staining [19–21]. Recently, a speckle-based X-ray phase tomography using synchrotron radiation visualized a whole unstained, hydrated mouse kidney [22], although its spatial resolution was limited to 8 μm. Heavy-atom staining with osmium tetroxide is one useful method to enhance the X-ray absorption contrast in X-ray microscopy. A notable advantage of osmium staining is its compatibility with electron microscopy, which routinely uses osmium tetroxide as a chemically oxidative fixing reagent. For instance, an osmium-stained rat kidney was observed using a synchrotron-based X-ray microscope [23], which traced whole single nephrons although detailed structures in the vicinity of the renal corpuscle were not described. Unfortunately, this experiment was performed using synchrotron radiation with limited accessibility for most researchers. In terms of user’s accessibility, a laboratory-based equipment is highly desired for medical applications. For instance, a laboratory-based X-ray microscope was developed to observe an osmium-stained small invertebrate by a combination with optical microscopy [24], although this system was not commercially available. Toward a more efficient diagnosis of human renal biopsy, here we introduce the application of ‘nano3DX’, a compact, commercially available, laboratory-based X-ray microscope (Rigaku, Tokyo, Japan), to observe an osmium-stained mouse kidney. The kidney piece was embedded in an elongated resin sheet to mimic renal biopsy, thereby allowing us to assess the potential of X-ray microscopy toward the medical application.

## Materials and methods

### Mouse kidney material

All animal experimental protocols were approved by the Ethics Committee on Animal Care and Use of the RIKEN Kobe branch (Permit Number: A2018-03/K2018-EP126), and the procedures were performed in accordance with the Principles of Laboratory Animal Care and with the ARRIVE guidelines (https://arriveguidelines.org). Research staffs were trained in animal care/handling before the experiment. We used two eight-week-old male C57BL/6 J mice for this study. The animals were housed in a cage with a raised mesh base under constant environmental conditions (room temperature, 22–23℃; relative humidity, 50–60%) and a 12-h light–dark cycle (08:00/20:00). Prior to the experiment, food and water were provided *ad libitum*. The animals were monitored for daily weight changes and behaviors for animal health. In addition, in order to avoid a survival endpoint, it was planned to perform the euthanasia when extreme weight loss (more than 15% reduction compared to the previous day) or symptoms for infection such as diarrhea or vomiting were observed in animals. In order to reduce animal suffering, proper animal care management was carried out, paying particular attention to cage cleaning and animal housing density. In the present study, all animals did not meet the euthanasia criterion. In the experiment, the mice were deeply anesthetized using 1.5–3.0% (v/v) isoflurane under monitoring behavior. Immediately after experimentally euthanizing, the mice were perfused transcardially with physiological saline solution, and then switched to a fixing solution of 4% formaldehyde and 2.5% glutaraldehyde buffered with 0.1 M phosphate buffer (pH 7.4) at room temperature; the amount of time elapsed before the euthanasia was within 10 min, and each experiment time until the end of perfusion was within 60 min. The kidney was removed and postfixed overnight in 4% formaldehyde. The kidney specimen was used for the following process. Kidney sections (300 μm or 500 μm thickness) were prepared using a vibratome VT1000S (Leica Microsystems, Inc.), and the sections were manually cut into 1-mm strips to mimic the shape of a biopsy. First, the strips of kidney were immersed in 1% OsO_4_ aqueous solution buffered with 0.1 M phosphate buffer (pH 7.4) at room temperature for 60–120 min. After being washed three times in ultrapure water for 10 min, the pieces were dehydrated through a graded ethanol series (70, 80, 90 and 100%; 15 min each) into propylene oxide (100%, 20 min; two times) at room temperature. Sequentially, the pieces were immersed in 1:1 mixtures of propylene oxide and epoxy resin (Quetol-812 set; Nisshin EM Co., Ltd.) at room temperature overnight. Then, the pieces infiltrated with pure epoxy resin were sandwiched [25–27] between two sheets of Aclar film (Nisshin EM Co., Ltd.) without spacers and weighed down with a fixed bottle (Nisshin EM Co., Ltd.). They were incubated in a pre-warmed oven at 37℃ for 12 h, 50℃ for 12 h and then 64℃ for 48–72 h. Finally, the Aclar film was removed from the embedded kidney pieces, and the samples were used for the X-ray microscope observation in this work.

### Data collection

A resin sheet (about 3 × 10 × 0.3 mm in size) with an osmium-stained mouse kidney piece embedded (about 1 × 5 × 0.3/0.5 mm in size) was fixed with double-sided tape on the flat side of a half-cut tip of a cylindrical metal-stick (3 × 3 × 55 mm in size), and the stick was fixed on a cylindrical metal-jig (12 × 12 × 12 mm in size) using a hexagon socket setscrew. The jig was set on the sample stage of a compact laboratory-based X-ray microscope ‘nano3DX’ (Rigaku, Tokyo, Japan) with a scintillator-based lens and with a 16-bit, 3296 × 2472 pixel (5.5 μm/pixel) CCD detector. A quasi-parallel X-ray beam setting in which the sample-to-detector distance was set much shorter than the source-to-sample distance (260 mm) was adopted to reduce the influence of a light source drift. The sample was scanned with 8.0 keV X-rays from a rotating anode of Cu-target (40 kV, 30 mA; spot size of 70 μm; no filter) to collect 800 projection images with a pixel size of 1.07 μm (L0540 (10×) lens, bin 2) in a step scan mode with an exposure of 20–30 s per frame (4.7–6.9 h in total) and with a sample-to-detector distance of 4 mm. The actual field-of-view used was 1.30 × 1.33 mm since the region of interest was trimmed in the x-direction. To enhance the contrast, the propagation-based phase retrieval [16–18] was unsuccessful; the osmium staining, the limited resolution, and the anisotropic shape of the sample may be relevant. Thus, in this work, a conventional median/Gaussian-based noise filter (denoise; radius of 1 pixel for median, radius of 1 sigma for Gaussian) was adopted, which was more robust when compared to phase retrieval. In addition, we used unfiltered characteristic X-ray from a rotating anode with Cu-target (8.0 keV) to utilize its high intensity. Filtering toward on emission line of characteristic X-ray may be considered in the future. Although we also tried Mo-target (17.5 keV), it was less effective for visualizing the nephron because it tended to emphasize proximal tubules (PTs) more than the Cu-target. Since the specimens were irradiated by 8.0 keV X-rays for 4.7–6.9 h, there may be radiation damage that can influence further observations by different modalities. Additional data collection using scientific complementary metal-oxide semiconductor (sCMOS) detector (16 bit, 2048 × 2048 pixel, 6.5 μm/pixel) were performed for the same specimens in a continuous scan mode under the same conditions except for an exposure time of 0.9 s per frame (15 min in total), field-of-view of 1.30 × 1.30 mm and pixel size of 1.27 μm.

### Image processing and analysis

The CT reconstruction at 16 bits was performed based on a conventional filtered-back-projection method (convolution back projection). The manual segmentation of the nephron from 1236 CT slices (*z* = 0–1325.86 μm) was performed as follows, using the polygon selection tool of *ImageJ* [28]. In each CT slice, the boundary of the nephron was delineated and a mask for the selected nephron area was prepared using the ‘Clear’ tool of *ImageJ*. Then, the mask and the original CT slice were multiplied to produce an isolated area of nephron using the ‘Image calculator’ tool. Consecutive slices with the isolated nephron area were combined to reconstruct a 3D nephron. The boundary was redrawn when positional difference was detected. Of 1236 CT slices in total, 746 CT slices—the 491st to the 1236th (*z* = 525.62–1325.86 μm)—contained the single nephron selected. The 3D rendering was performed using the program *Drishti* [29]. The length of the renal tubule was measured by chain tracing of the tubule using the ‘addpoint/addpath’ tool of *Drishti*. The spatial resolution of a CT image was practically evaluated by an index referred to as ‘size of blurring at edges (SBE)’. SBE was obtained using logistic curve-fitting technique [30] against a line opacity profile across a well-defined edge in the image as described [18]:

(1) $$y = A - \frac{A-B}{1+(\frac{x}{C})^{D}}$$

where variables *x* and *y* represent the position and the value of a pixel, respectively, and the parameters *A* to *D* represent the maximum asymptote value, the minimum asymptote value, the inflection position and the Hill’s slope, respectively; a distance between two positions giving values *A*-0.25(*A-B*) and *A*-0.75(*A-B*) was defined as SBE. This SBE has the same definition as ‘spatial resolution’ in our previous work [18]; the notation was changed to avoid confusion with other conventional indices. Contrast-to-noise ratio (CNR) between two regions of the CT image was calculated as:

(2) $$CNR = {{\left| {{\mu _1} - {\mu _2}} \right|} \over {\sqrt {\sigma _1^2 + \sigma _2^2} }}$$

where *μ*_1_ and *μ*_2_ represent the average opacities of the two regions and *σ*_1_ and *σ*_2_ represent their corresponding standard deviations. This CNR has the same definition as ‘signal-to-noise ratio (SNR)’ in our previous work [18]; we changed the notation considering a terminological preference. For a CNR analysis, a PT region and a distal-tubule (DT) region were selected from a single nephron of which the affiliation was checked carefully by a visual inspection of the CT slices, i.e. tubule tracing. The tracing of the DT assumes a common nephron topology where the tubule in contact with the vascular pole belongs to the same nephron [31]. For each tubule region, an air region in the same CT slice was selected for calculating an air-tubule CNR. A pair of different tubule regions were compared to calculate an intertubule CNR. Thus, a total of 200 measurements were performed in the CNR analysis (Table 1, and Supplementary Figs. S1 and S2): CT images from two different detectors (CCD and sCMOS); five different samples for each CT image; five independent nephrons for each sample; four regions for each nephron (PT, air for PT, DT and air for DT). For SBE/CNR analysis, a 95% confidence interval of the average value was calculated. The statistical significance of the difference between a pair of average values was evaluated by a two-tailed Student’s *t*-test under a null hypothesis of no difference; before the *t*-test, the accordance with a normal distribution and the equality of variances were confirmed at the significance level of 5% using the Kolmogorov–Smirnov test and the *F*-test, respectively.

**Table 1.** Observation statistics

			CNR^d^			
Sample^a^	Transmission^b^	SBE (μm)^c^	Air-PT	Air-DT	*P*-value^e^	PT-DT
1_300_M_t	0.716 0.740	1.59 ± 0.09 1.79 ± 0.11	45.2 ± 2.3 30.3 ± 3.0	32.1 ± 2.0 22.4 ± 1.1	3.4 × 10^−5^ 1.4 × 10^−3^	12.7 ± 0.9 7.9 ± 1.9
2_500_M_t	0.658 0.665	1.68 ± 0.14 1.79 ± 0.10	38.0 ± 1.0 24.5 ± 1.7	30.9 ± 1.3 18.5 ± 1.3	3.0 × 10^−5^ 6.8 × 10^−4^	9.1 ± 1.8 5.7 ± 1.6
1_300_H_t	0.699 0.713	1.63 ± 0.08 1.80 ± 0.09	45.7 ± 2.1 32.9 ± 2.7	27.9 ± 1.5 22.9 ± 1.4	8.6 × 10^−7^ 1.9 × 10^−4^	15.2 ± 1.3 9.1 ± 1.6
2_300_H_b	0.774 0.798	1.67 ± 0.11 1.75 ± 0.13	46.9 ± 1.6 27.2 ± 2.1	29.1 ± 2.7 18.6 ± 1.1	3.8 × 10^−6^ 1.1 × 10^−4^	17.4 ± 2.6 8.6 ± 2.1
2_500_H_b	0.660 0.681	1.52 ± 0.12 1.70 ± 0.09	40.0 ± 1.3 26.4 ± 1.5	32.0 ± 1.6 20.5 ± 1.1	6.6 × 10^−5^ 2.6 × 10^−4^	9.9 ± 0.8 3.8 ± 1.7

A value with range denotes an average with 95% confidence interval from five independent measurements. Top and bottom lines are results from data taken by CCD and sCMOS detectors, respectively.

a Abbreviations used are: the top region of 300-micron piece from kidney sample No. 1 embedded in medium resin for 1_300_M_t, the top region of 500-micron piece from kidney sample No. 2 embedded in medium resin for 2_500_M_t, the top region of 300-micron piece from kidney sample No. 1 embedded in hard resin for 1_300_H_t, the bottom region of 300-micron piece from kidney sample No. 2 embedded in hard resin for 2_300_H_b and the bottom region of 500-micron piece from kidney sample No. 2 embedded in hard resin for 2_500_H_b. Exposure time per frame for CCD data was: 20 s for 300-μm samples (4.7 h in total), 25 s for 2_500_M_t (5.8 h in total) and 30 s for 2_500_H_b (6.9 h in total).

b Calculated value from the opacity histogram of a projection image at 0° of each data set; the peak opacity of the proximal tubule was divided by the peak opacity of air.

c Size of blurring at edges of resin-sample boundary estimated based on the Eq.1 from a 20 μm line profile.

d Calculated value from an opacity comparison between two selected regions (5 × 5 pixel) of CT image (see Supplementary Figs. S1 and S2 in detail). PT and DT denote proximal and distal tubules, respectively. Air-PT and air-DT denote air-tubule CNRs and PT-DT denotes an intertubule CNR; a pair of indicated CT regions were compared based on the Eq. 2.

e Average CNR values of air-PT and air-DT were compared by Student’s *t*-test for each result, according to the procedure described in Materials and methods.

## Results

### Observation of mouse kidney

**Fig. 1.**
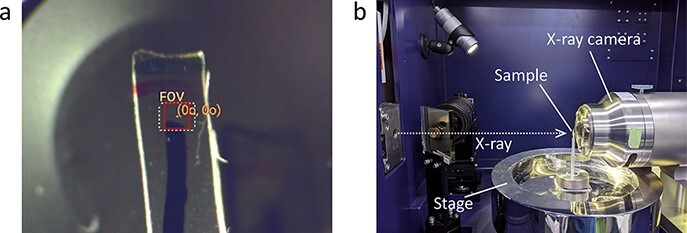
Observation of mouse kidney using laboratory-based X-ray microscopy. (a) Optical monitor image of kidney sample mounted for experiment. The solid-line square (1.30 mm × 1.33 mm) represents the field-of-view for the X-ray scanning; note that the actual scanning area is shifted by about a millimeter due to a misalignment of the optical monitor. (b) Configuration of X-ray scanning. Representative parts of the apparatus are labeled with names; note that the sample shown is a plastic piece for calibration.

**Fig. 2.**
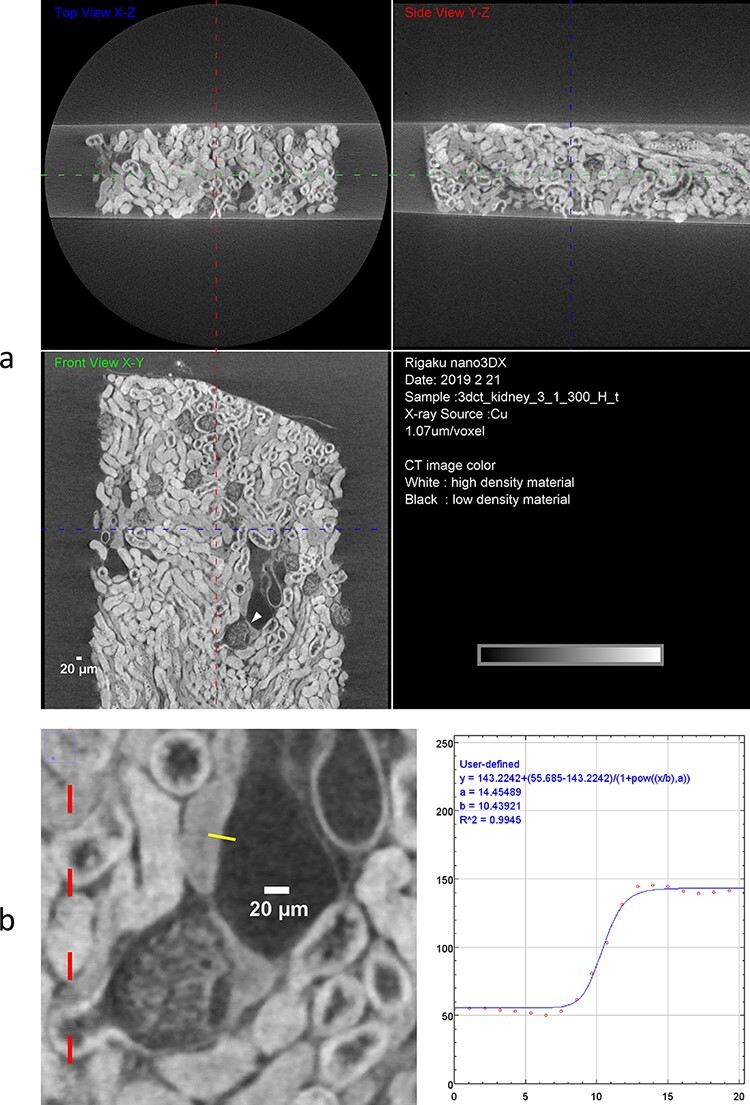
CT reconstruction. (a) Orthogonal CT slices from three different perspectives. These images are derived from the sample shown in Fig. 1a and in Table 1 (1_300_H_t). A renal corpuscle used for the nephron segmentation is indicated by an arrowhead. Conditions for the data collection are described in Materials and methods. (b) Measurement of SBE. Magnified part of (a) around the arrowhead is presented with an opacity profile along the solid line orthogonal to resin-tissue boundary (about 20 μm). Obtained SBE from the curve fitting was 1.59 μm.

**Fig. 3.**
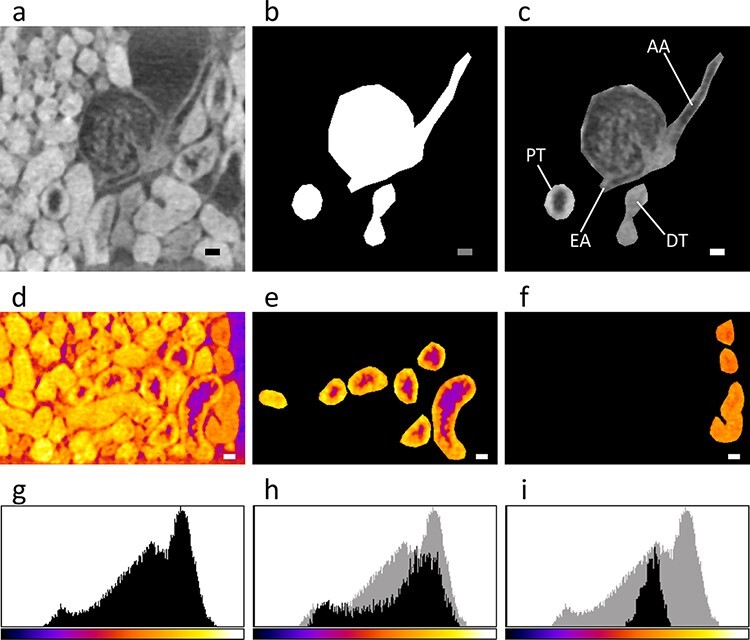
Segmentation of single nephron. The CT data used was from the observation of mouse kidney sample shown in Fig. 1a and in Table 1 (1_300_H_t). This figure was produced using the program *ImageJ* [28]. Scale bars: 20 μm. The manual segmentation procedure is explained (a–c). (a) Original 968th CT section (*z* = 1037.30–1038.37 μm). It is magnified around a renal corpuscle corresponding to that indicated by an arrow in Fig. 2. (b) Corresponding mask. White regions specify the nephron to be segmented out. (c) Corresponding nephron segmented. It was produced by multiplying the CT slice and the mask. Parts of the nephron are labeled; AA, EA, PT and DT represent the afferent arteriole, the efferent arteriole, the proximal tubule and the distal tubule, respectively. The segmented nephron regions were analyzed on the 871st CT section (d–i; *z* = 933.25–934.32 μm). The X-ray opacity in the range of 0–255 was color-coded using the lookup table ‘Fire’ of *ImageJ*. For a part of the CT section that was trimmed so as to fit the single nephron, selected areas (d–f) and corresponding opacity histograms (g–i) are shown. (d, g) Original CT section. Proximal (e, h) and distal (f, i) tubules for the segmented single nephron. The histograms for the tubules are overlaid with that for the original section as a gray background.

Using a compact laboratory-based X-ray microscope, we observed a 1-mm strip of a mouse kidney piece mimicking the shape of a human biopsy. To enhance the low contrast of the kidney tissue, the sample was stained with osmium tetroxide, dehydrated with a series of alcohol baths of increasing concentrations and then embedded into a resin sheet, as described in Materials and methods (Fig. 1a). The resin sheet was placed on the sample stage of the microscope and irradiated by Cu-target X-rays for 4.7–6.9 h to collect projection images using a CCD detector (Fig. 1b). Five independent specimens from combinations of two mice, two different types of resin (hard/medium) and two different thicknesses of kidney sample (300/500 μm) were examined. All the five specimens produced similar results regarding the SBE (an index to evaluate spatial resolution) and the CNR, although the thinner samples provided slightly higher X-ray transmission values (Table 1 and Supplementary Fig. S1). Reconstructed CT image showed multiple renal glomeruli and diverse tubular structures in any specimen (Fig. 2, Supplementary Fig. S1 and Supplementary Videos 3 and 5). SBE attained by CCD detector was 1.5–1.7 μm in all directions of any specimen (Table 1), which was enough for resolving detailed internal structures of the nephron, such as glomerular capillary (Fig. 3c). The resin embedding was effective in reducing sample motion during data collection. The thin strip shape of the kidney piece was important to search for a continuous nephron as well as to keep acceptable levels of X-ray transmission. To evaluate the influence of used detector type, the same specimens were scanned by sCMOS detector. As a result, sCMOS provided similar SBEs and lower CNRs from much shorter data acquisition time of 15 min, comparing with CCD (Table 1 and Supplementary Figs. S1 and S2). Thus, sCMOS detector may be selected for a quick observation for diagnostic purposes, although CCD was adopted in this work for image analysis and 3D rendering.

### Segmentation of nephron

Using the CT slices obtained, area segmentation of a single nephron was performed (Fig. 3a–c and Supplementary Videos 3 and 4). First, vessels and tubules belonging to a single nephron were traced on each CT section. The tracing of the DT assumes a common nephron topology where the tubule in contact with the vascular pole belongs to the same nephron [31]. A continuous nephron—except for a gap in the PT—was found in the top region of a 300-μm-thick piece from kidney sample No. 1 embedded in a hard resin. Then, a single nephron was isolated and reconstructed using a mask that was prepared from a delineation of the nephron boundary. In the segmented nephron, the DT looks darker on the CT slices compared to the PT, probably due to a difference in reabsorption capacities with osmium tetroxide between PT and DT (Fig. 3c and Supplementary Videos 3 and 4). This phenomenon may suggest that the two types of tubules can be distinguished from their X-ray opacities. This possibility was also pointed out by a previous report using synchrotron X-rays on an osmium-stained rat kidney, although there was no supporting data [23]. Therefore, to examine this possibility, we have analyzed the X-ray opacity histograms of segmented tubules. As a result, the two types of tubules produced distinct opacity peaks (Fig. 3d–i), confirming that PT and DT were distinguishable by opacity. The generality of this result was further confirmed by a CNR analysis between PT and DT from five independent nephrons in each kidney sample (Table 1). When the opacities in air and tubule regions were compared in any sample, the difference between average values of air-PT CNRs and air-DT CNRs was statistically significant. This difference corresponds to a high average value of intertubule (PT-DT) CNRs, indicating that discrimination is possible even in the thicker samples. It is worth noting that the X-ray opacities for vessels are similar to those for the DT. The osmium staining selectively visualizes osmiophilic materials such as cellular constitutive lipids with unsaturated bonds [32]. Taken together, the observed differences in X-ray opacities for various nephron parts may reflect their content of osmiophilic materials.

### Spatial relationship between nephron parts

**Fig. 4.**
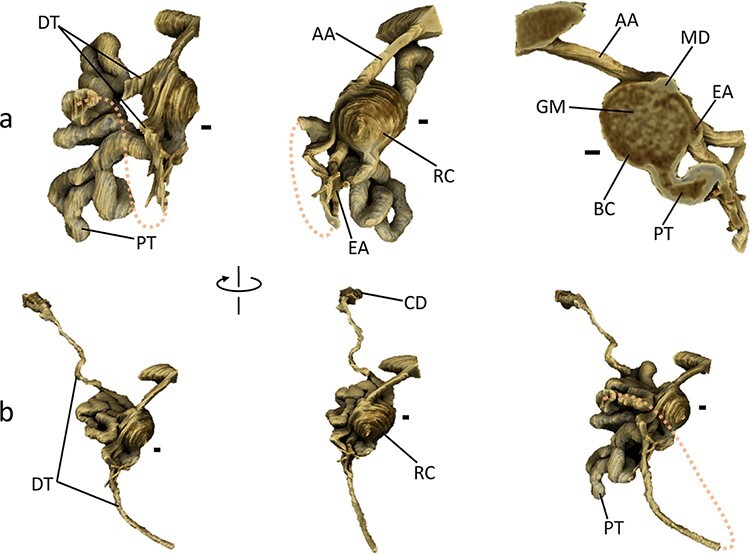
3D rendering of single mouse nephron. The 3D rendering models were produced using the program *Drishti* [29]. The nephron segmented harbors a renal corpuscle corresponding to that indicated by an arrow in Fig. 2. A shortcut in the proximal tubule is indicated by dotted lines. Perspectives of the left and the center models rotate about 60° to each other along the vertical axis. Parts of the nephron are labeled: RC, GM, BC, MD, AA, EA, PT, DT, and CD representing the renal corpuscle, the glomerulus, Bowman’s capsule, the macula densa, the afferent arteriole, the efferent arteriole, the proximal tubule, the distal tubule and the collecting duct, respectively. Scale bars: 20 μm. Parallel lines on the model surface are artifacts due to a technical limitation of the manual segmentation. (a) Nephron models in the vicinity of the renal corpuscle from three perspectives. The right model shows a zoomed-up cross-section of the renal corpuscle viewed from the back side of the center model. (b) Nephron models from the renal corpuscle to the collecting duct. The right model shows a complete nephron except for the proximal shortcut; the perspective is similar to that of the left model.

The renal tubule transports the tubular fluid from Bowman’s capsule to the collecting duct through the PT, the loop of Henle, the DT and the connecting tubule, which runs more than 10 mm in the case of a mouse [31]. As an application of laboratory-based X-ray microscopy, a 3D rendering of a single nephron was performed (Fig. 4 and Supplementary Video 6). This model contained a 2.1-mm section of the PT and a 1.9-mm section of the DT, indicating that a maximum 40% of the tubule entire was traced. The 3D rendering model visualized characteristic nephron parts. The renal corpuscle comprising the glomerulus and the Bowman’s capsule is roughly spherical in shape and has a diameter of about 120 μm (Fig. 4a), which is in agreement with the largest mouse renal corpuscle reported [6] from the optical-microscopy-based reconstruction. Certain glomerular capillaries are visible inside the glomerulus, although the better resolution/contrast may be required to trace them. The vascular pole of the renal corpuscle has afferent and efferent arterioles that are nearly aligned in parallel and has a part of DT that is curved steeply to make a v-shaped contact-point, called the ‘macula densa’, with the renal corpuscle. The efferent arteriole is branched immediately after flowing out the glomerulus, whereas the afferent arteriole is connected directly to a broader vessel, which may reflect the regulatory function of the efferent arteriole. The renal tubule begins at the tubular pole of the renal corpuscle and eventually flows into the connecting duct (Fig. 4b). In the vicinity of the renal corpuscle, the beginning of the PT and a section of the DT in the downstream of the macula densa show remarkably convoluted structures, whereas the other parts of tubules look more straight (left and center panels; Fig. 4a and b). These convoluted tubules may collaborate with the branched efferent arteriole to adjust the contents in the tubular fluid and in blood [33].

## Discussion

Several hours of data collection successfully provided a high-contrast CT image at micron-level resolutions in all directions. This isotropy of resolution is an obvious advantage of X-ray microscopy, which cannot be achieved by serial sectioning. Using a sCMOS detector instead of conventional CCD effectively reduced the acquisition time to 15 min. However, the resolution of optical microscopy (2D method) is around 0.2 micron in general. Brighter X-rays, detector with higher SNR, better algorithm for CT reconstruction would be required to improve resolution and acquisition time of X-ray microscopy in future. In this study, we measured 25 different nephrons for both CCD (Supplementary Fig. S1) and sCMOS (Supplementary Fig. S2) detectors for histogram analysis, which confirmed that the PTs and DTs were distinguishable on the basis of their X-ray opacities. Therefore we believe that this method can cover enough number of measurements for diagnostic purposes.

In the CT image obtained, part of a single nephron connected to a renal corpuscle was segmented to produce a 3D rendering model that covered a maximum 40% of the entire tubule. In terms of overall spatial arrangement of nephron parts, the model obtained by the laboratory-based X-ray CT was consistent with the previous reports of nephron models segmented from synchrotron-based X-ray CT images [23] or those reconstructed by serial sectioning from optical microscope images [6,7]. Compared to the previous reports, the present model described more detailed features of the nephron, including a convoluted structure of renal tubules neighboring the renal corpuscle and a branched structure of efferent arterioles. These results confirmed the capability of nano3DX to visualize 3D structure of mouse renal biopsy at micron-level resolutions. However, recently, serial electron microscopy provides much finer 3D structures such as glomerular capillary network [34] and glomerular endothelial cells [35]. Therefore, main benefits of laboratory-based X-ray microscopy would be faster acquisition of nondestructive 3D structures with better user accessibility. In addition, another attractive application of X-ray microscopy may be volumetric analysis of whole kidney [36,37], although it is not suitable for the analysis of individual nephron, because of its huge number in the kidney. Limited numbers of nephron in a renal biopsy may allow finer analysis of individual nephron.

In this work, high CNRs due to the osmium staining that effectively enhanced the X-ray absorption contrast allowed us to extract manually a single mouse nephron through area segmentation around a renal corpuscle; an automatic segmentation using a process such as the active contour method [38] or deep learning [39] may be possible in near future, since the automatic segmentation at much lower CNRs of about 1.5 was reported in the case of maize embryo [40]. However, it should be noted that osmium tetroxide has a preference for unsaturated bonds in biological materials [32]. For instance, in the X-ray CT image of the osmium-stained kidney, the lumen side of PT looks dark, as if it were a void (Fig. 3c and e), although a brush border should exist in the PT lumen. It is conceivable that the brush border of the tubule is less reactive with osmium tetroxide compared to the PT wall. A possible way to solve this reactivity problem in X-ray microscopy may be using unstained paraffin-embedded samples although their contrast are much lower when compared to osmium-stained samples. In addition to the contrast enhancement ability, notable advantage of the osmium staining is its compatibility with electron microscopy, which routinely uses osmium tetroxide as a chemical-fixing reagent. Thus, utilizing the nondestructive characteristic of X-ray CT, the sample can be reused after X-ray microscopy observation for a further observation by electron microscopy at nanometer-scale resolution. The sample can also be reused for optical microscopy, provided that osmium tetroxide is replaced by other staining agents such as iodine. A selective heavy-atom staining aimed at a specific kidney compartment [41] may be considered also.

## Conclusion

Microscopic observations of biological structures are indispensable not only for research in life sciences but also for medical applications. To date, bio-specimens have been observed mainly using optical microscopy and electron microscopy. Herein we reported the application of ‘nano3DX’, a compact, commercially available, laboratory-based X-ray microscope (Rigaku, Tokyo, Japan) through the high-resolution 3D observation of an osmium-stained mouse kidney piece embedded in an elongated resin sheet as a model of renal biopsy. Laboratory-based X-ray microscopy would be a key technique in a multimodal correlative microscopy, enabling a wide range of seamless visualization from millimeter to nanometer scales through a complementary use of various observation modalities. This multimodal correlative microscopy may provide a powerful basis for histomorphology and/or clinical pathology in future.

## Supplementary Material

dfac033_Supp
