# Supplementary material for: Compact laboratory-based X-ray microscope enabling nondestructive 3D structure acquisition of mouse nephron with high speed and better user accessibility
Source: Microscopy (Oxf). 2022 Jul 2;71(6):315–23. doi: 10.1093/jmicro/dfac033 (PMC9731380; doi:10.1093/jmicro/dfac033)

Supplementary Fig. 1. CNR measurement using the program *ImageJ* for CCD data.

Proximal tubule measurement for 1_300_M_t nephron 1: air (top) and material (bottom) regions.

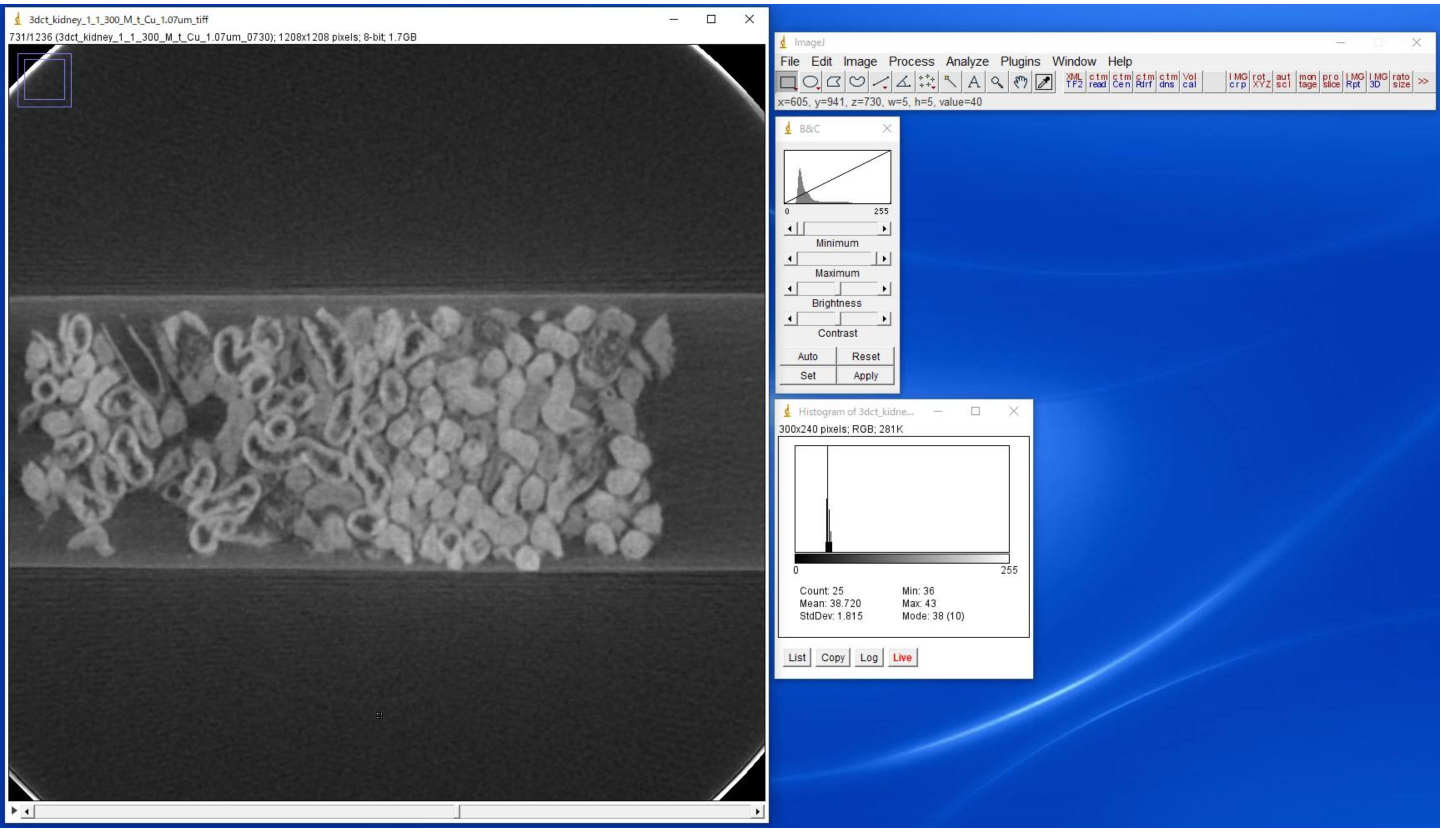

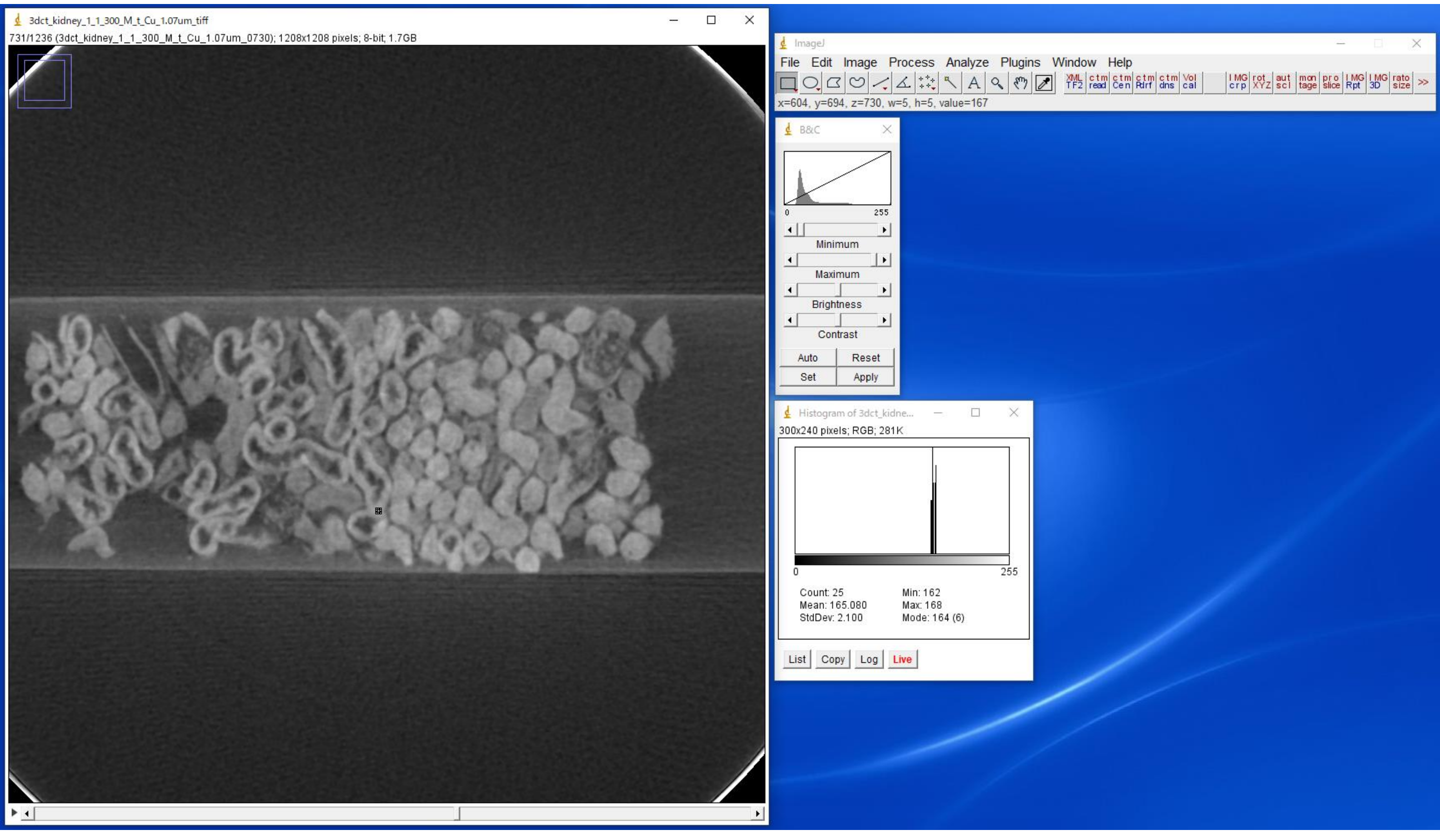

Supplementary Fig. 1. CNR measurement using the program *ImageJ* for CCD data.

Distal tubule measurement for 1_300_M_t nephron 1: air (top) and material (bottom) regions.

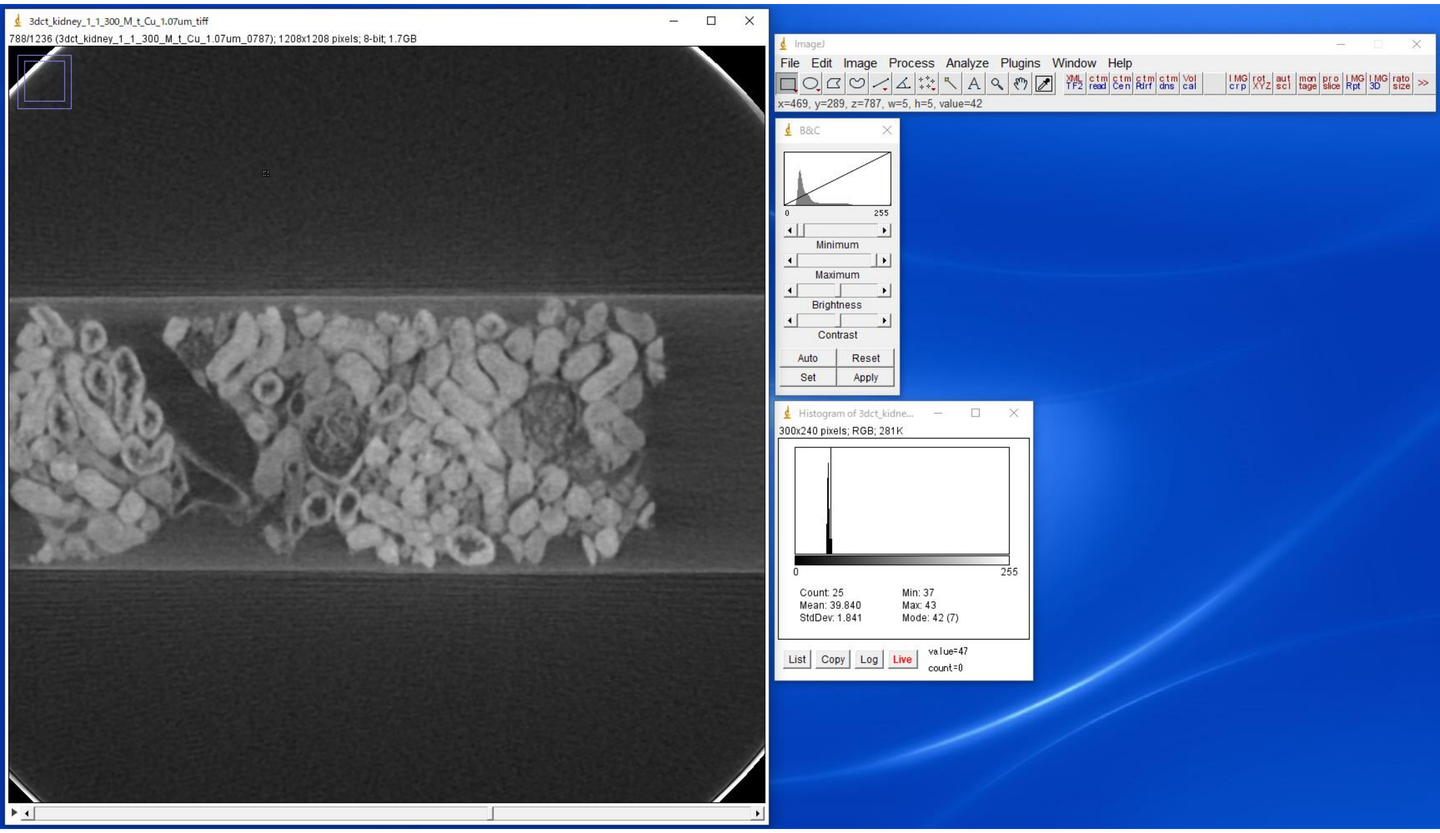

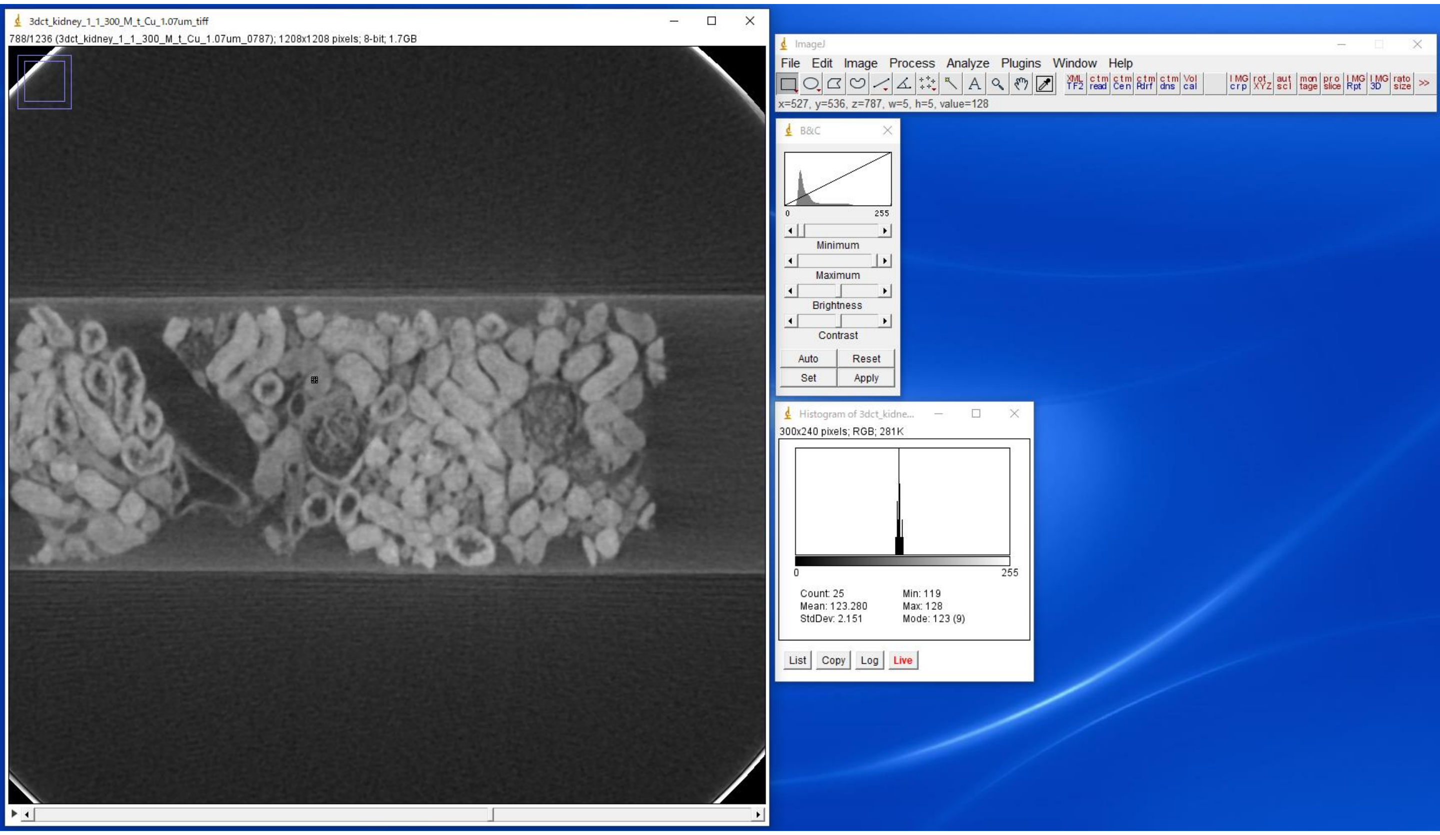

Supplementary Fig. 1. CNR measurement using the program *ImageJ* for CCD data.

Proximal tubule measurement for 1_300_M_t nephron 2: air (top) and material (bottom) regions.

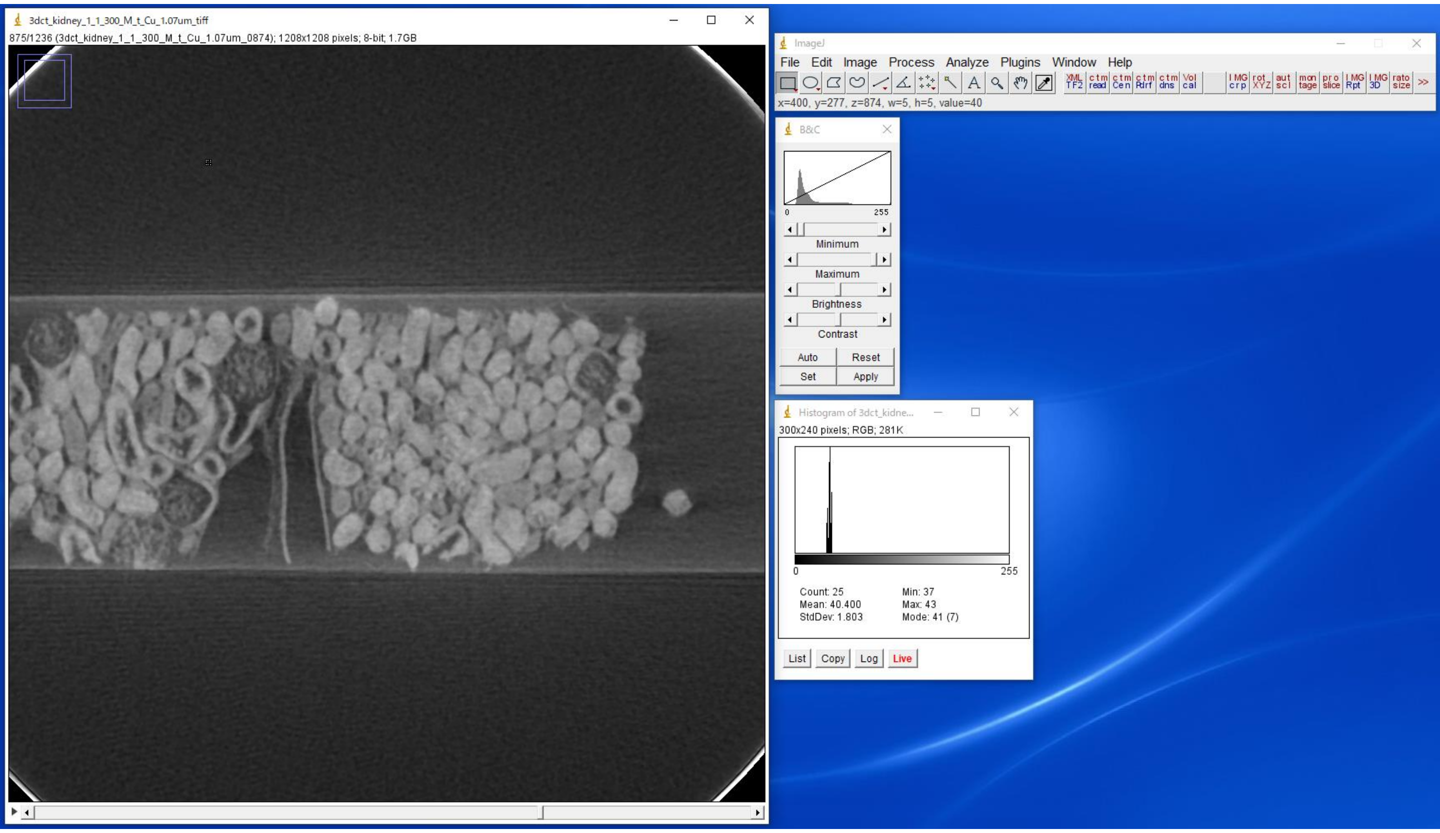

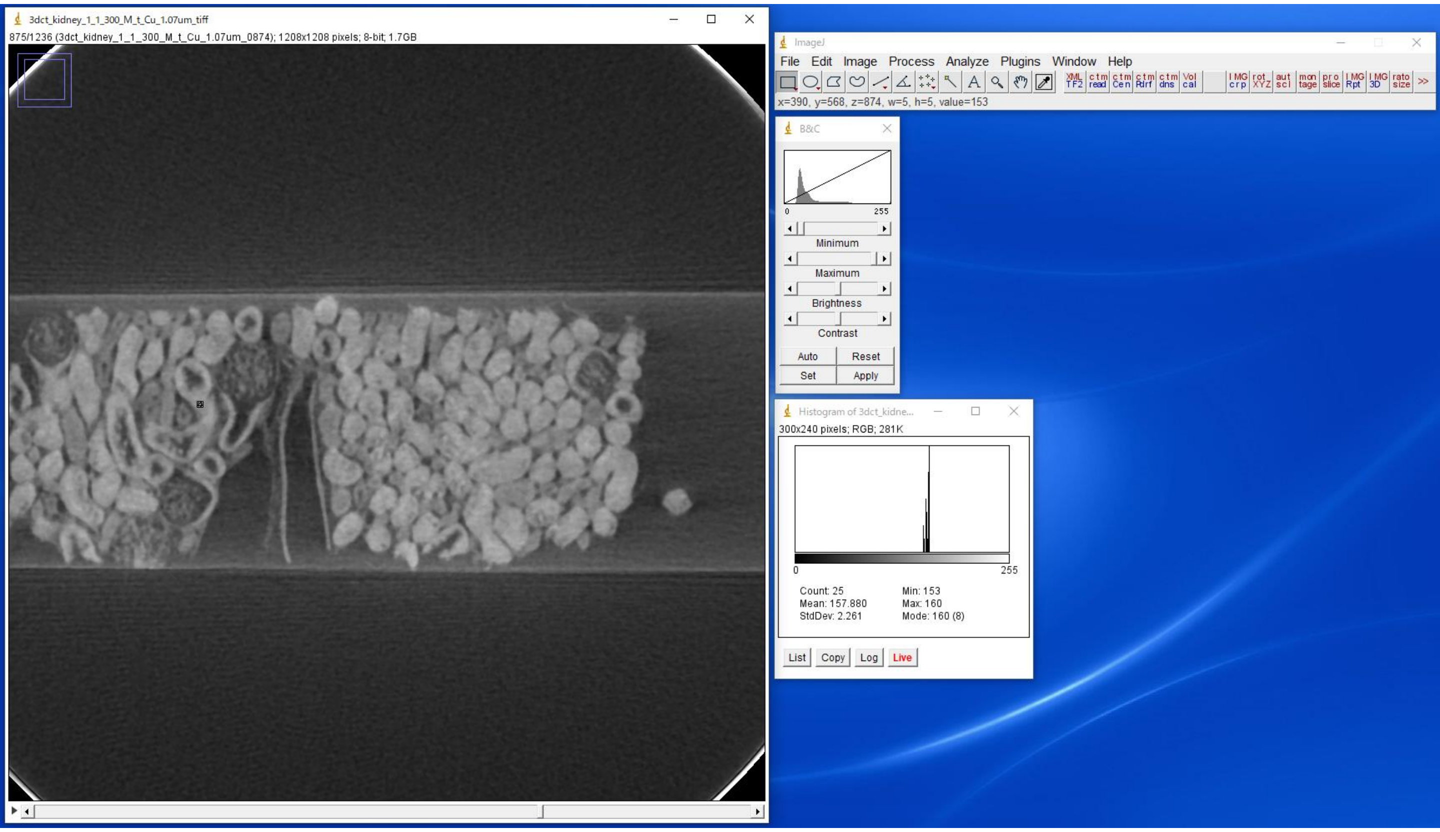

Supplementary Fig. 1. CNR measurement using the program *ImageJ* for CCD data.

Distal tubule measurement for 1_300_M_t nephron 2: air (top) and material (bottom) regions.

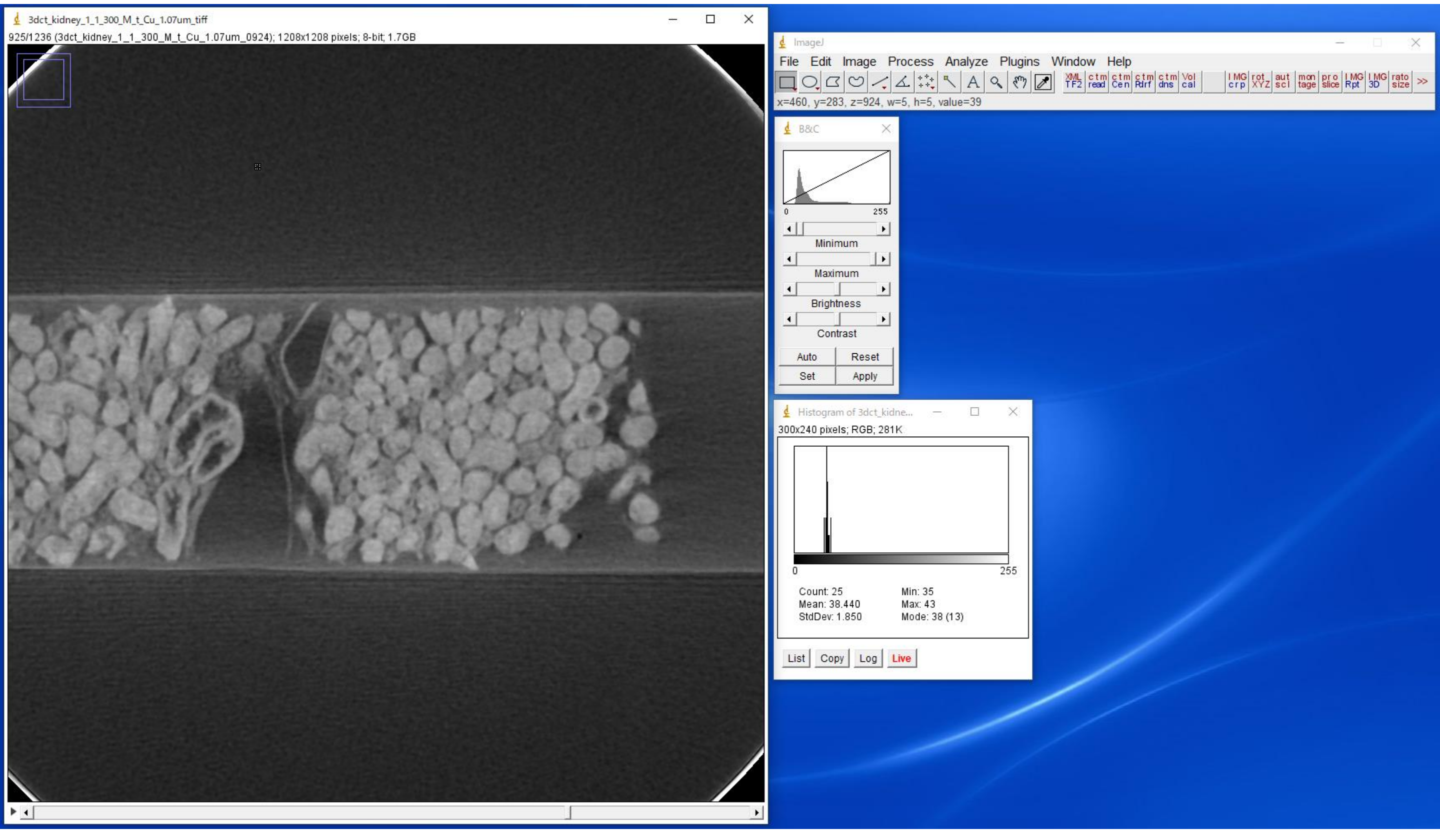

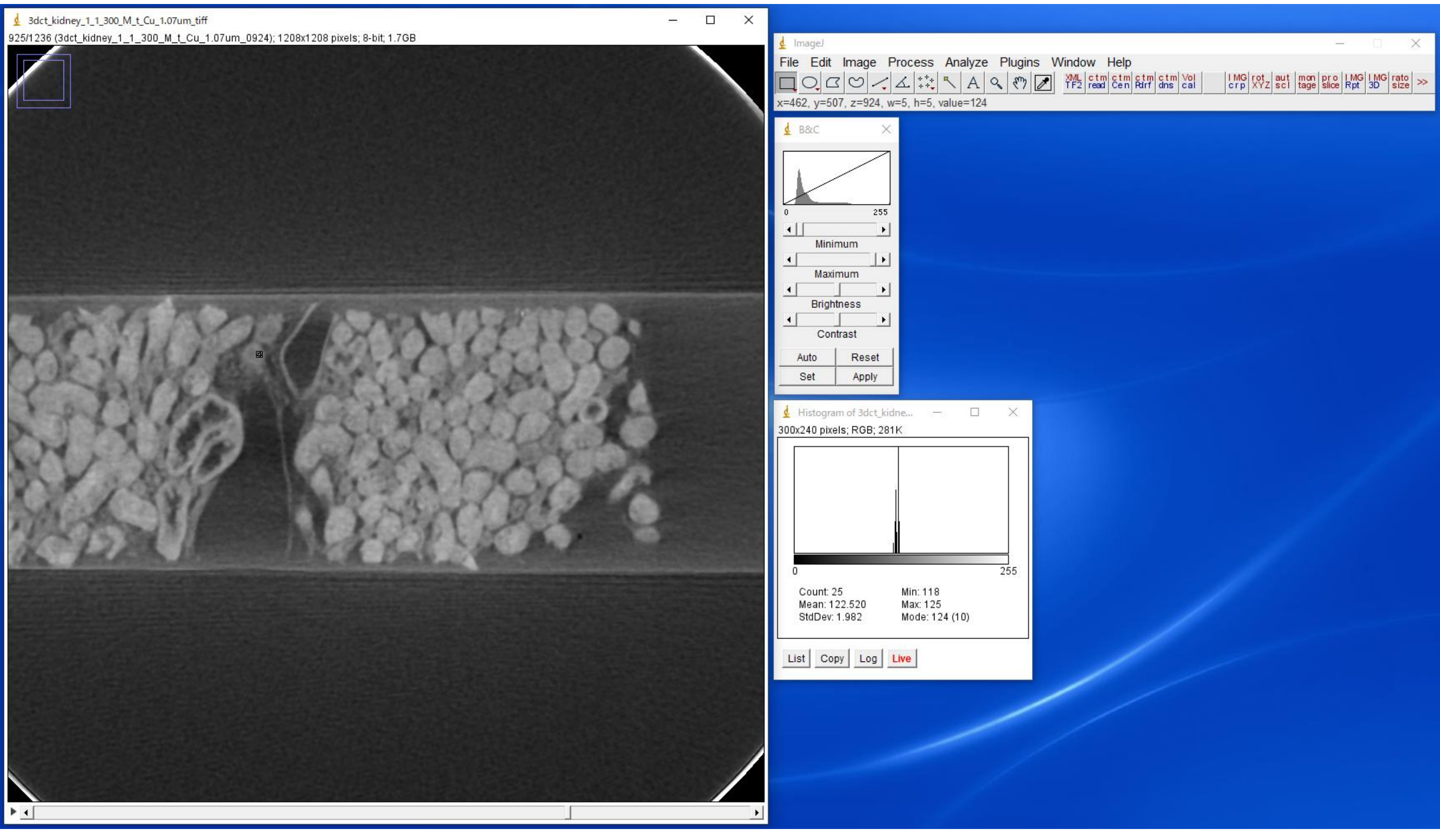

Supplementary Fig. 1. CNR measurement using the program *ImageJ* for CCD data.

Proximal tubule measurement for 1_300_M_t nephron 3: air (top) and material (bottom) regions.

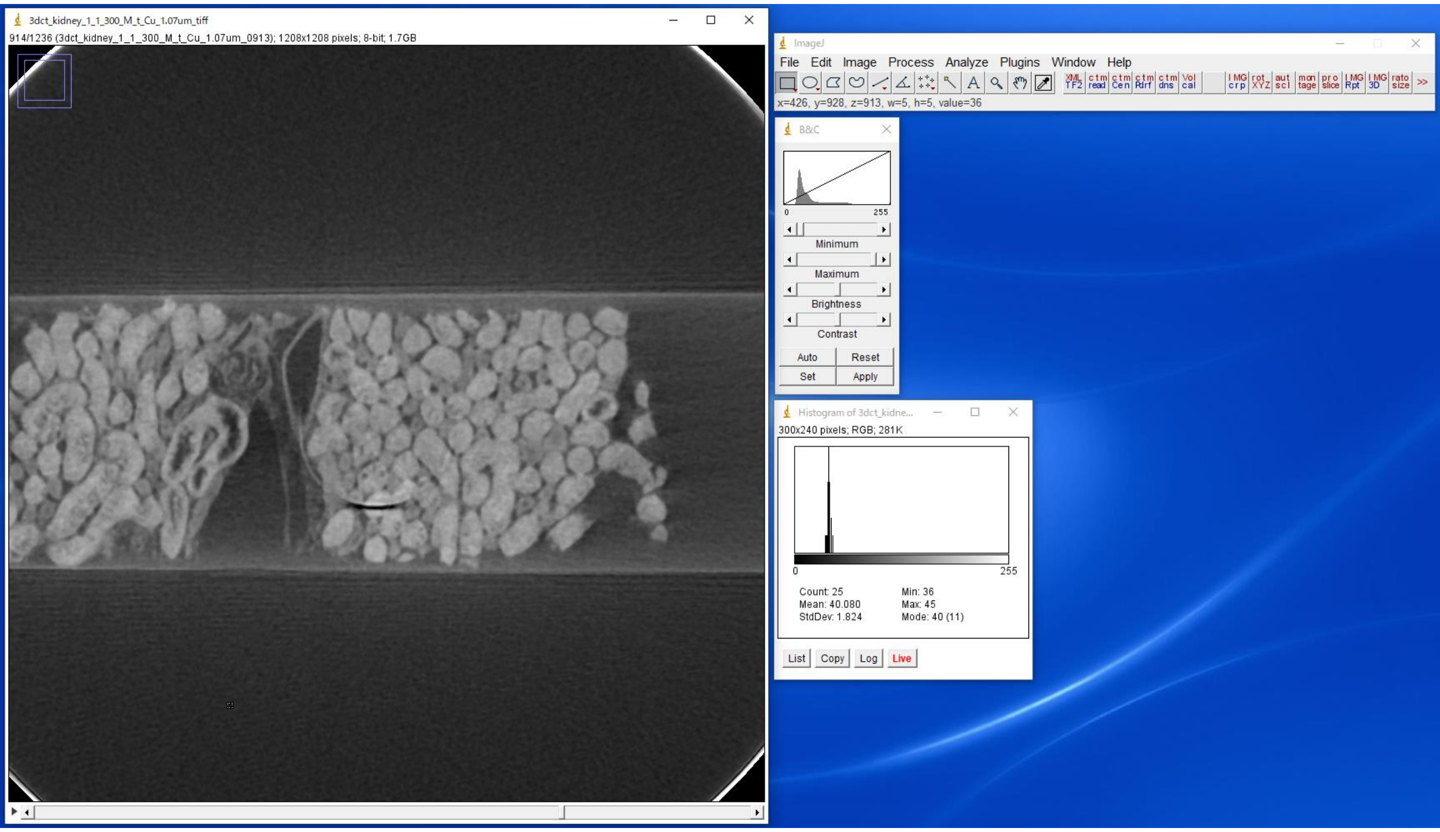

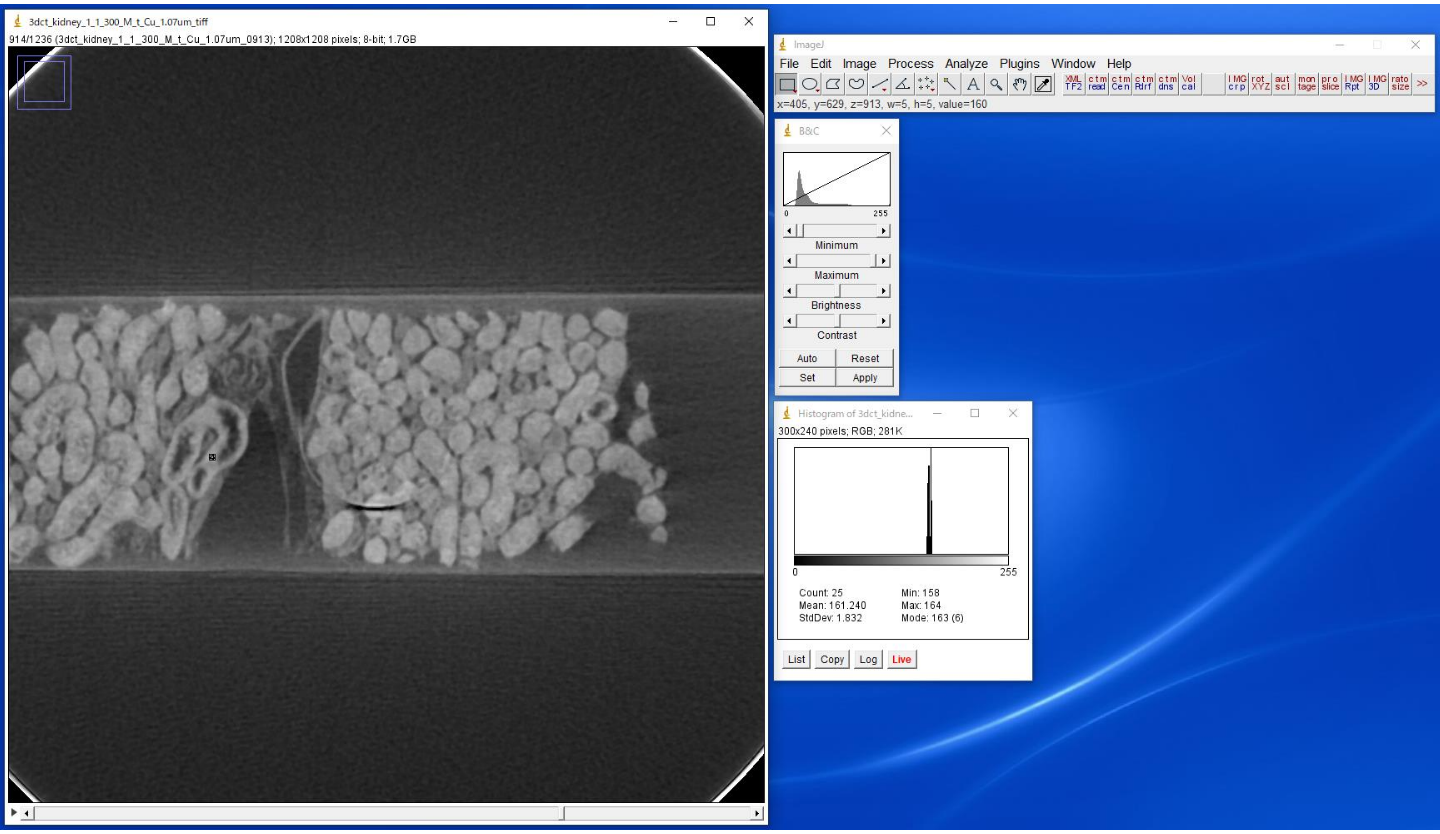

Supplementary Fig. 1. CNR measurement using the program *ImageJ* for CCD data.

Distal tubule measurement for 1_300_M_t nephron 3: air (top) and material (bottom) regions.

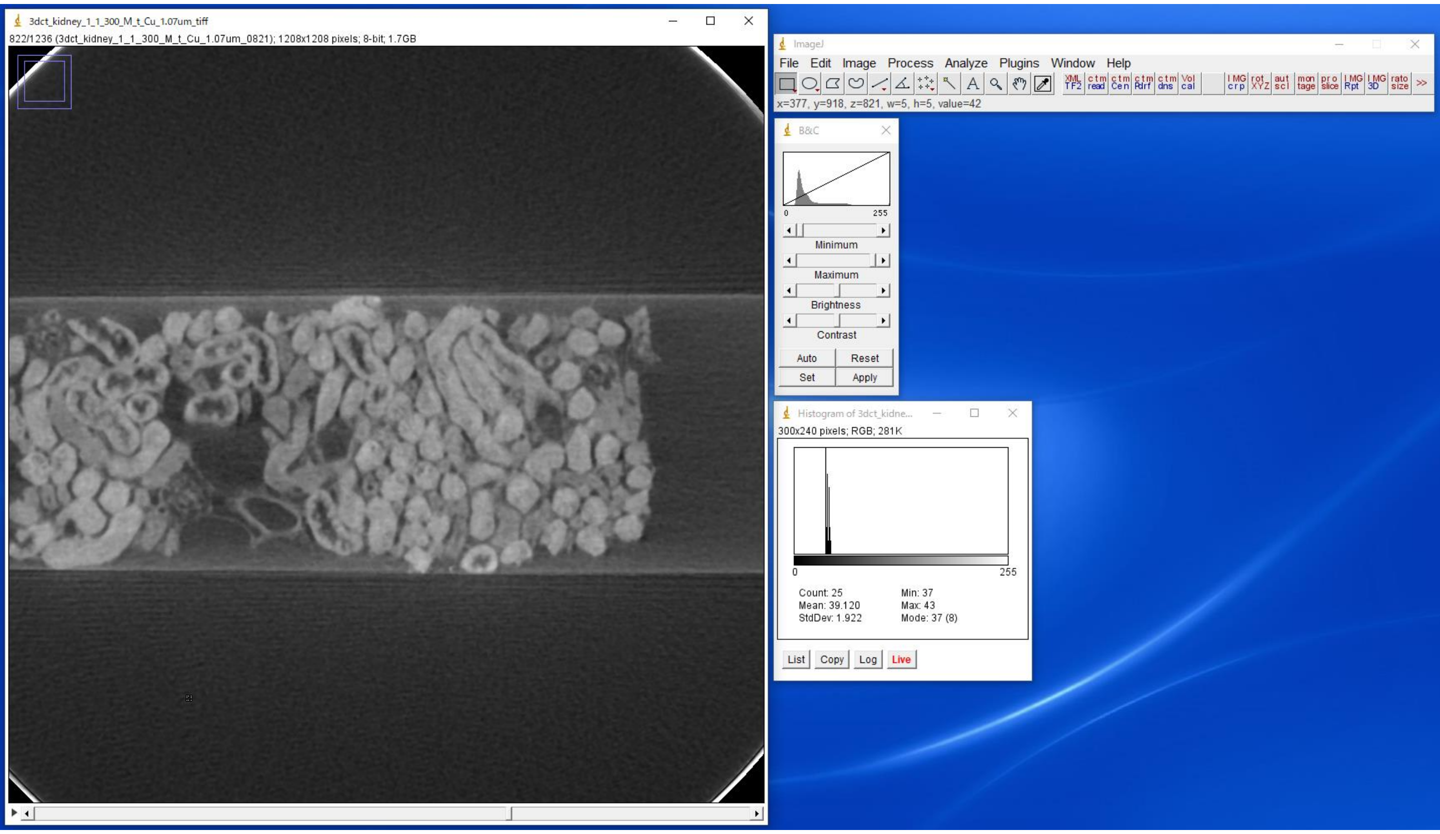

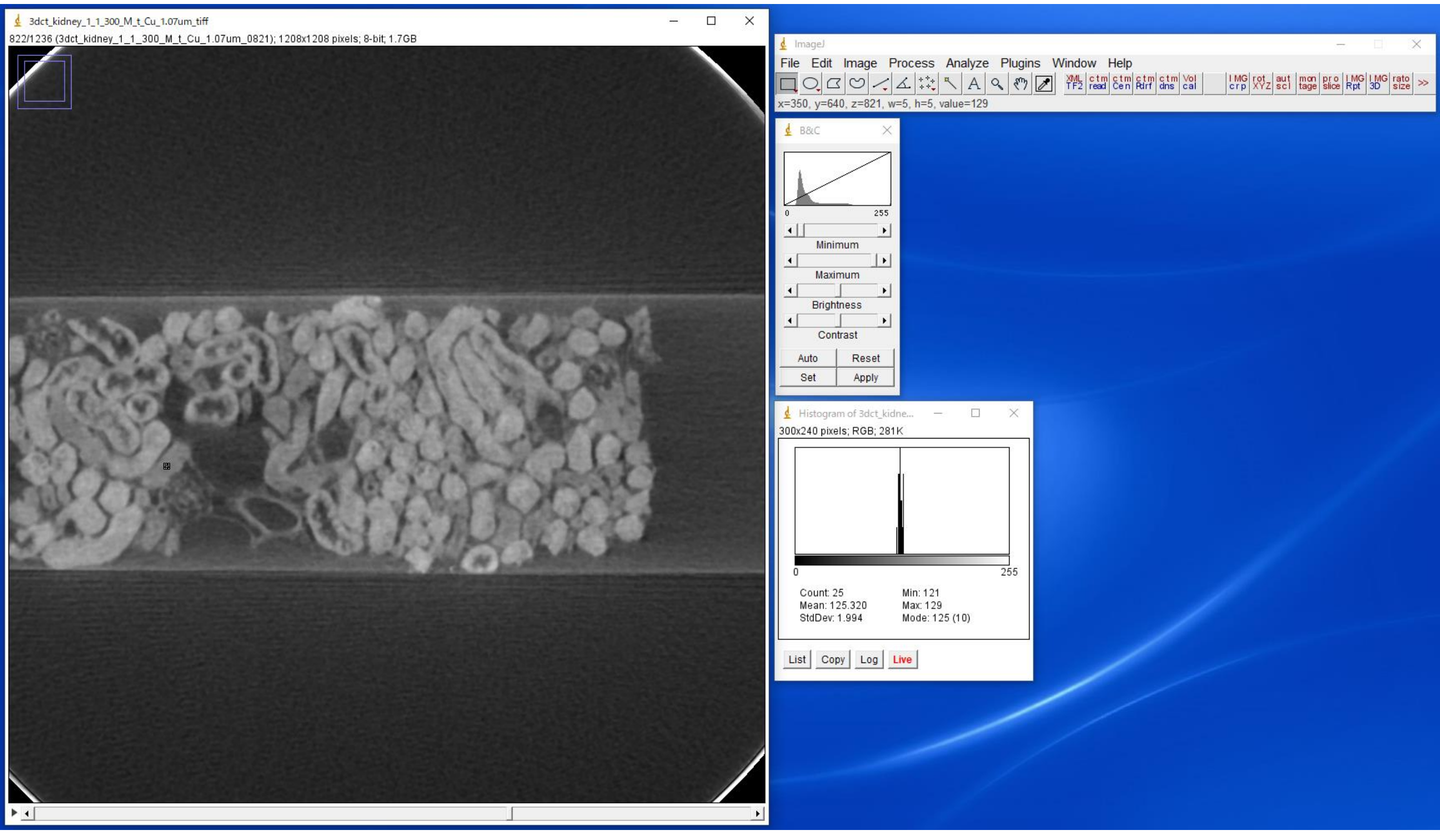

Supplementary Fig. 1. CNR measurement using the program *ImageJ* for CCD data.

Proximal tubule measurement for 1_300_M_t nephron 4: air (top) and material (bottom) regions.

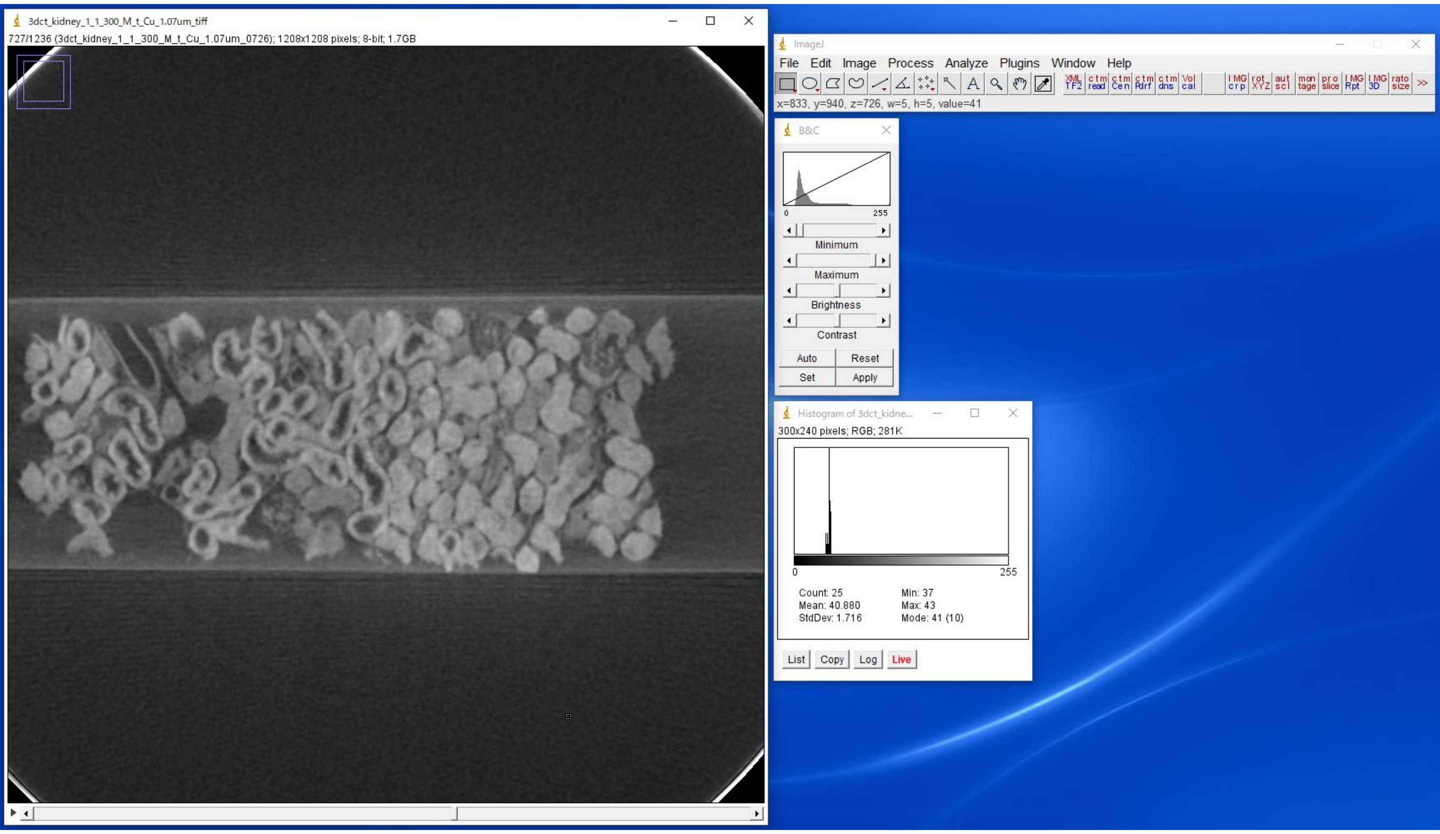

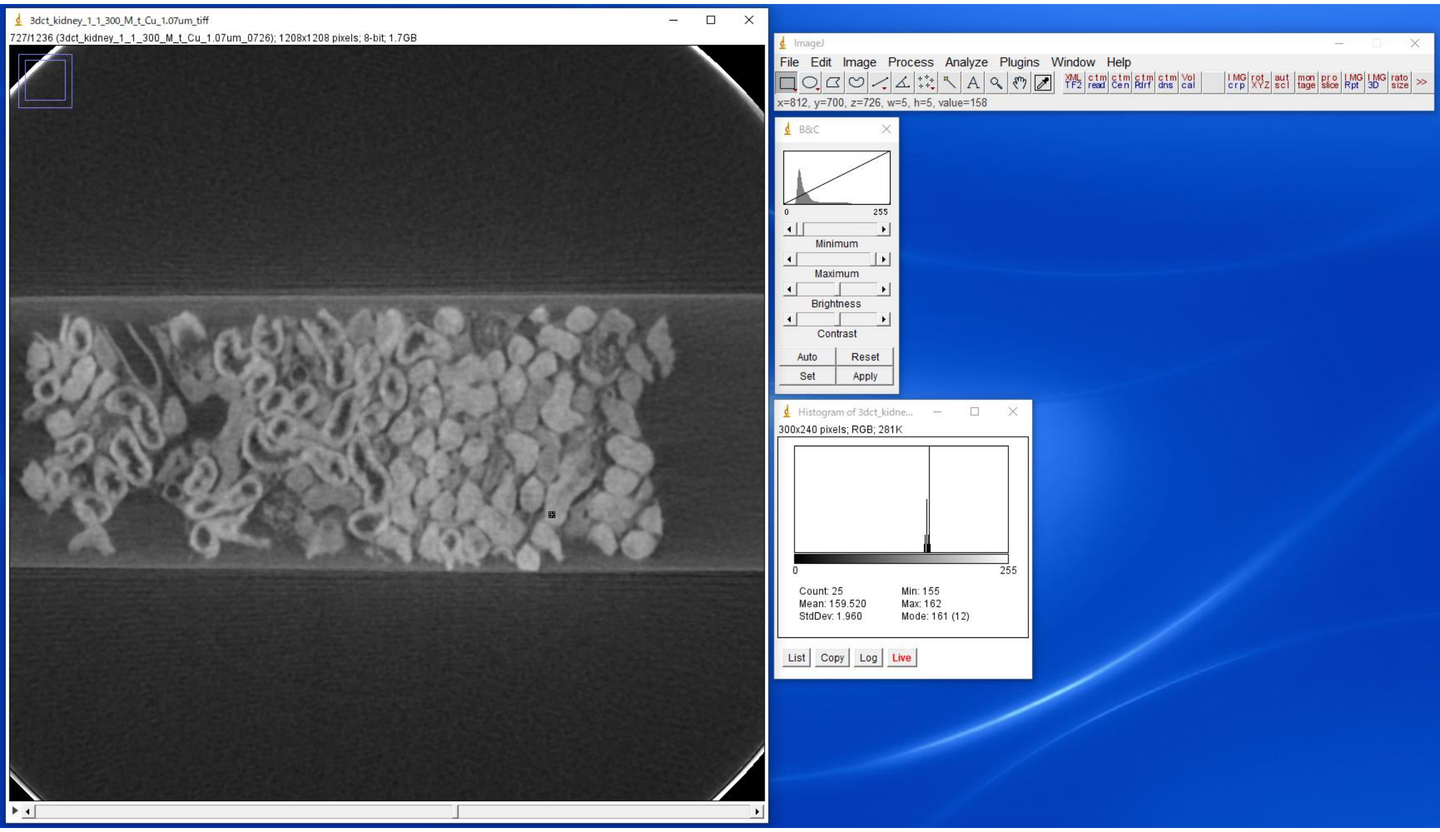

Supplementary Fig. 1. CNR measurement using the program *ImageJ* for CCD data.

Distal tubule measurement for 1_300_M_t nephron 4: air (top) and material (bottom) regions.

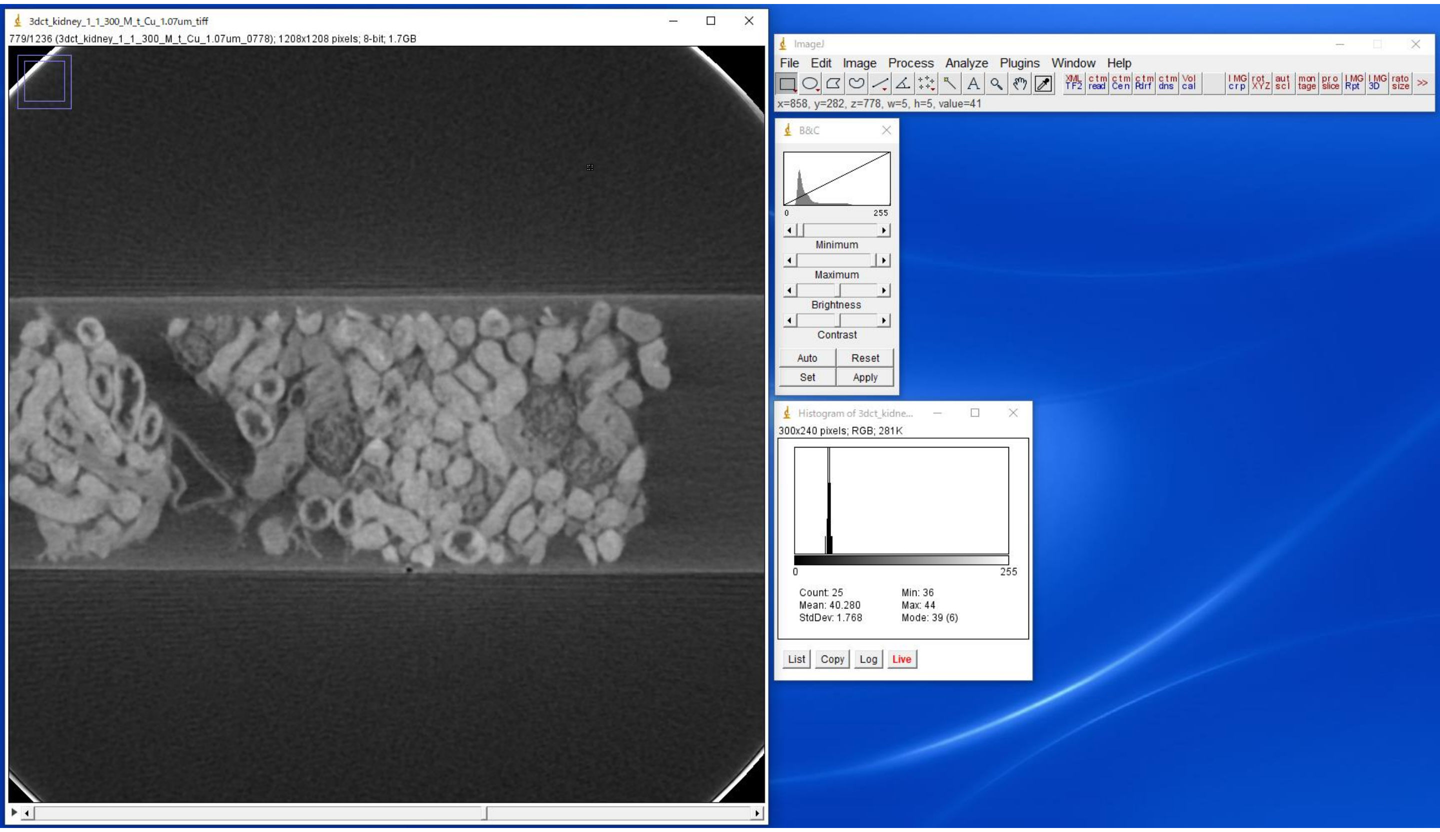

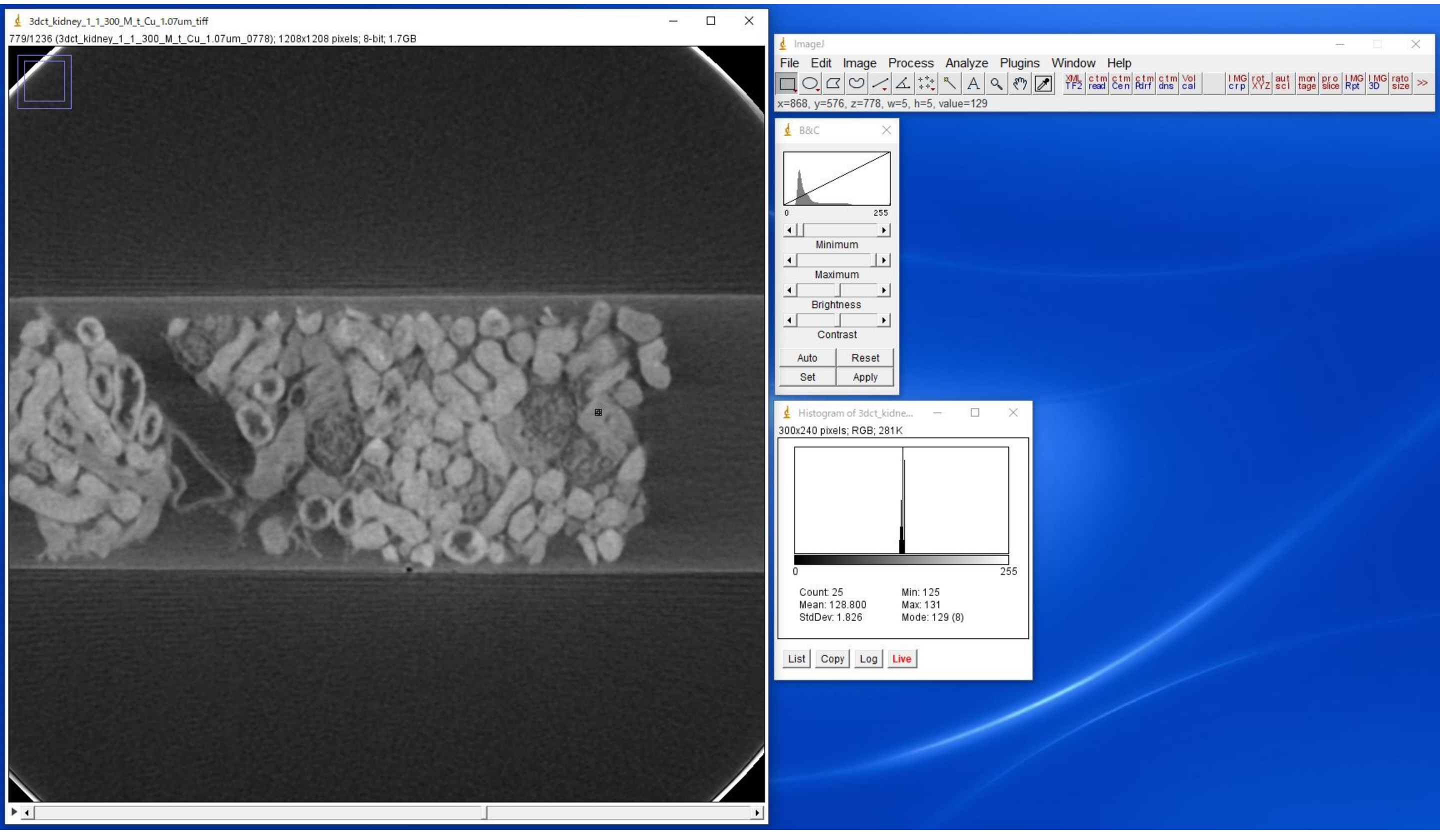

Supplementary Fig. 1. CNR measurement using the program *ImageJ* for CCD data.

Proximal tubule measurement for 1_300_M_t nephron 5: air (top) and material (bottom) regions.

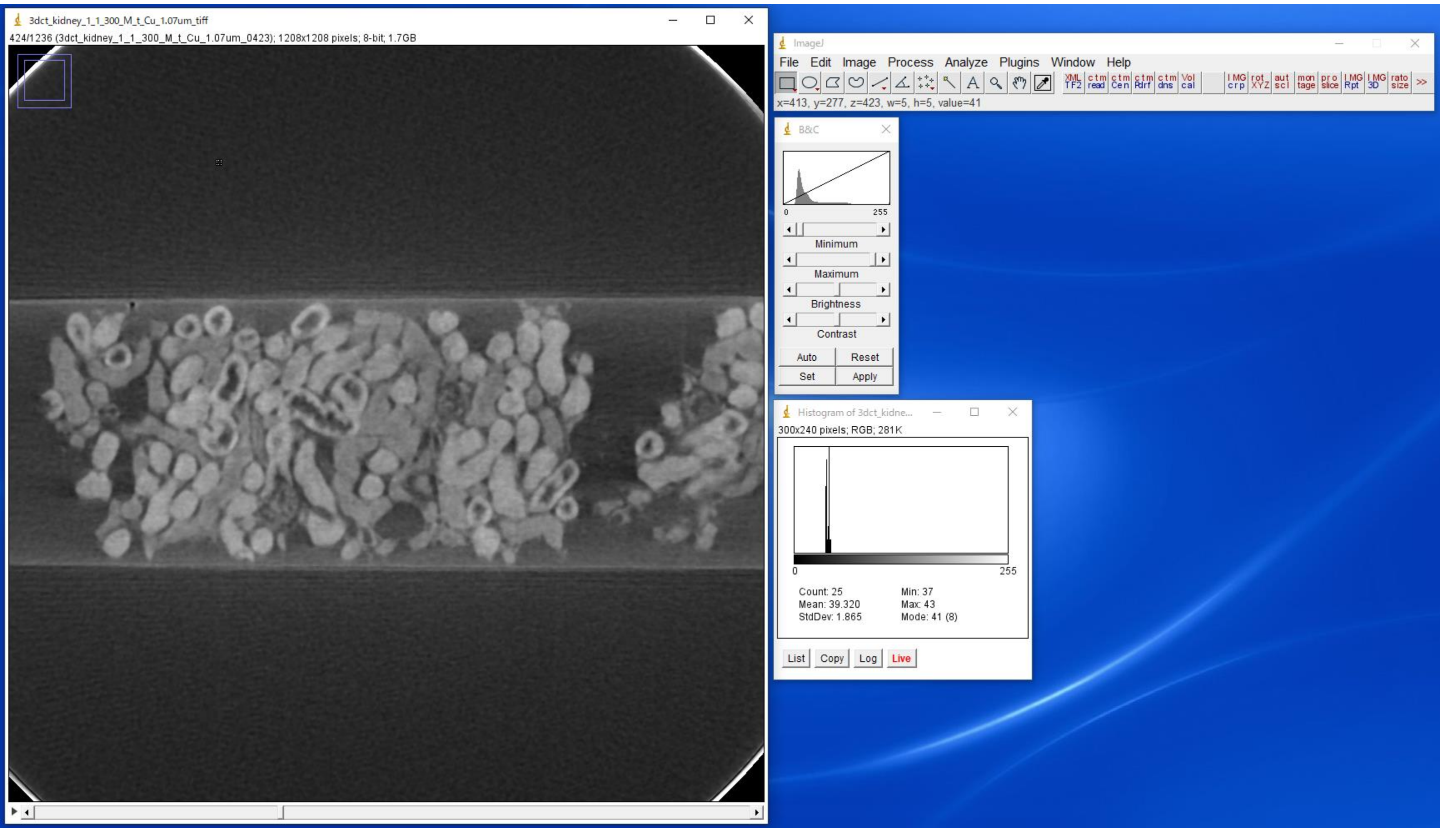

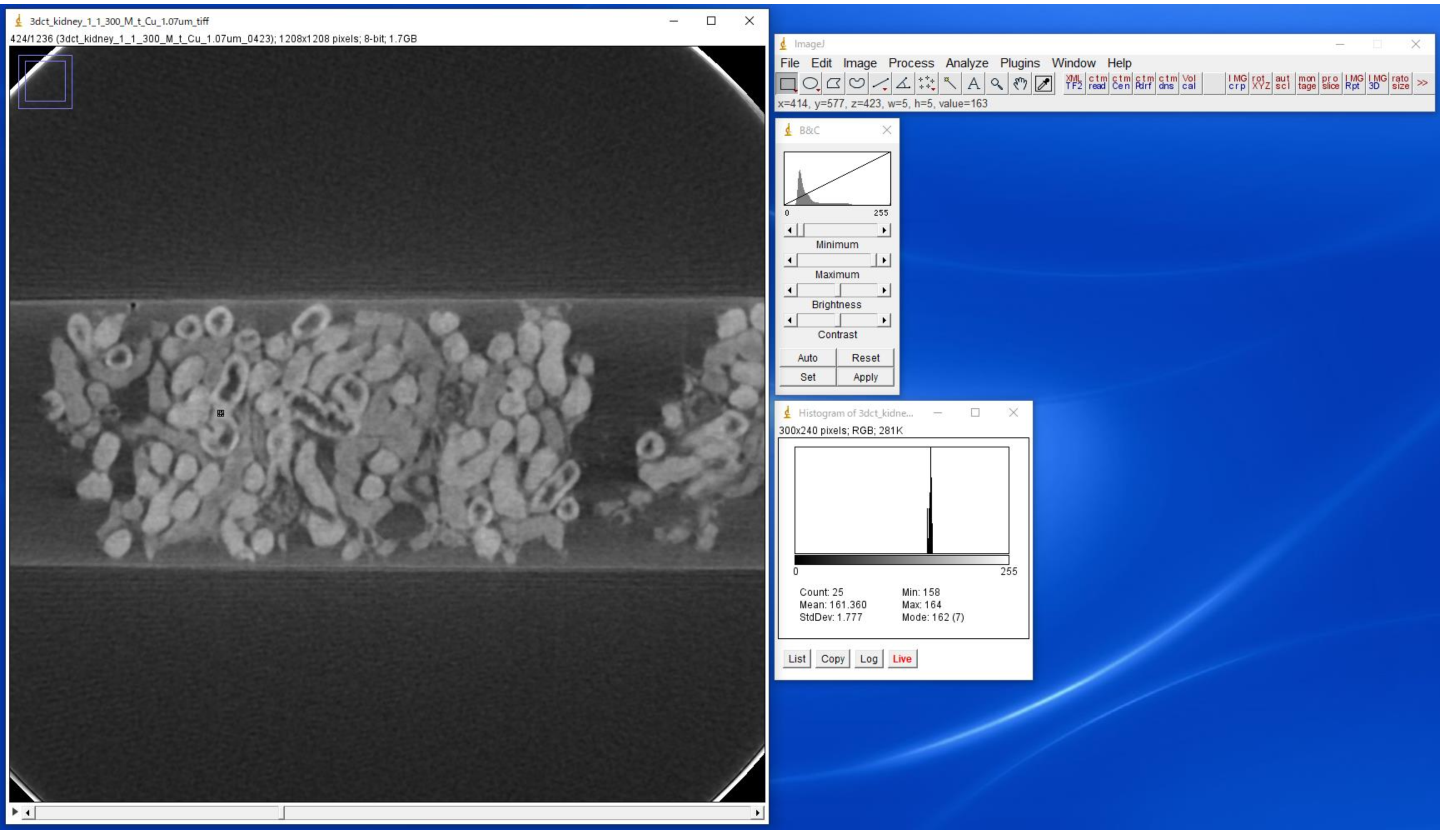

Supplementary Fig. 1. CNR measurement using the program *ImageJ* for CCD data.

Distal tubule measurement for 1_300_M_t nephron 5: air (top) and material (bottom) regions.

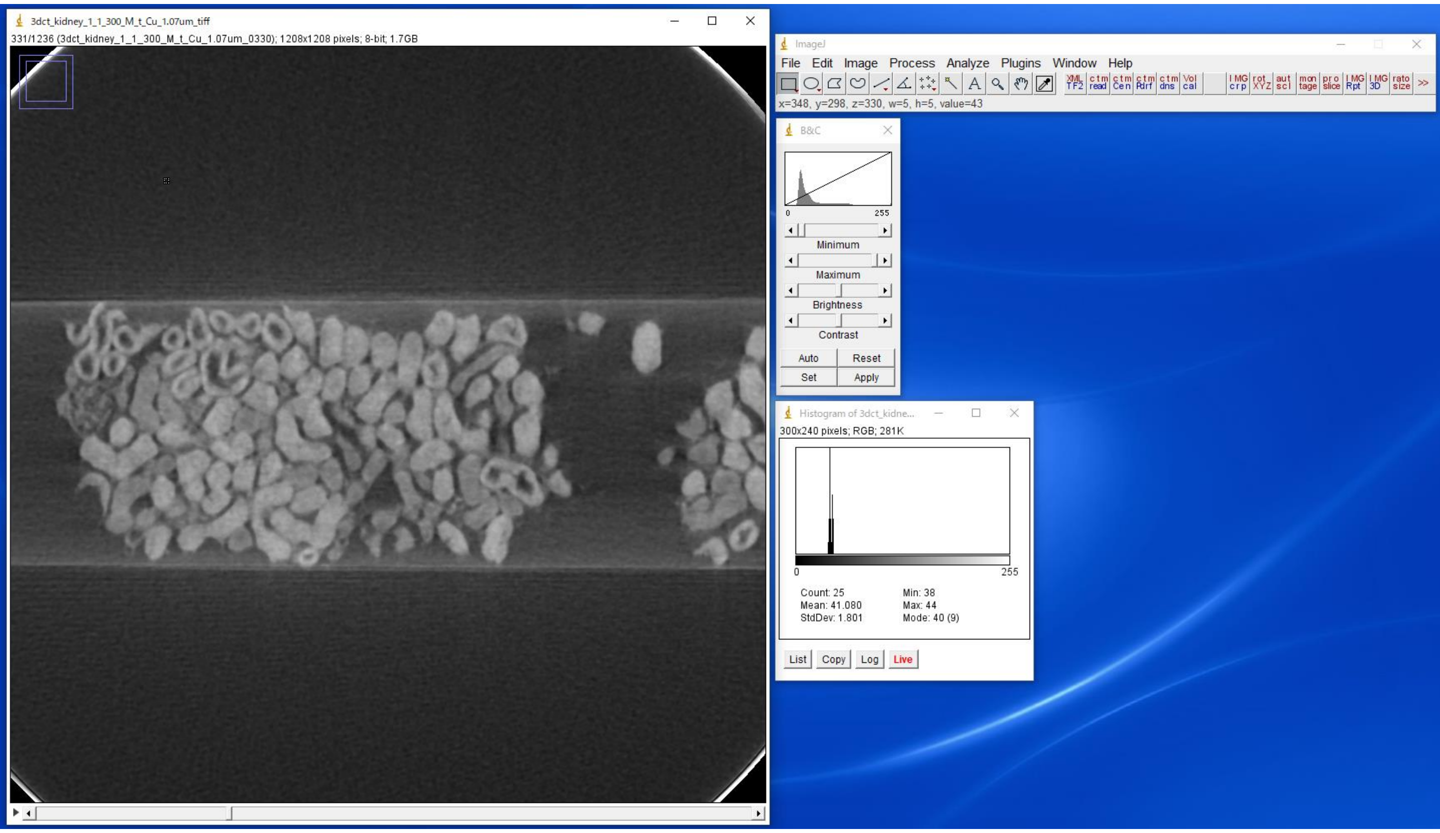

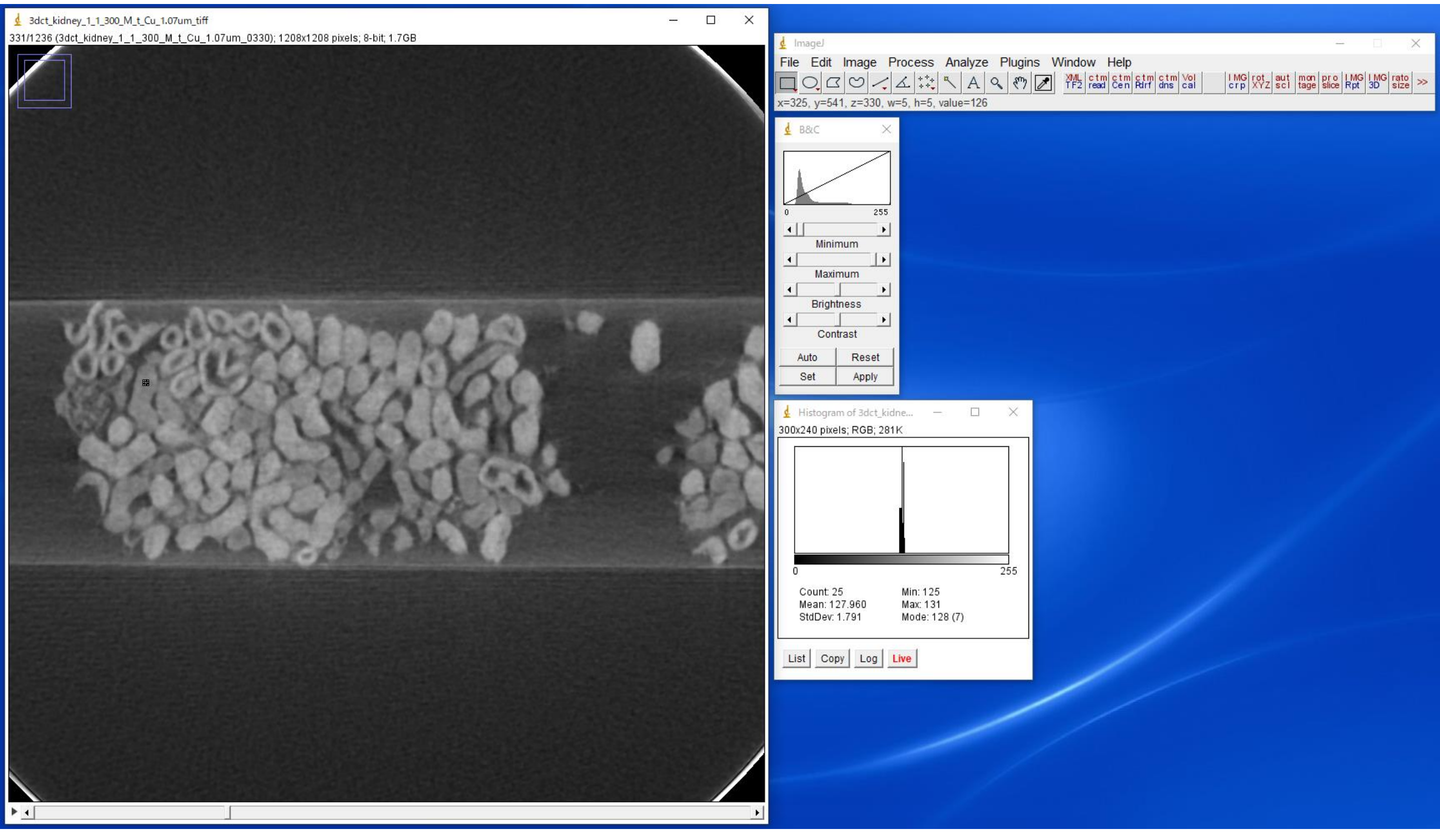

Supplementary Fig. 1. CNR measurement using the program *ImageJ* for CCD data.

Proximal tubule measurement for 2_500_M_t nephron 1: air (top) and material (bottom) regions.

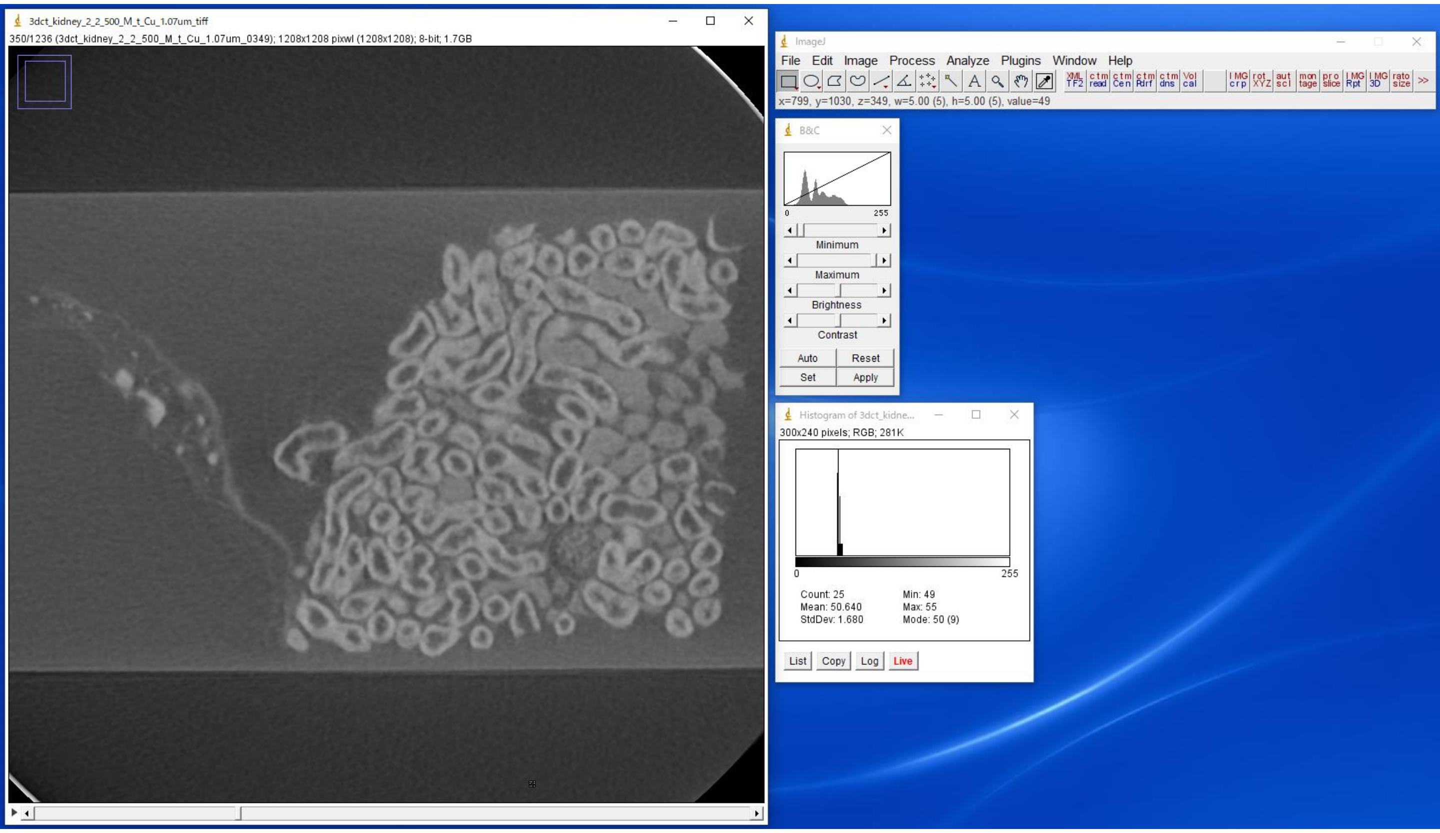

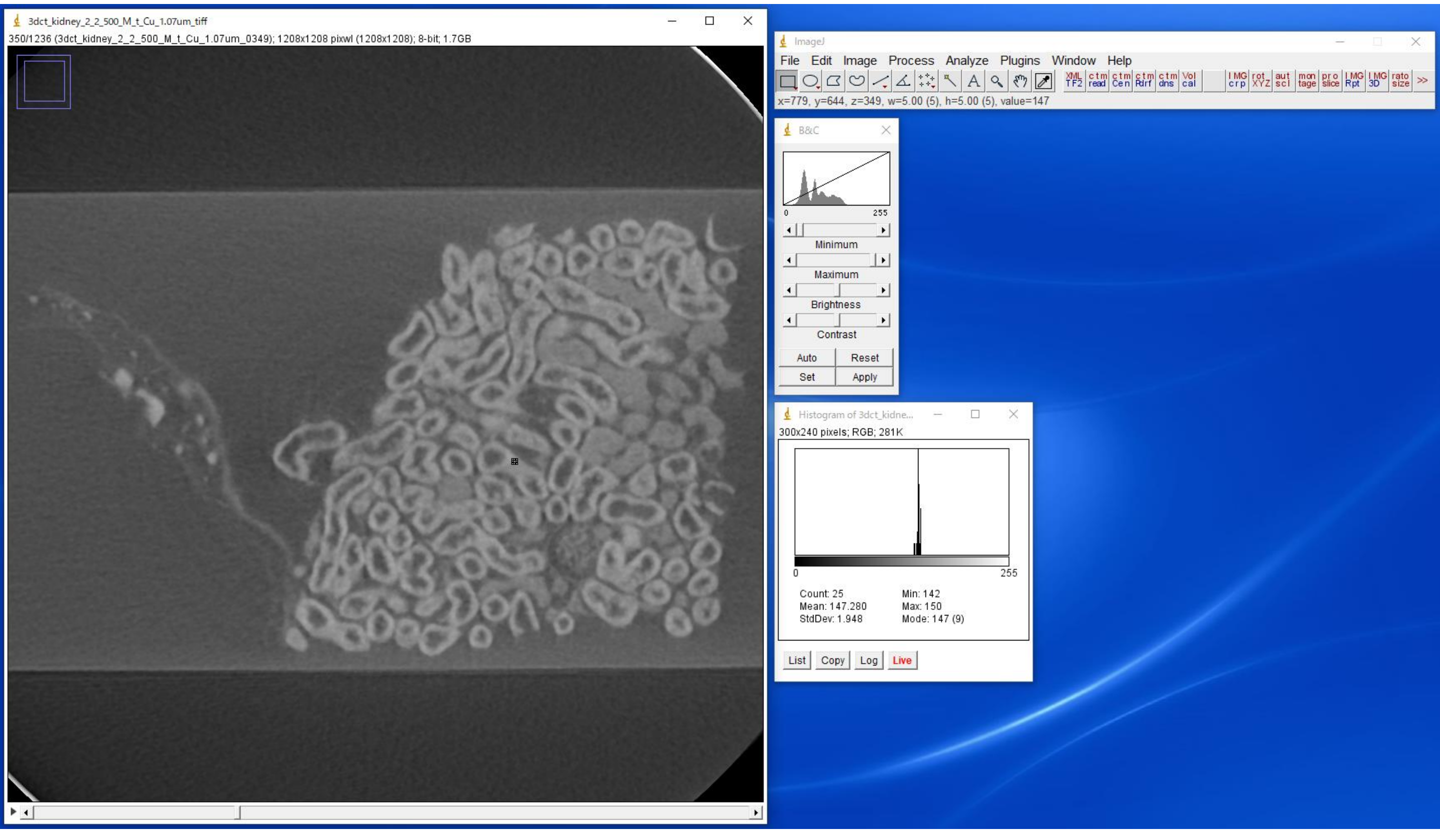

Supplementary Fig. 1. CNR measurement using the program *ImageJ* for CCD data.

Distal tubule measurement for 2_500_M_t nephron 1: air (top) and material (bottom) regions.

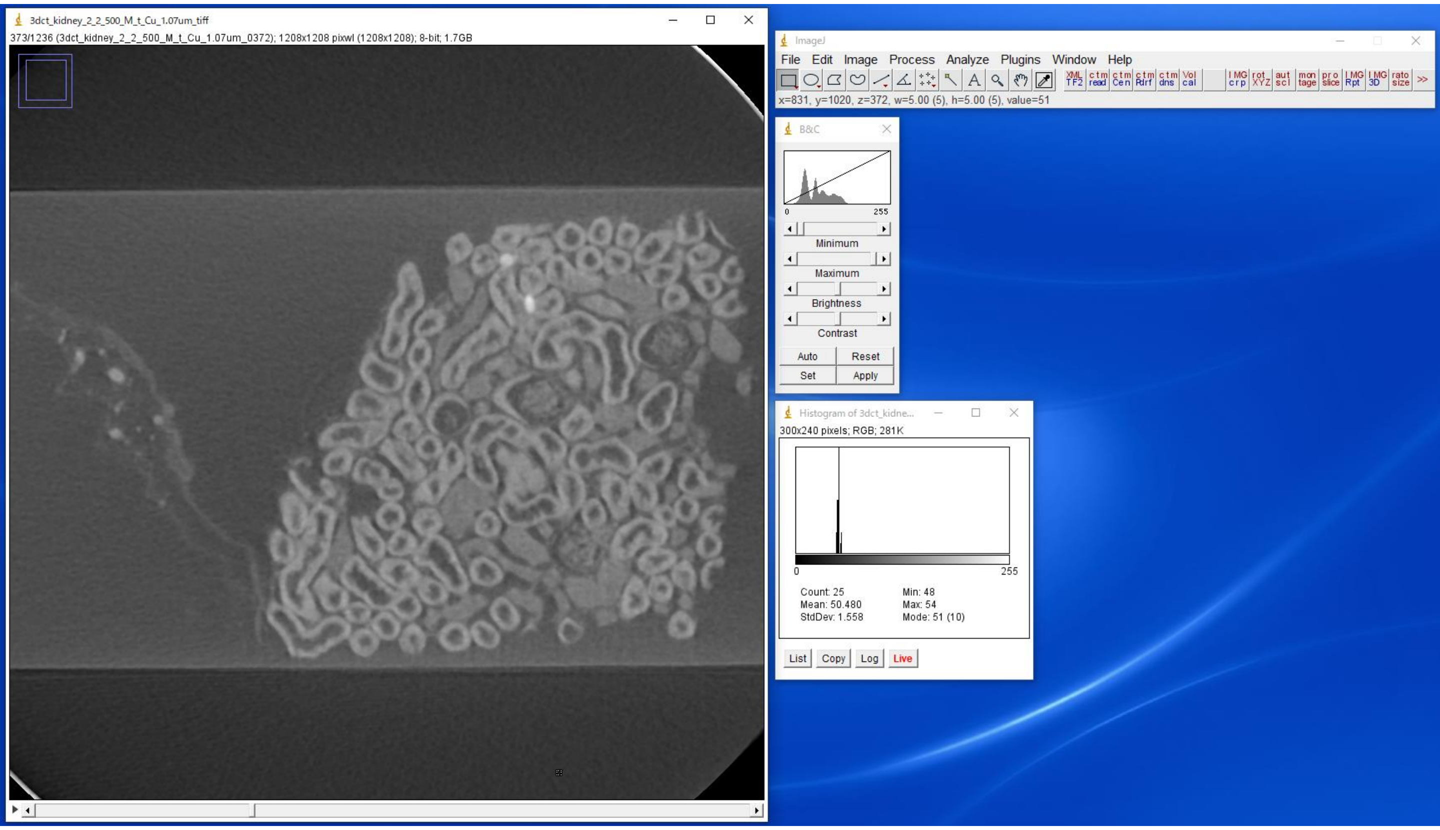

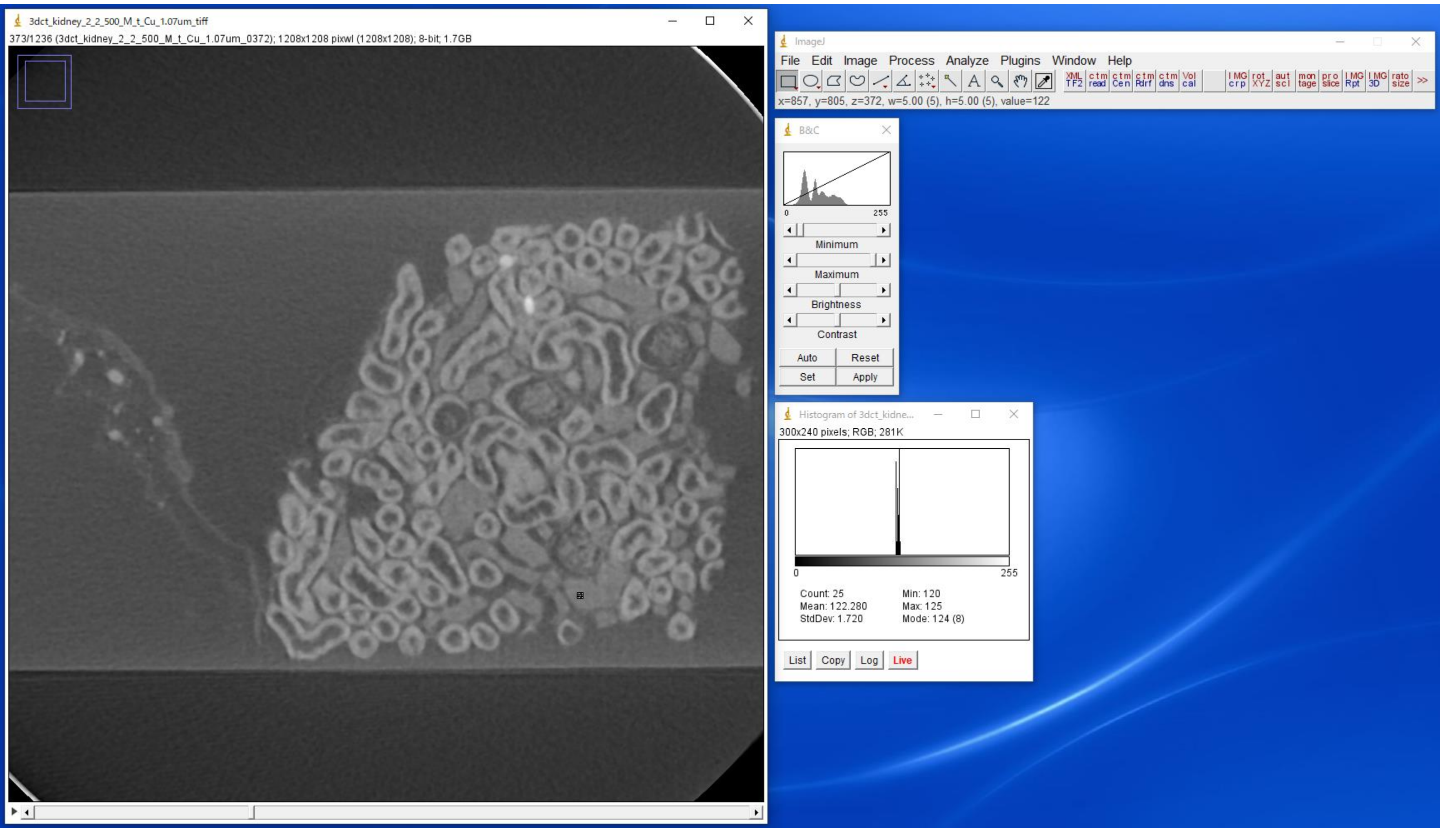

Supplementary Fig. 1. CNR measurement using the program *ImageJ* for CCD data.

Proximal tubule measurement for 2_500_M_t nephron 2: air (top) and material (bottom) regions.

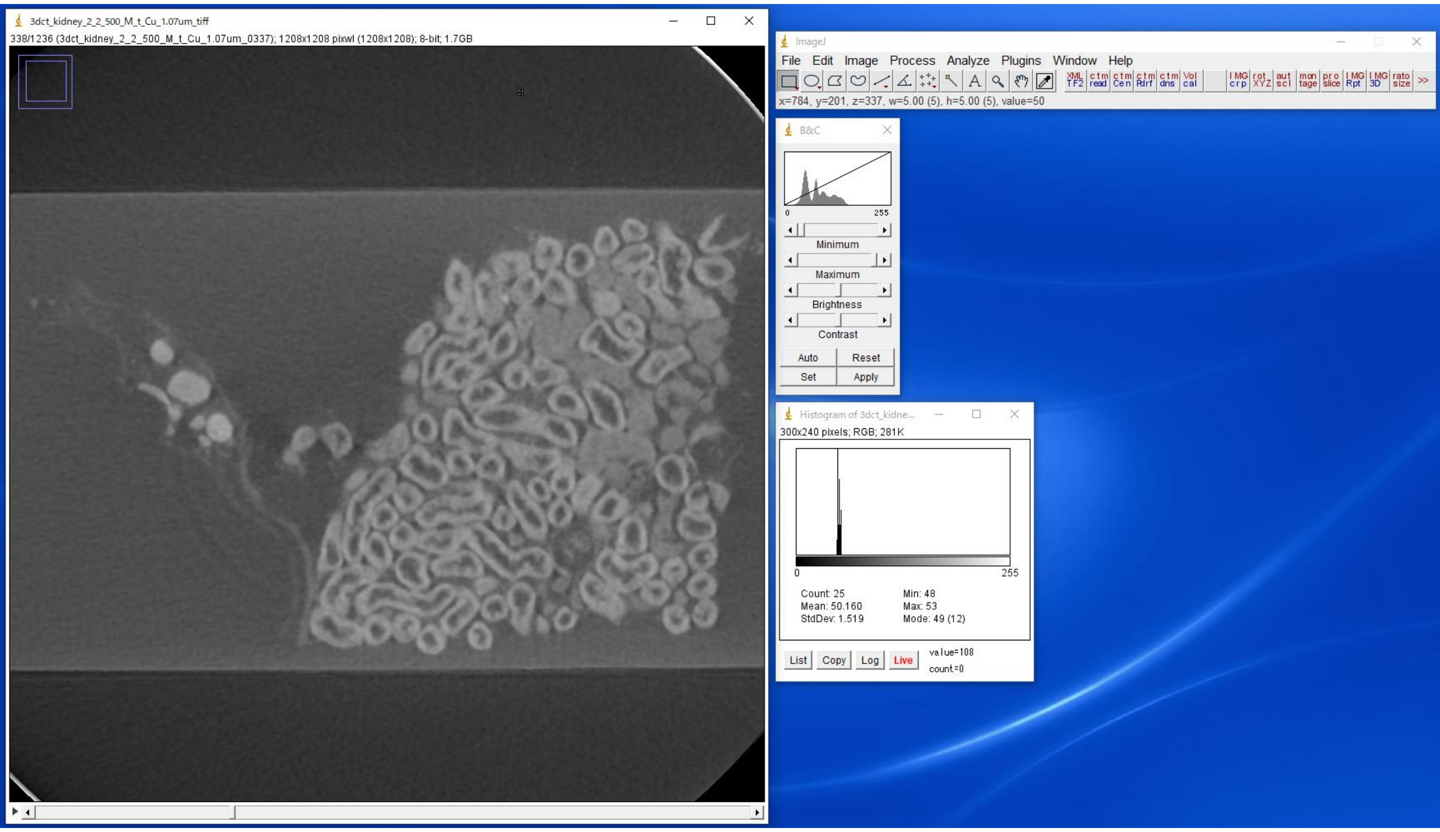

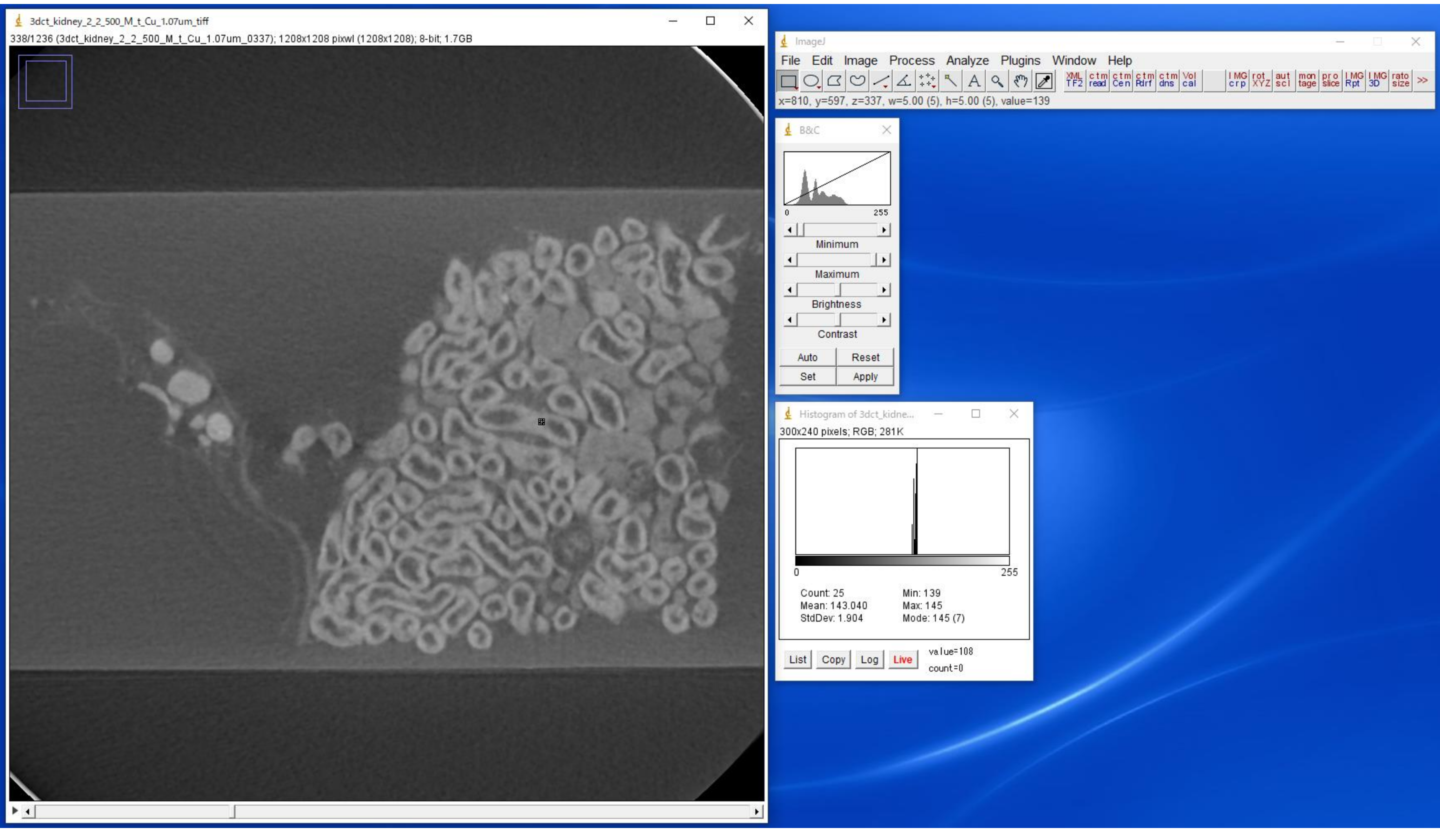

Supplementary Fig. 1. CNR measurement using the program *ImageJ* for CCD data.

Distal tubule measurement for 2_500_M_t nephron 2: air (top) and material (bottom) regions.

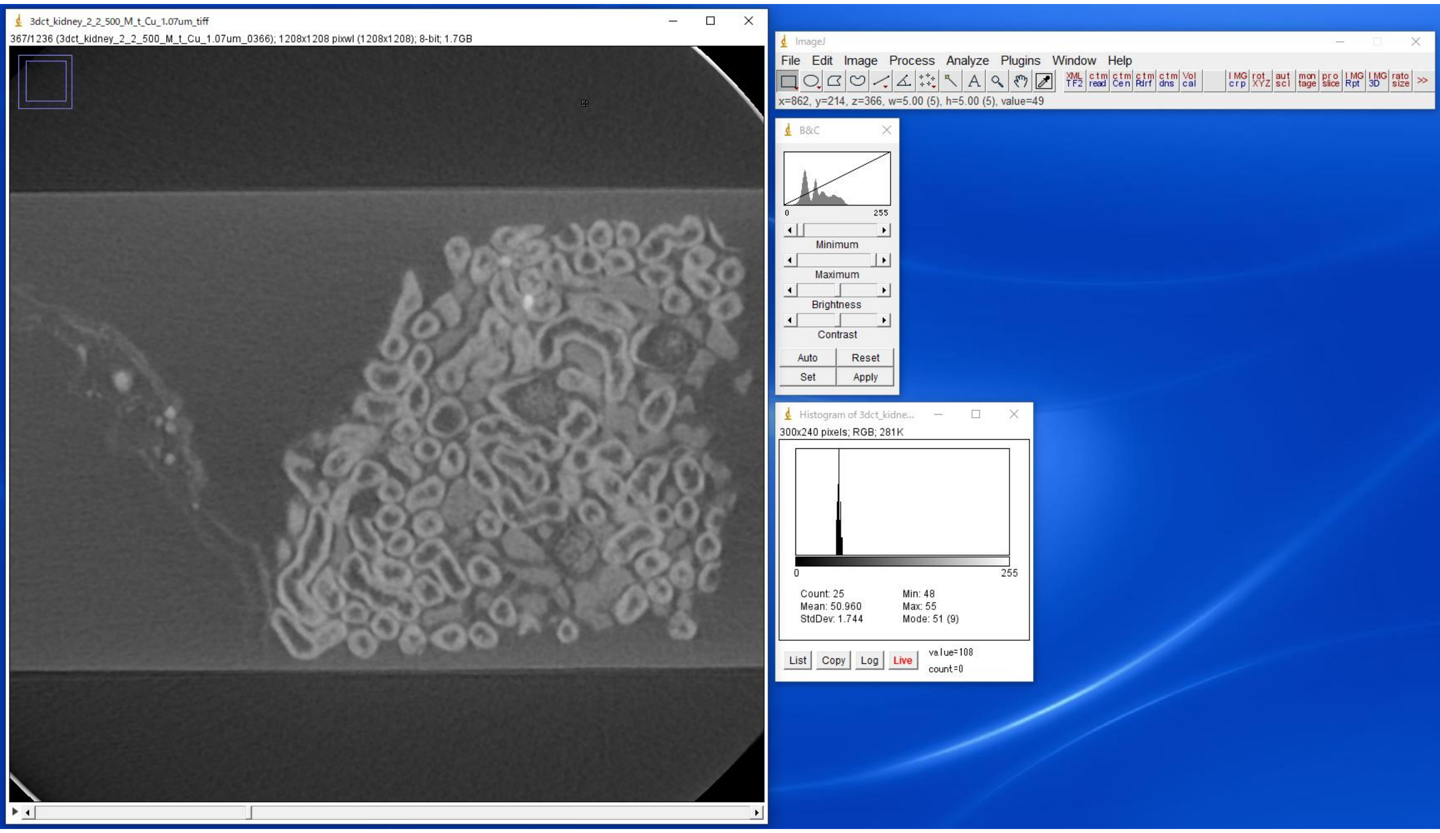

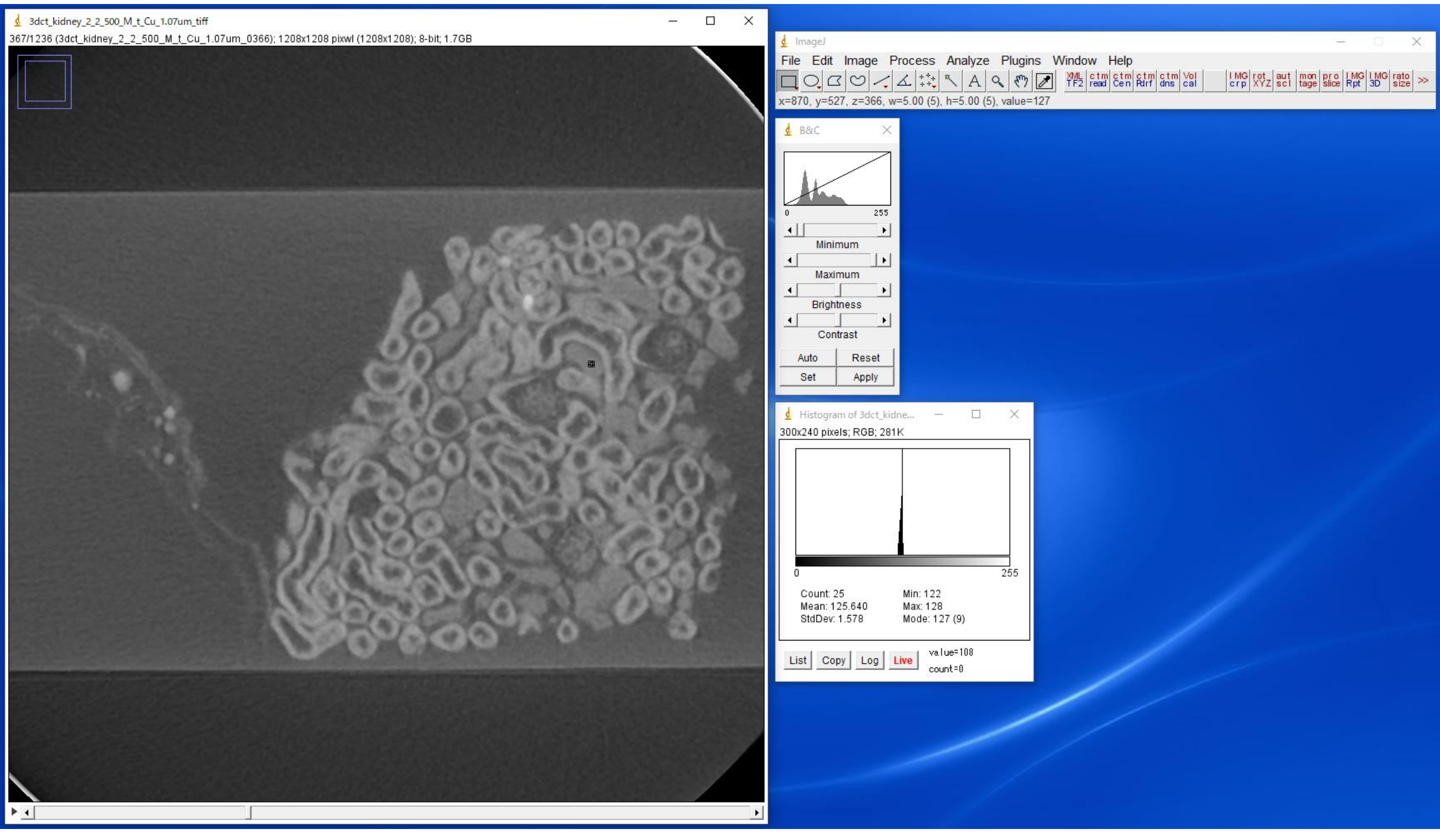

Supplementary Fig. 1. CNR measurement using the program *ImageJ* for CCD data.

Proximal tubule measurement for 2_500_M_t nephron 3: air (top) and material (bottom) regions.

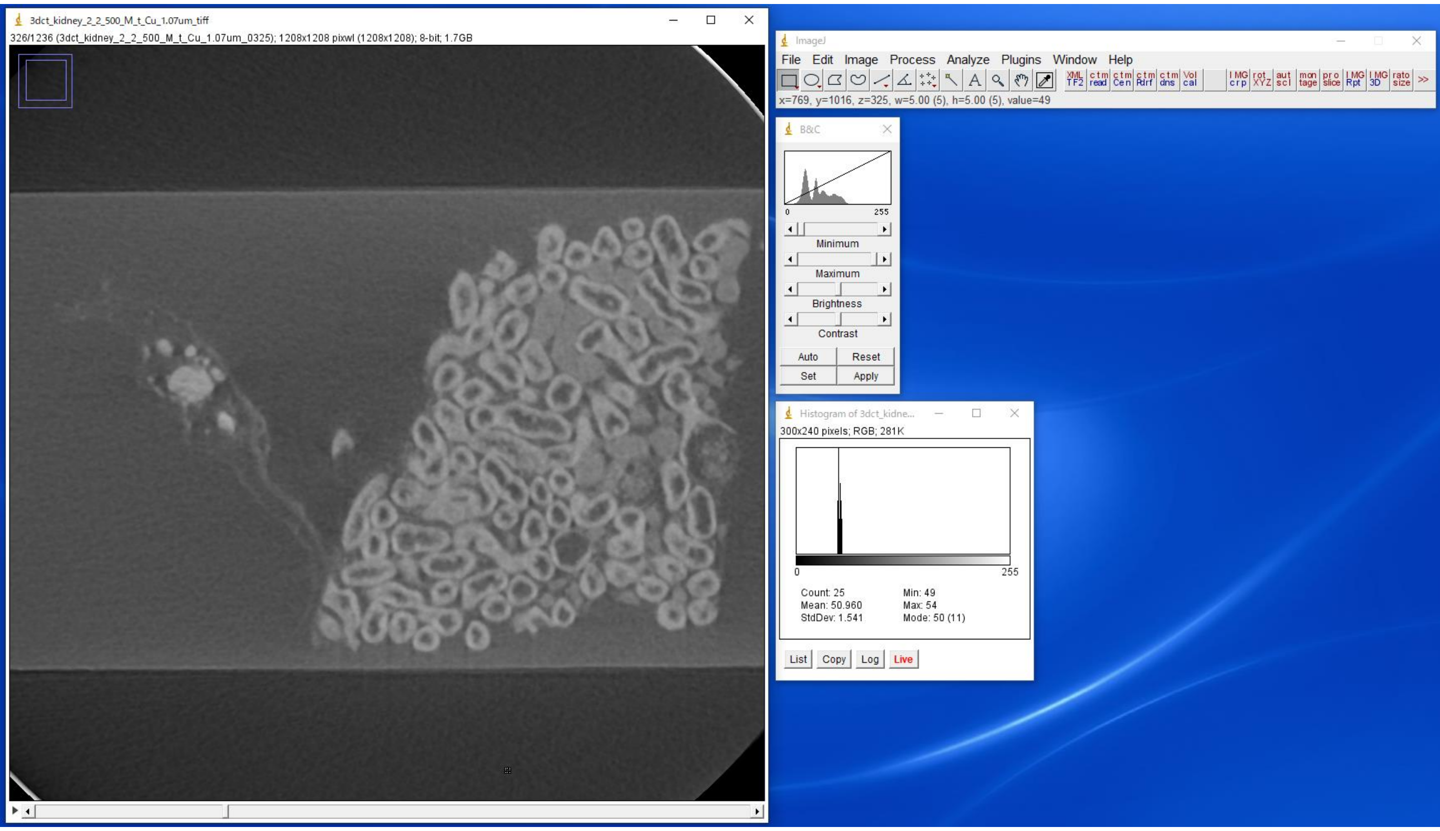

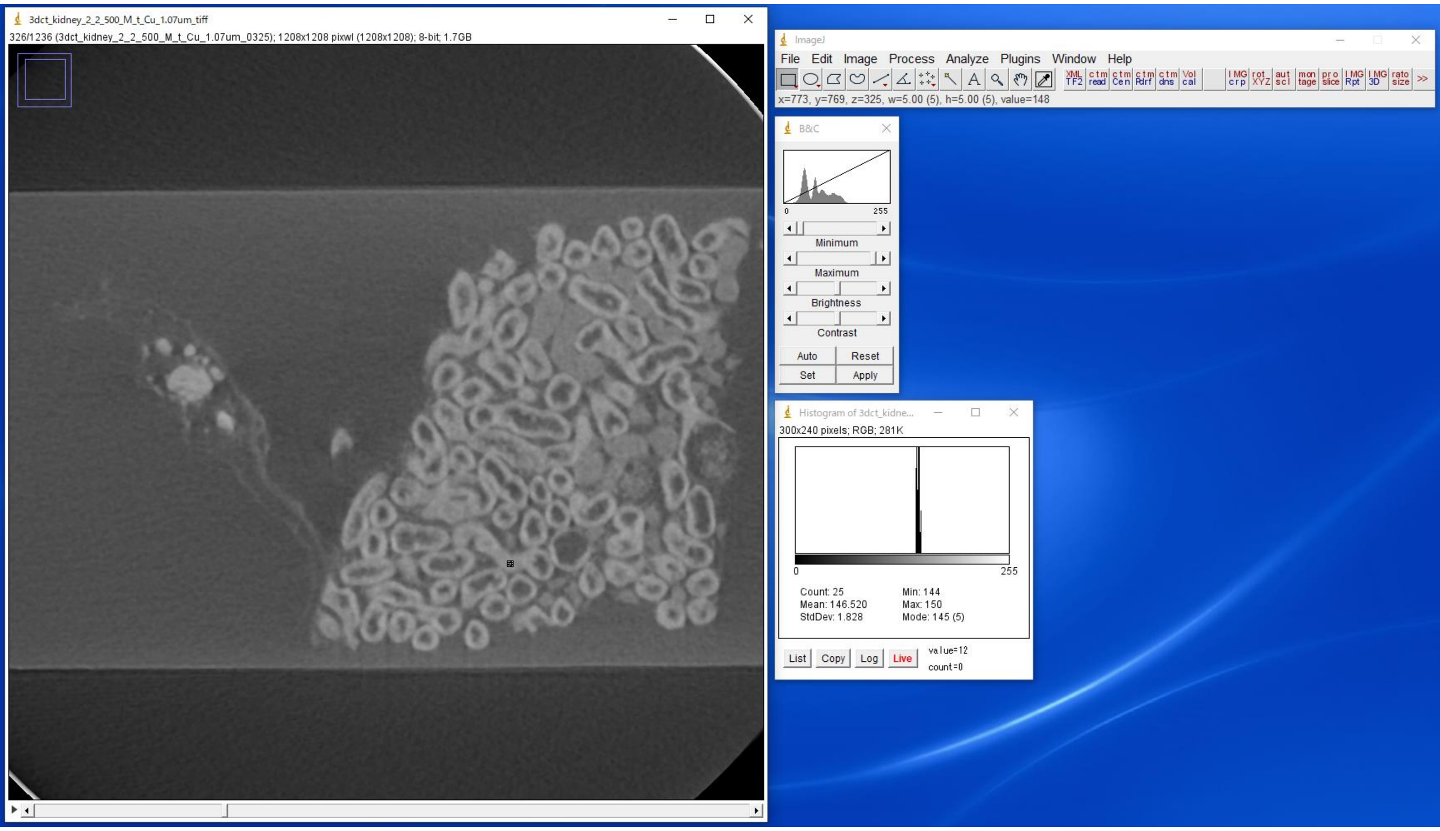

Supplementary Fig. 1. CNR measurement using the program *ImageJ* for CCD data.

Distal tubule measurement for 2_500_M_t nephron 3: air (top) and material (bottom) regions.

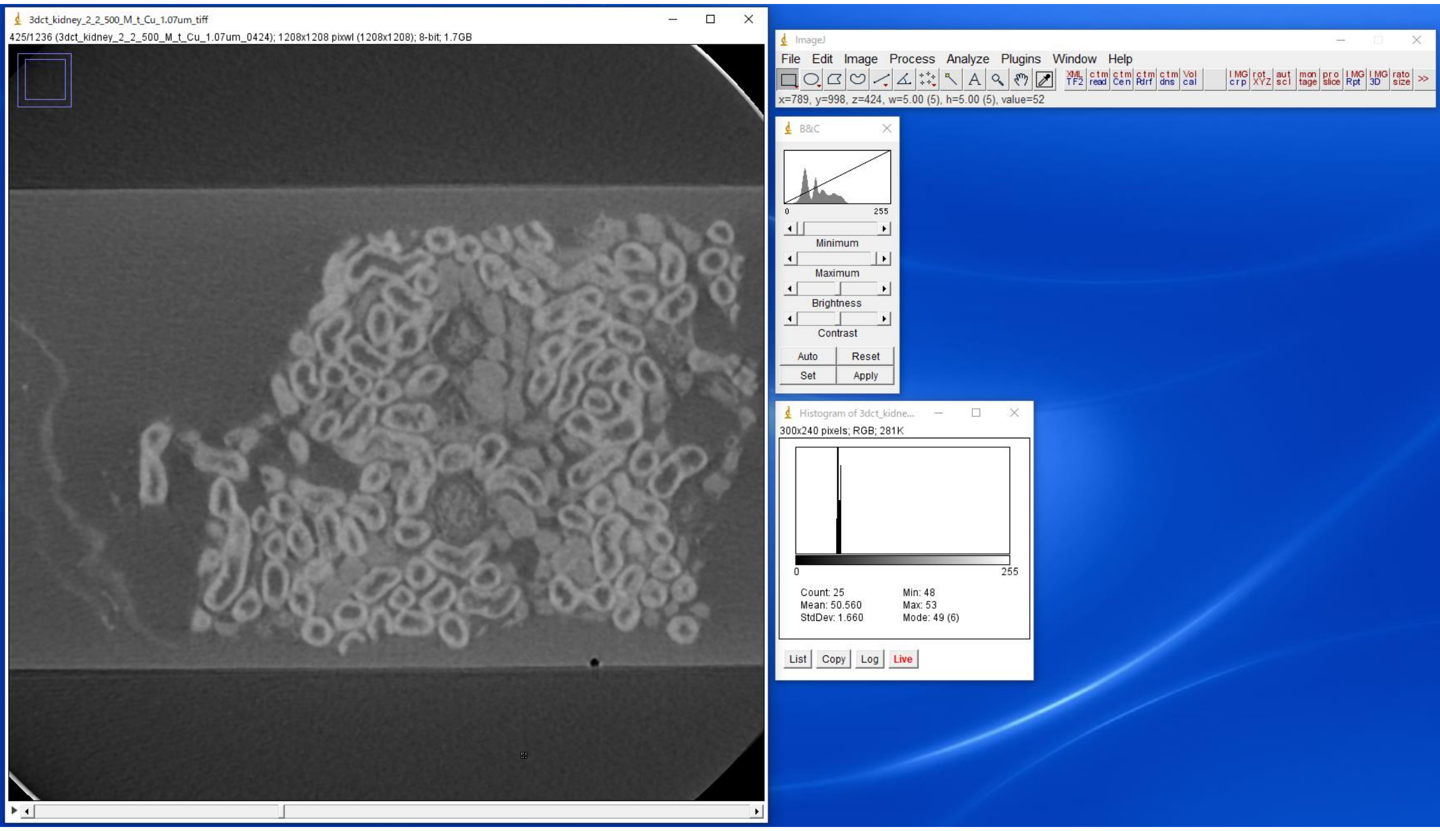

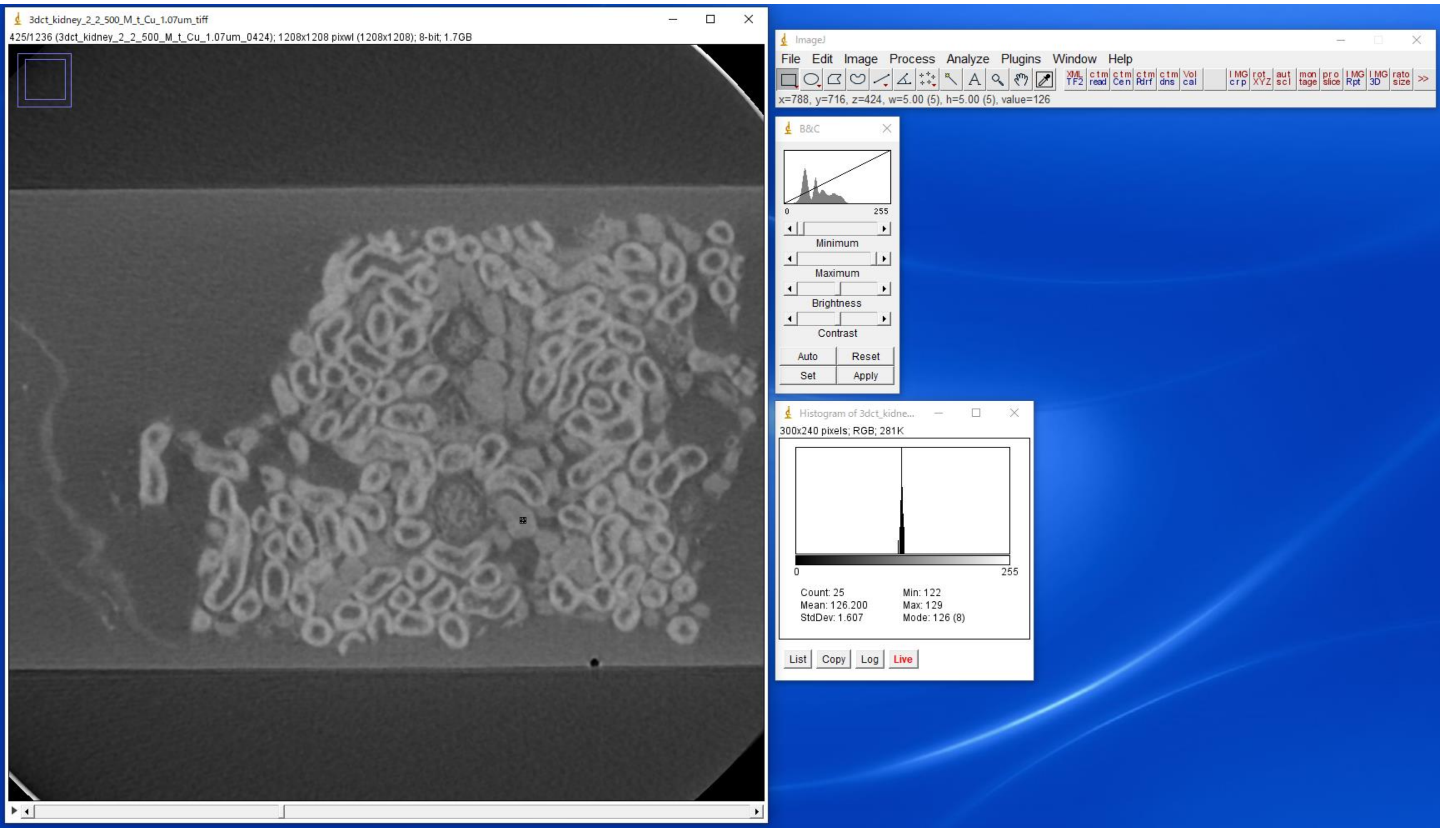

Supplementary Fig. 1. CNR measurement using the program *ImageJ* for CCD data.

Proximal tubule measurement for 2_500_M_t nephron 4: air (top) and material (bottom) regions.

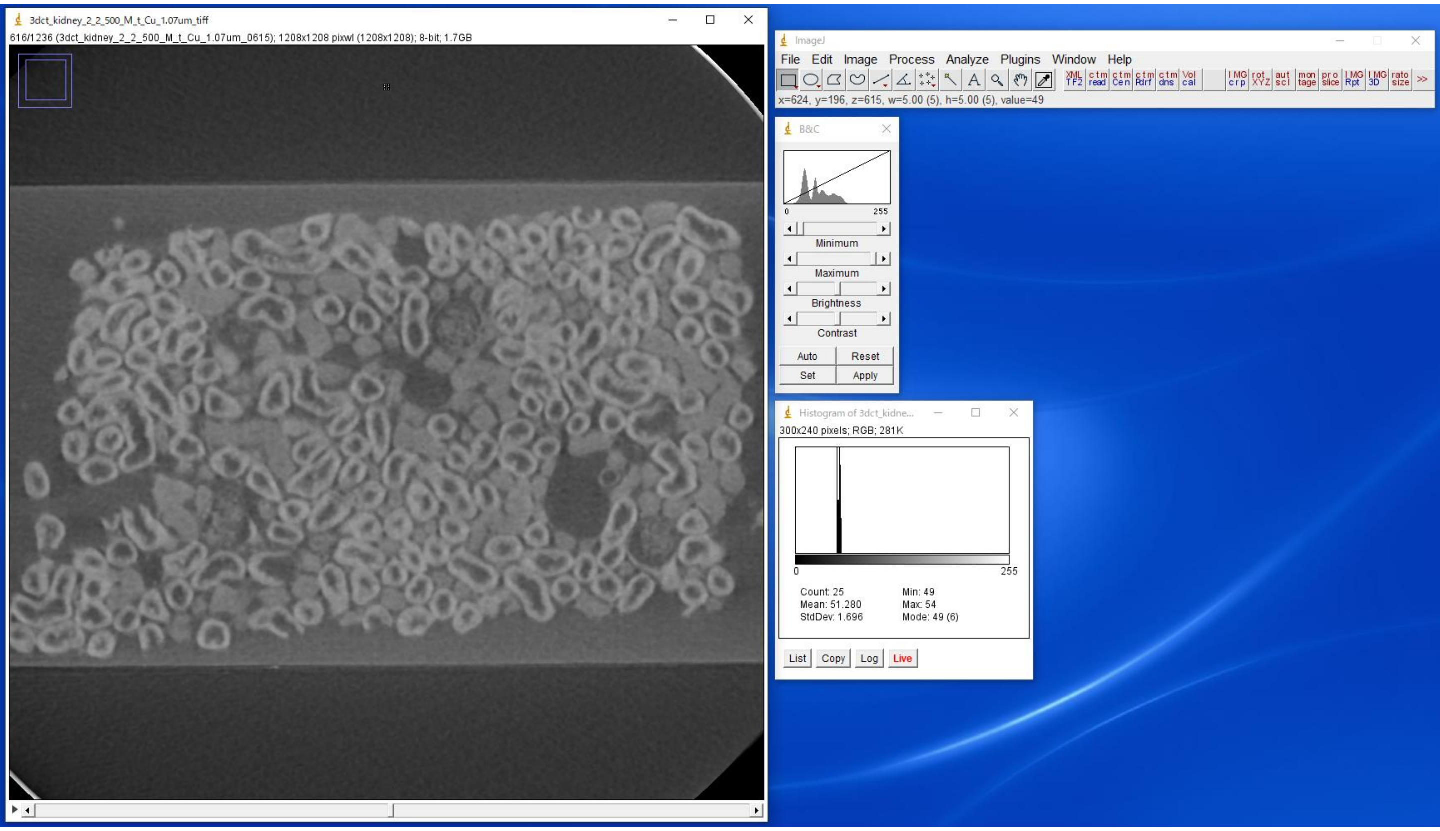

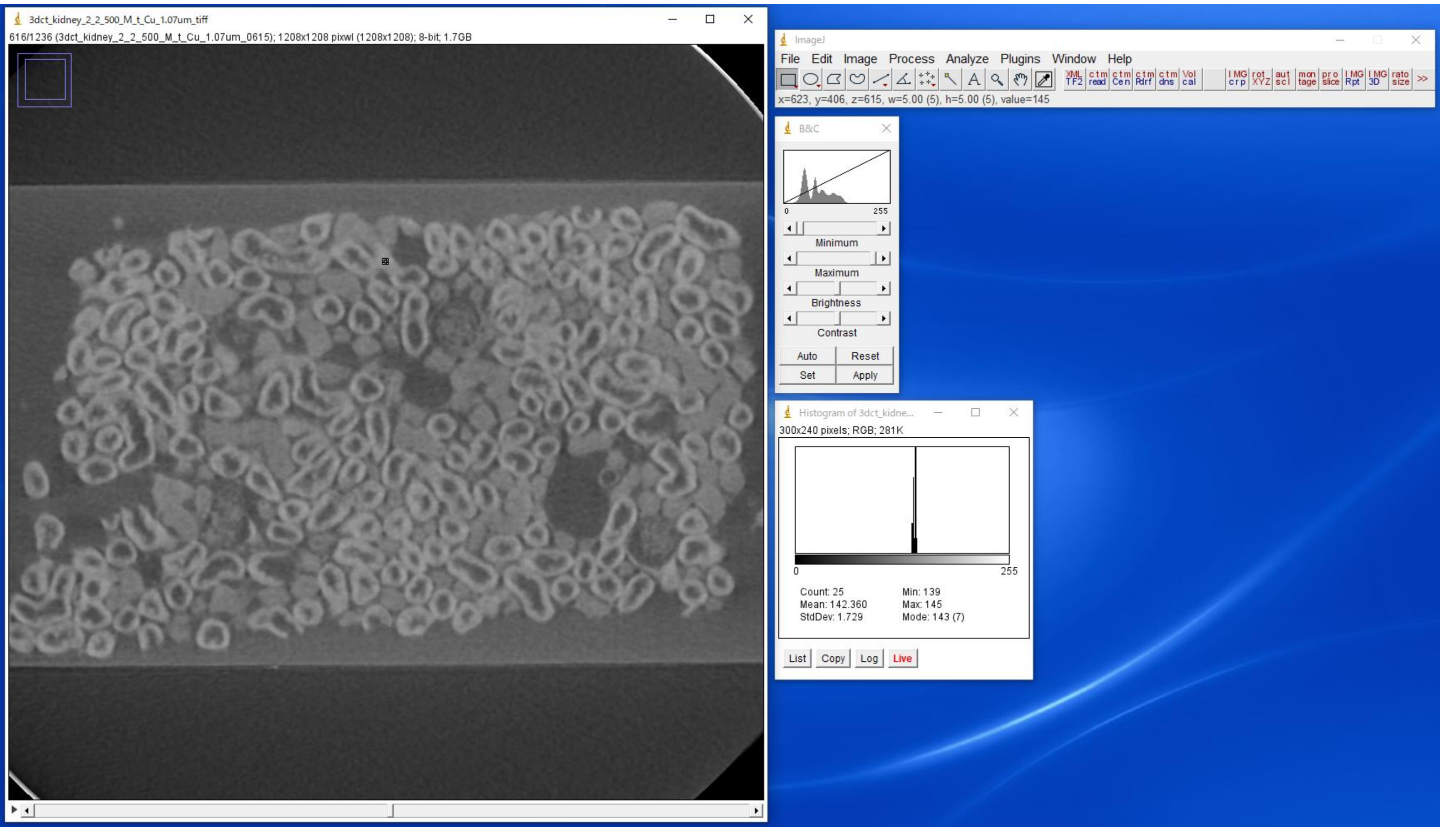

Supplementary Fig. 1. CNR measurement using the program *ImageJ* for CCD data.

Distal tubule measurement for 2_500_M_t nephron 4: air (top) and material (bottom) regions.

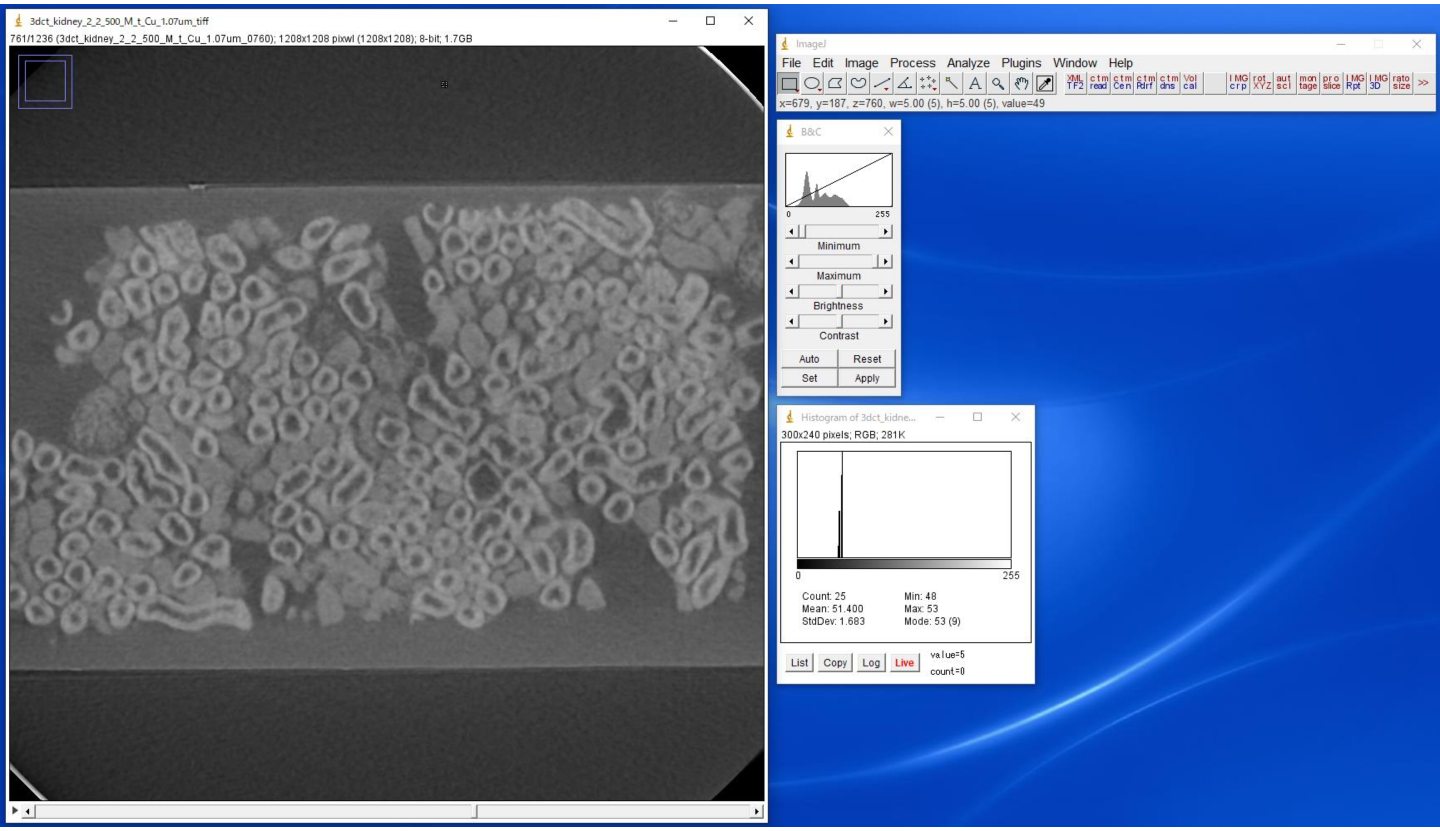

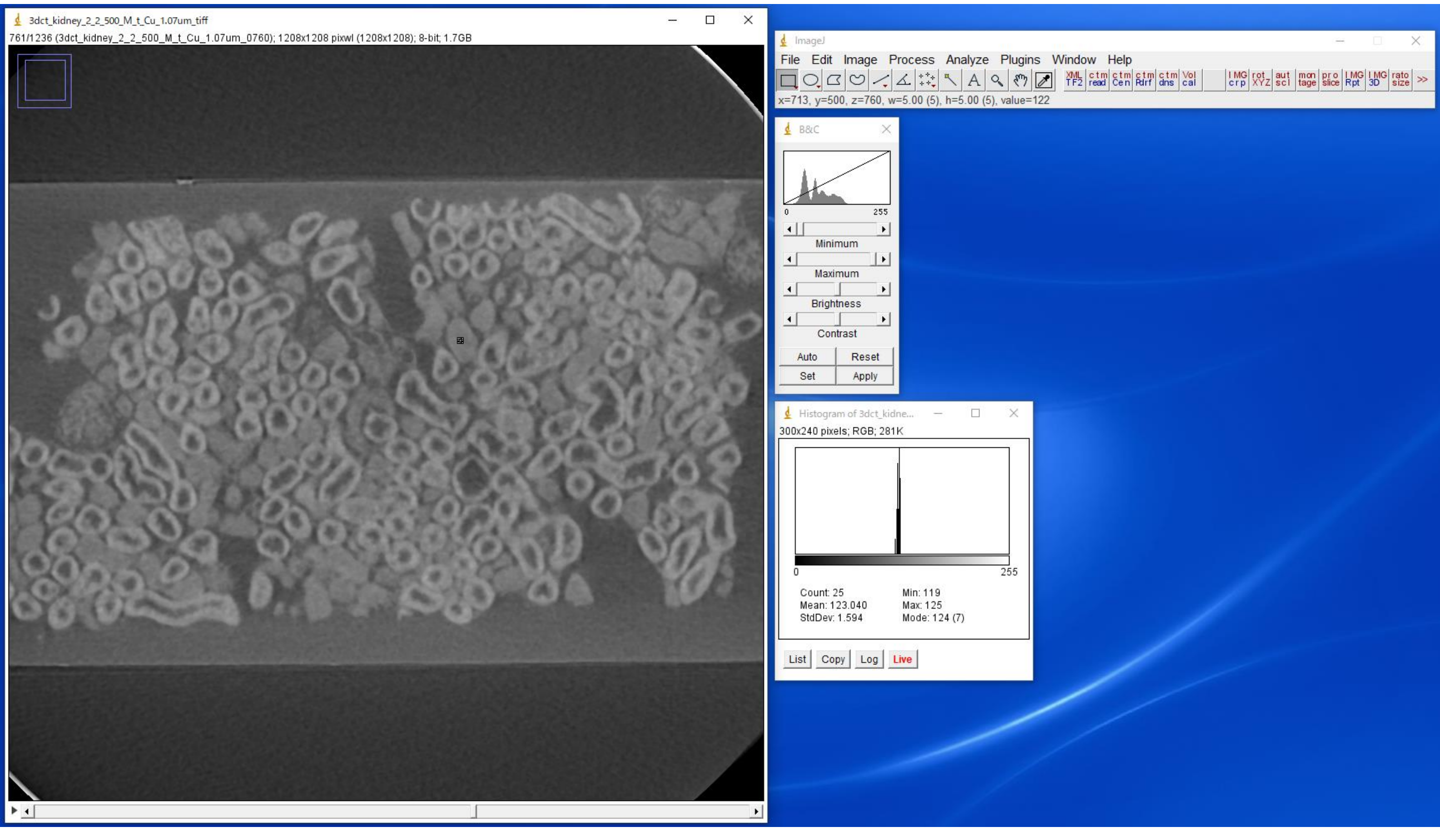

Supplementary Fig. 1. CNR measurement using the program *ImageJ* for CCD data.

Proximal tubule measurement for 2_500_M_t nephron 5: air (top) and material (bottom) regions.

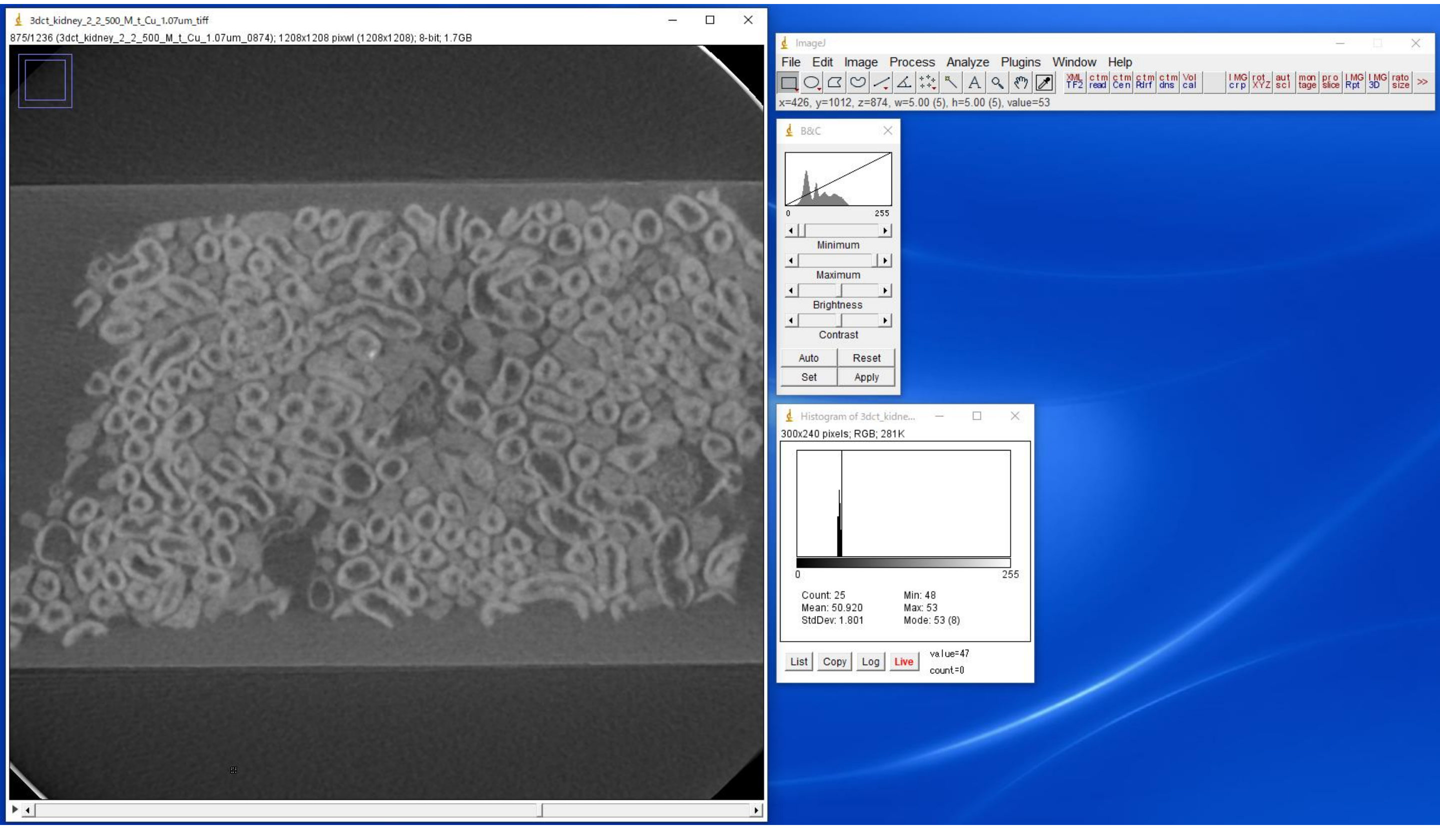

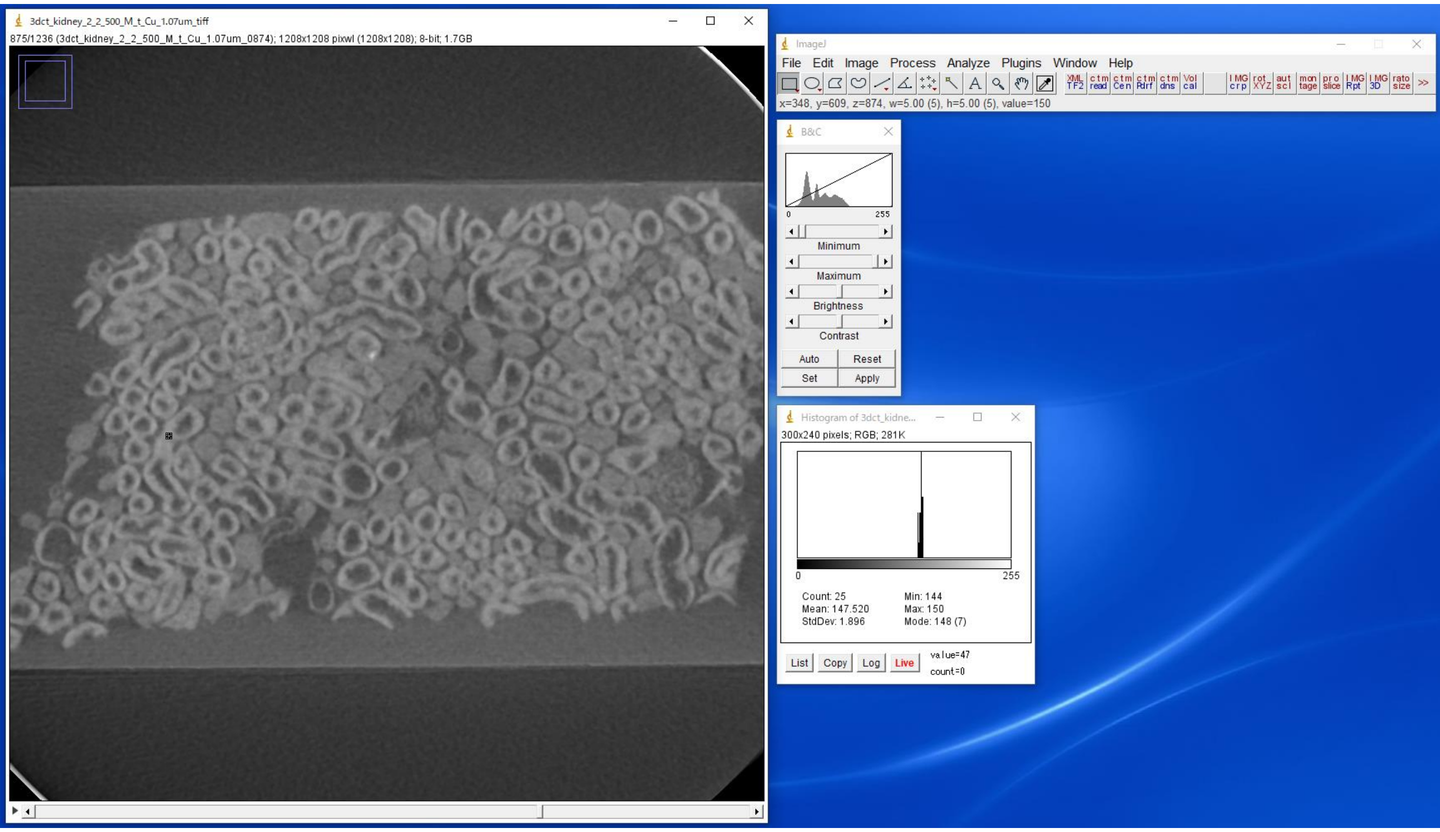

Supplementary Fig. 1. CNR measurement using the program *ImageJ* for CCD data.

Distal tubule measurement for 2_500_M_t nephron 5: air (top) and material (bottom) regions.

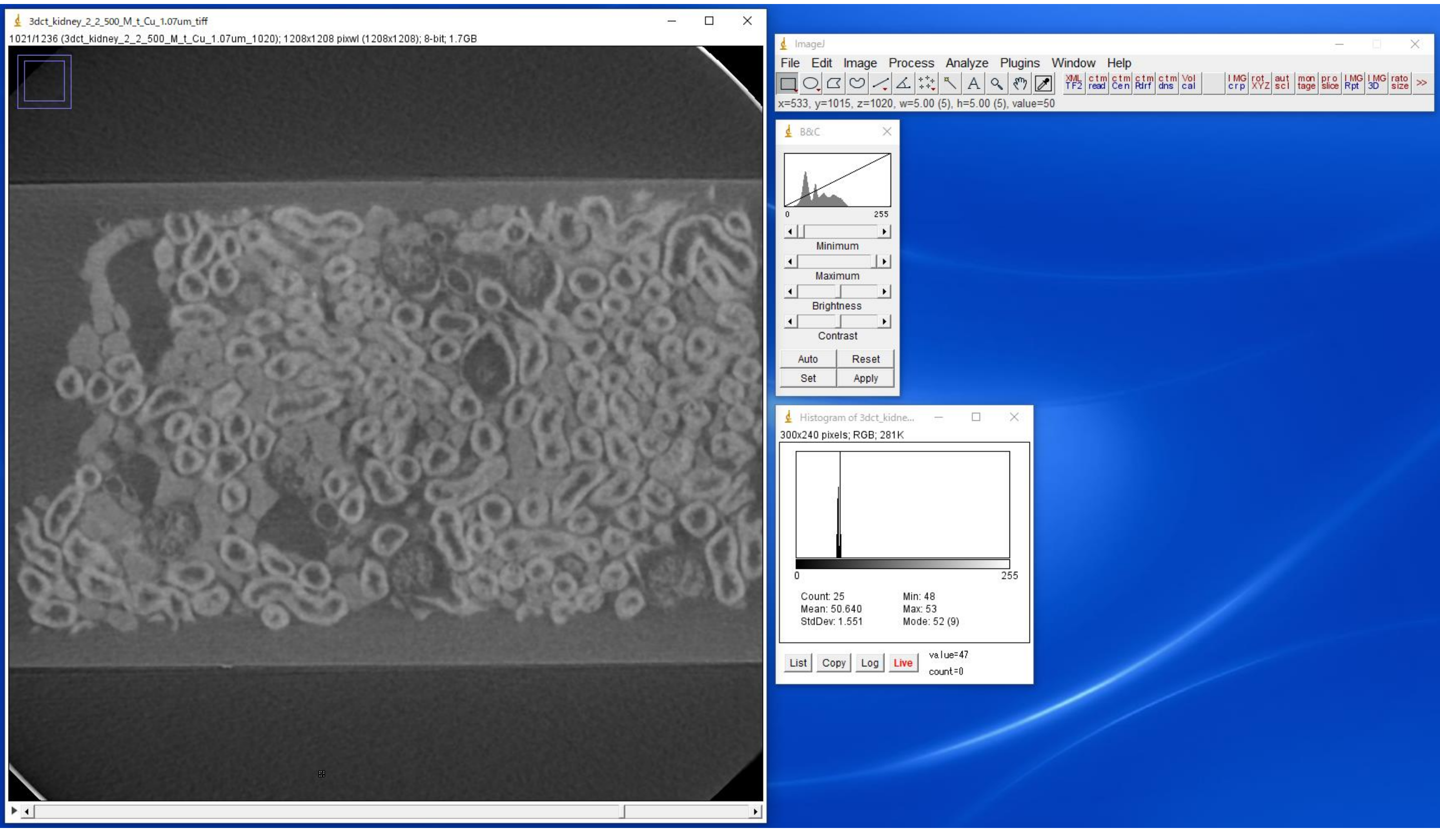

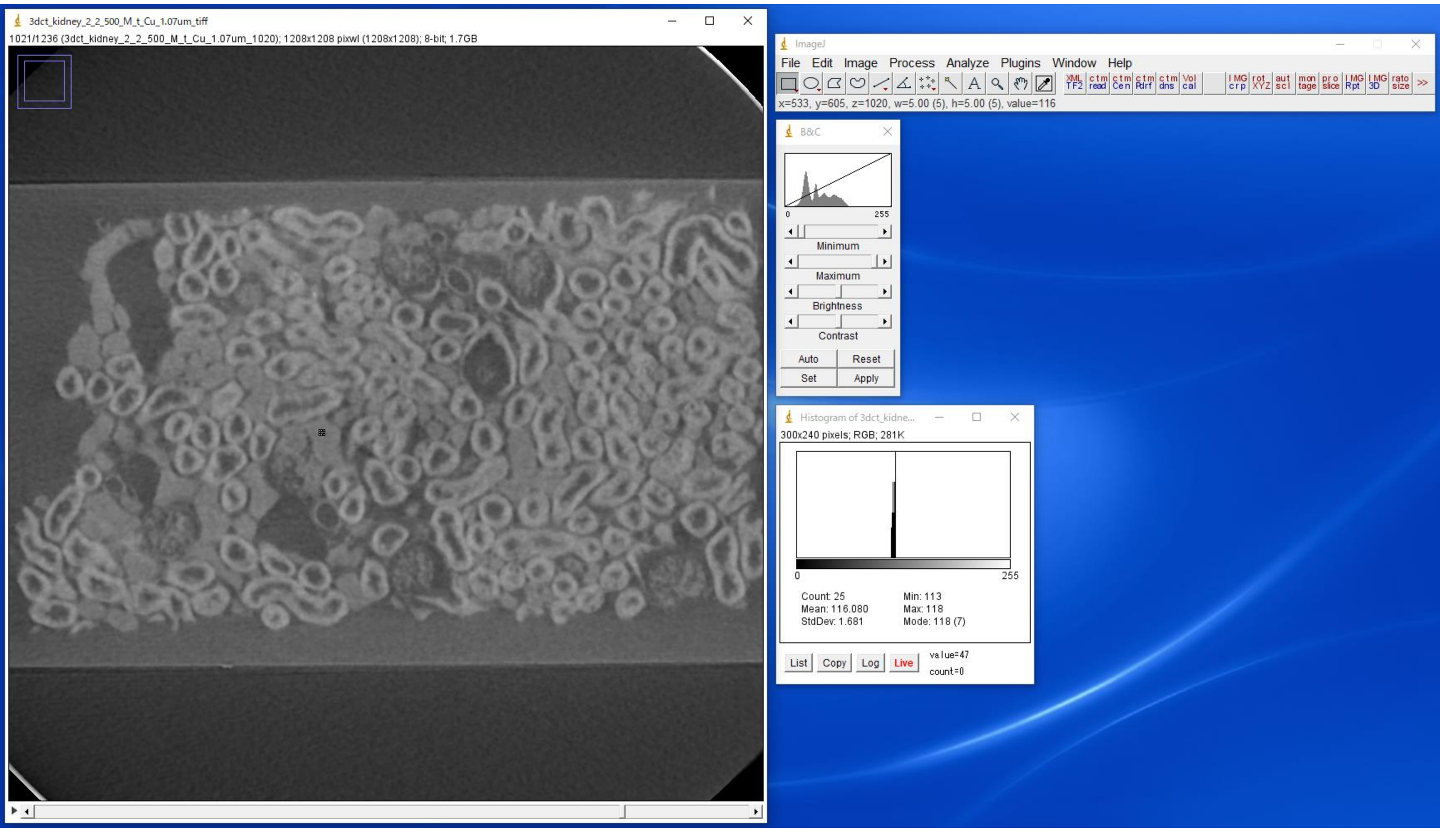

Supplementary Fig. 1. CNR measurement using the program *ImageJ* for CCD data.

Proximal tubule measurement for 1_300_H_t nephron 1: air (top) and material (bottom) regions.

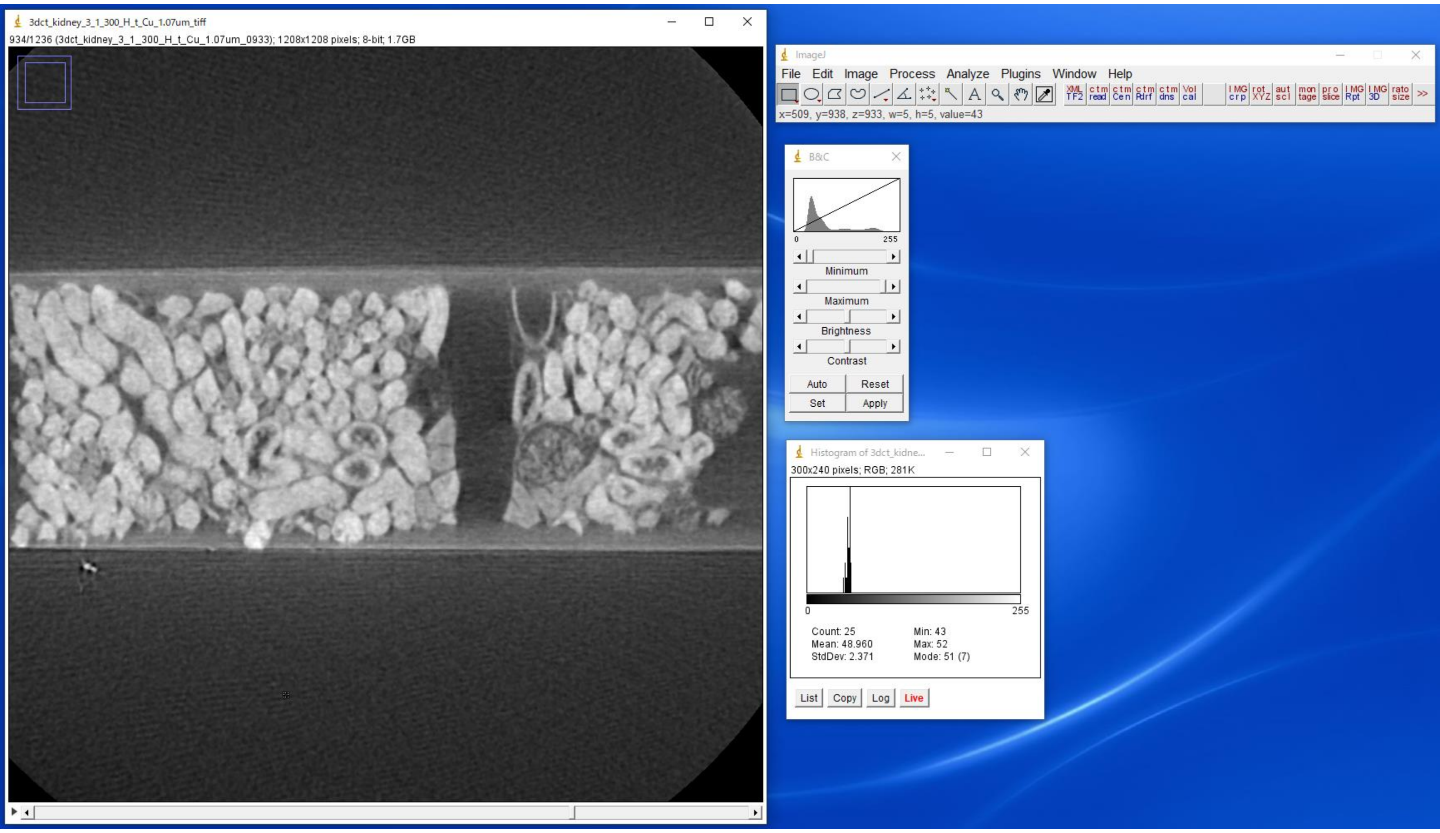

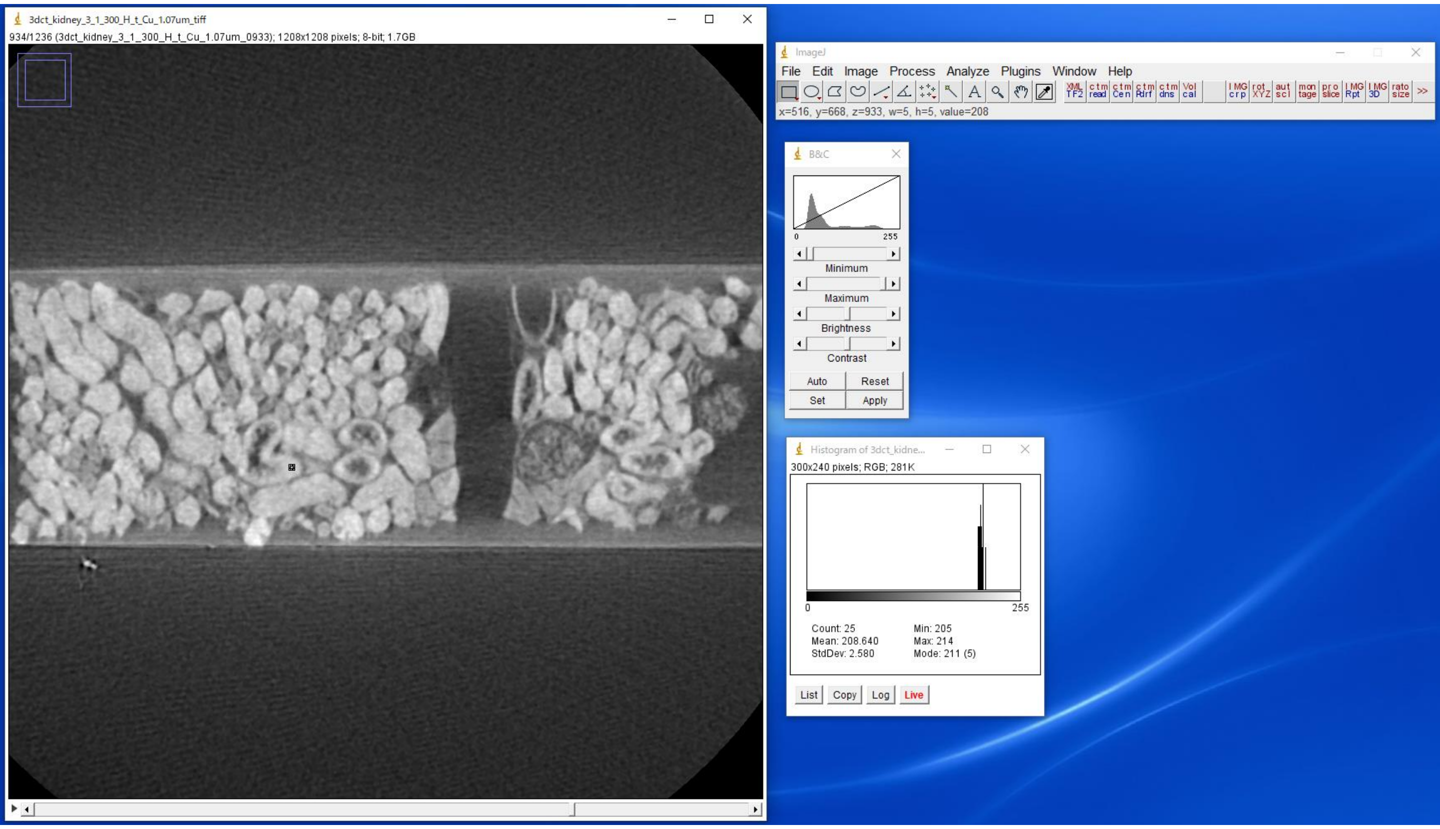

Supplementary Fig. 1. CNR measurement using the program *ImageJ* for CCD data.

Distal tubule measurement for 1_300_H_t nephron 1: air (top) and material (bottom) regions.

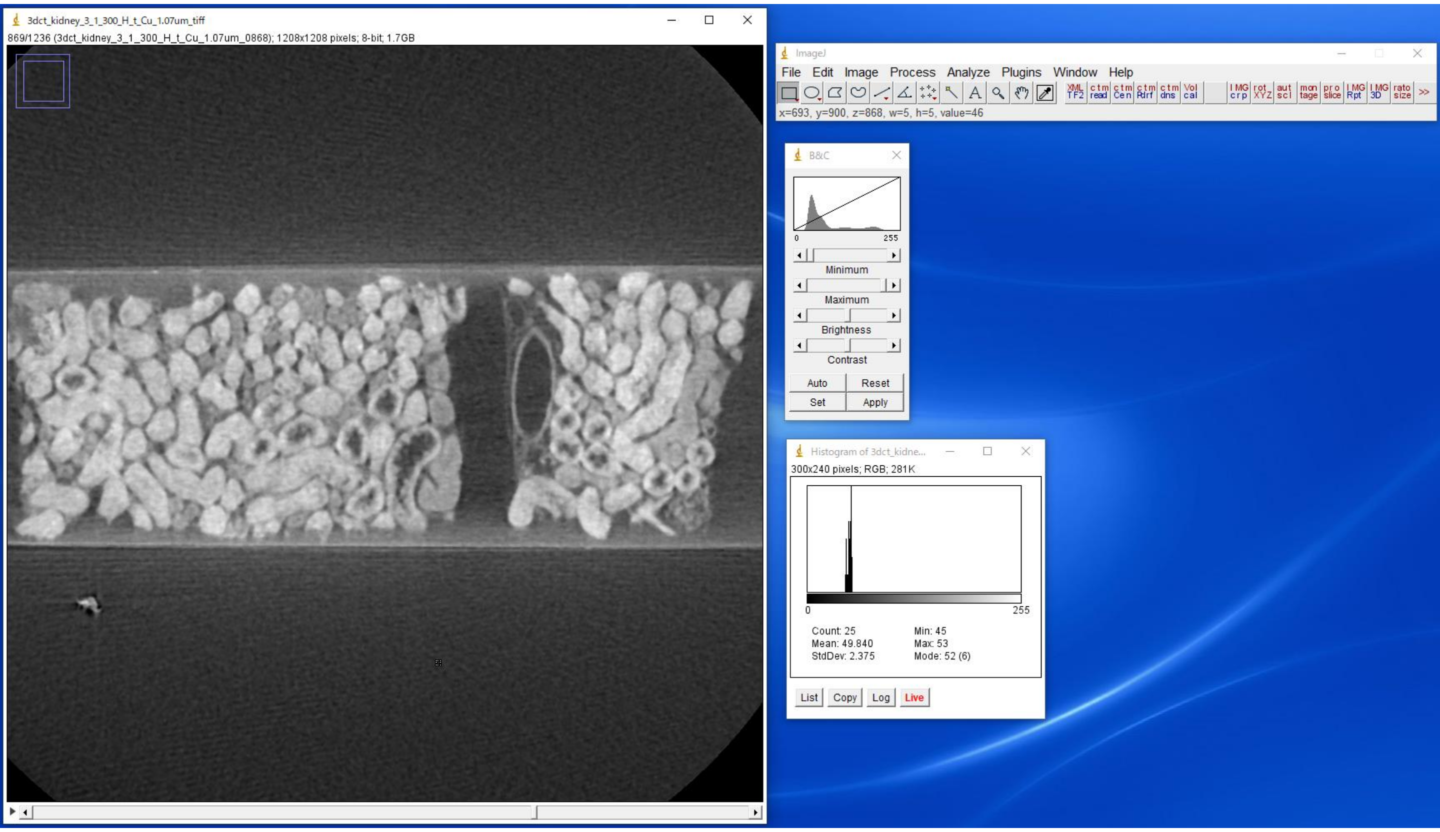

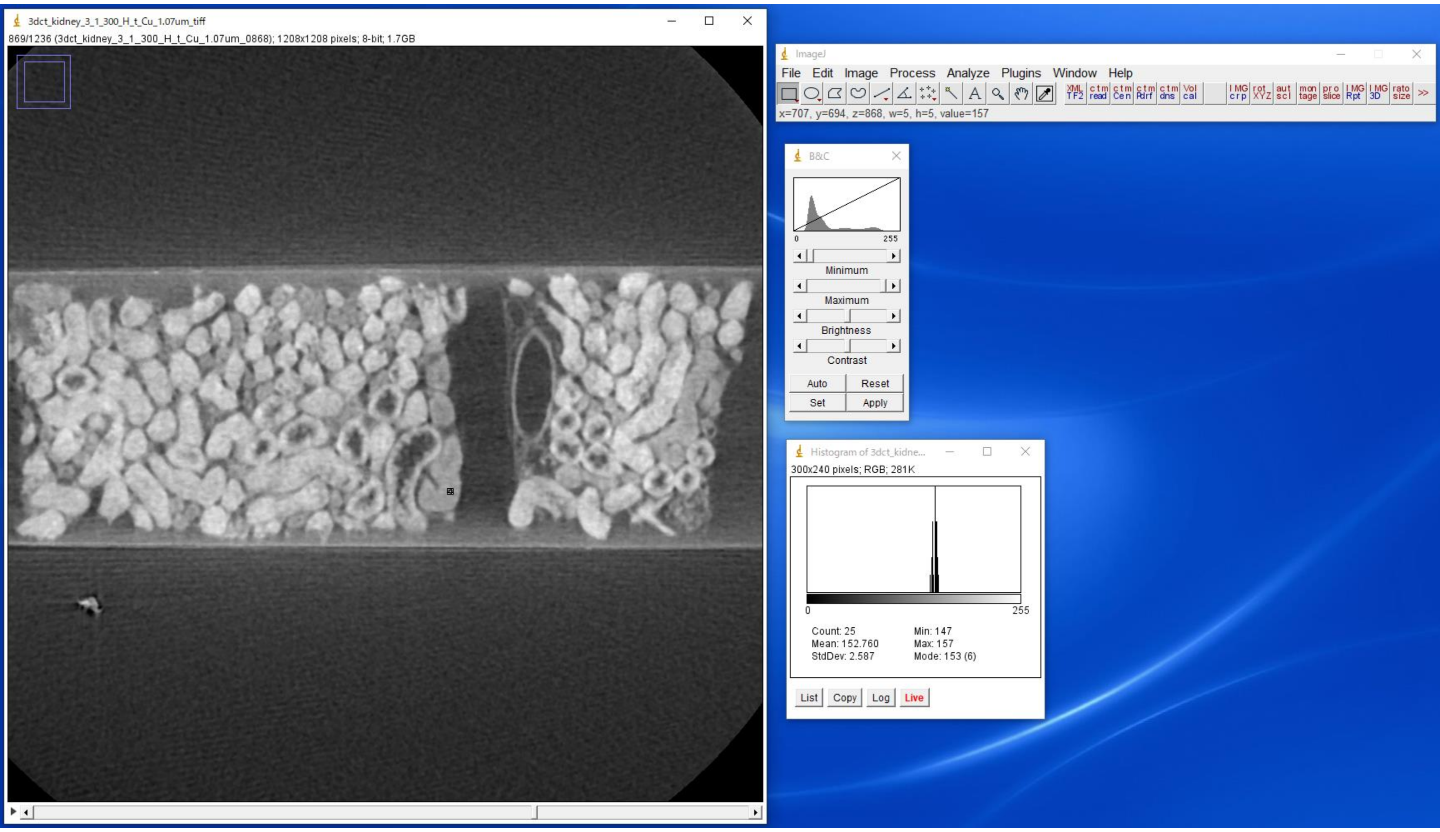

Supplementary Fig. 1. CNR measurement using the program *ImageJ* for CCD data.

Proximal tubule measurement for 1_300_H_t nephron 2: air (top) and material (bottom) regions.

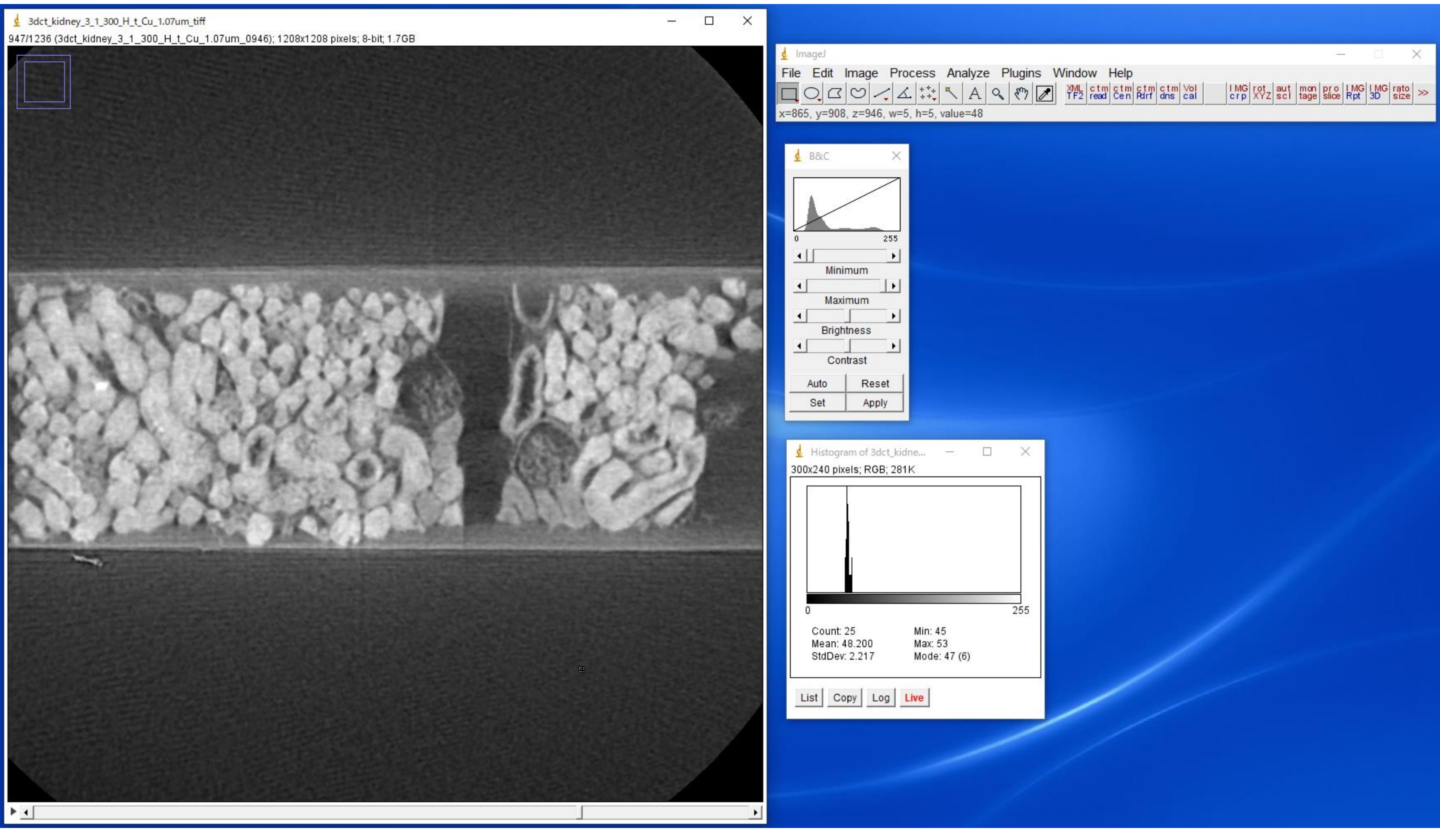

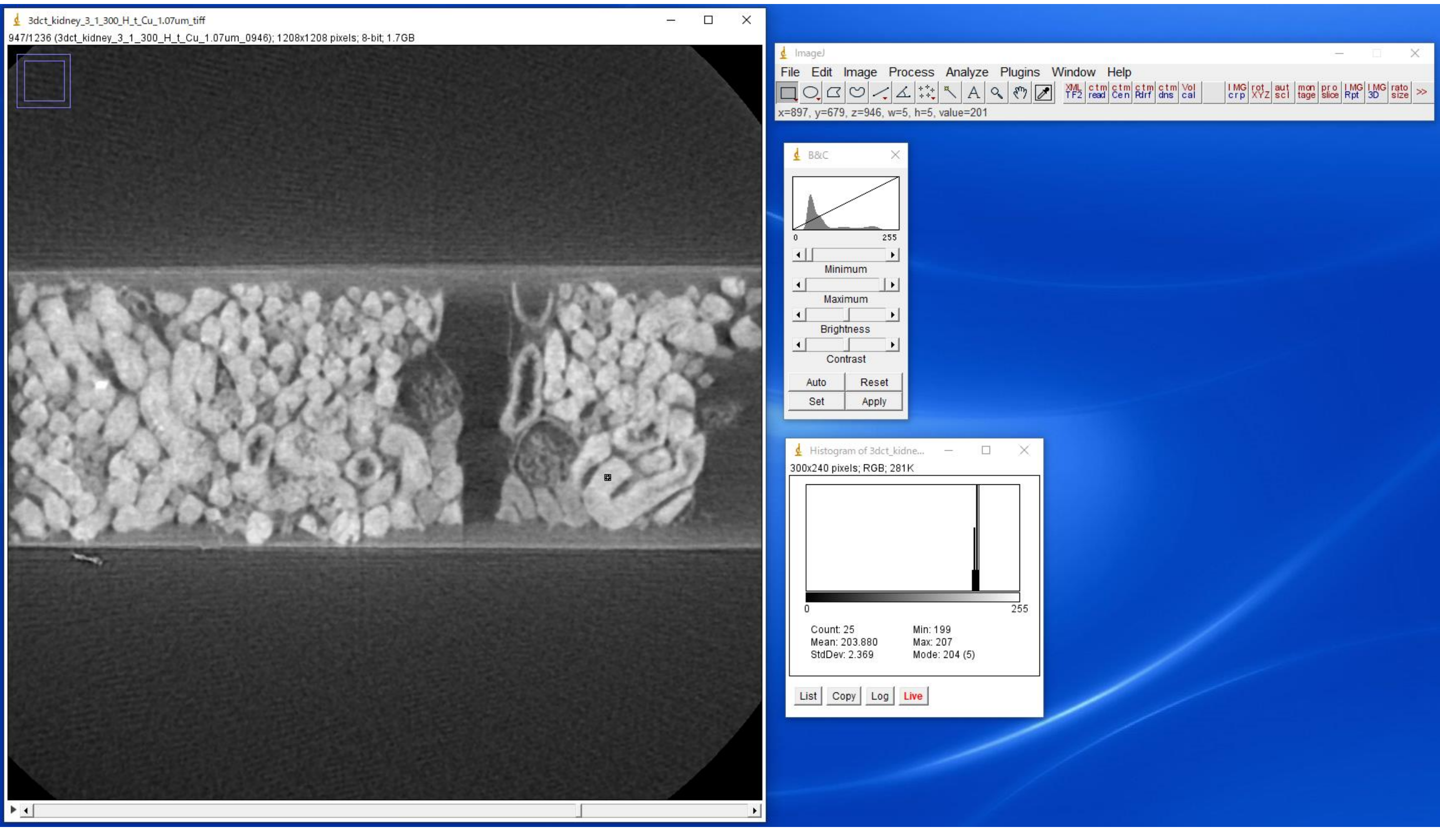

Supplementary Fig. 1. CNR measurement using the program *ImageJ* for CCD data.

Distal tubule measurement for 1_300_H_t nephron 2: air (top) and material (bottom) regions.

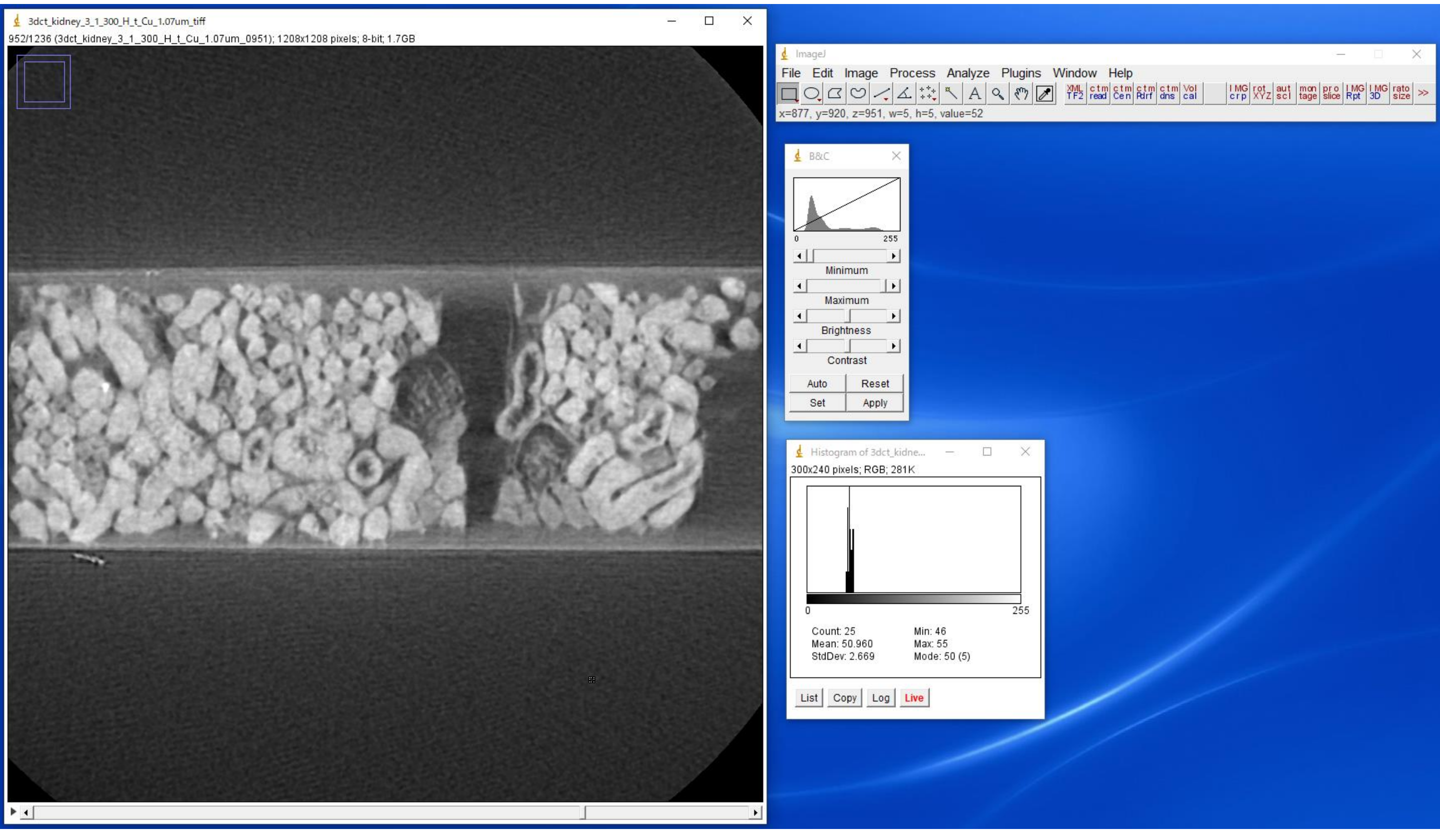

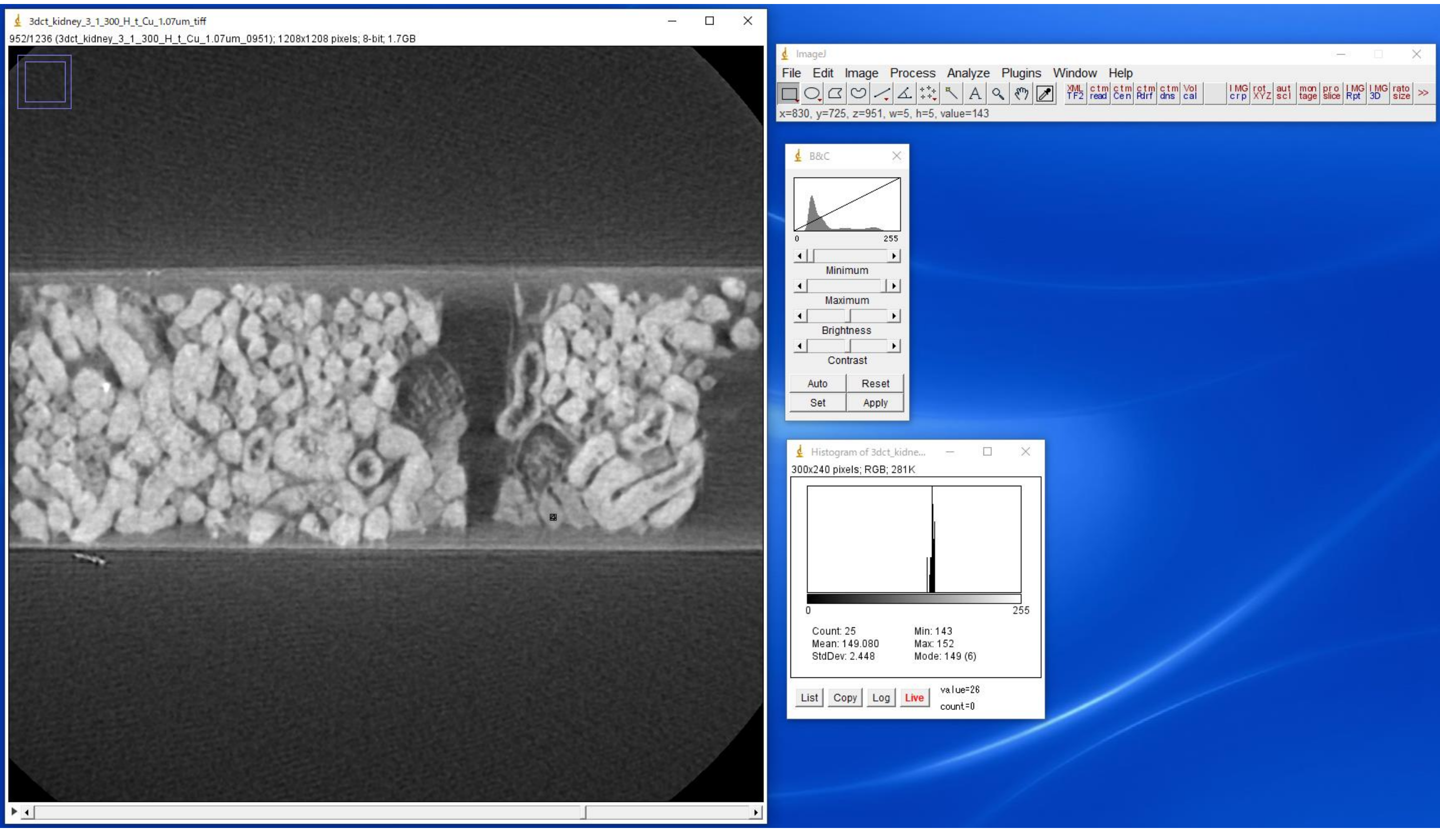

Supplementary Fig. 1. CNR measurement using the program *ImageJ* for CCD data.

Proximal tubule measurement for 1_300_H_t nephron 3: air (top) and material (bottom) regions.

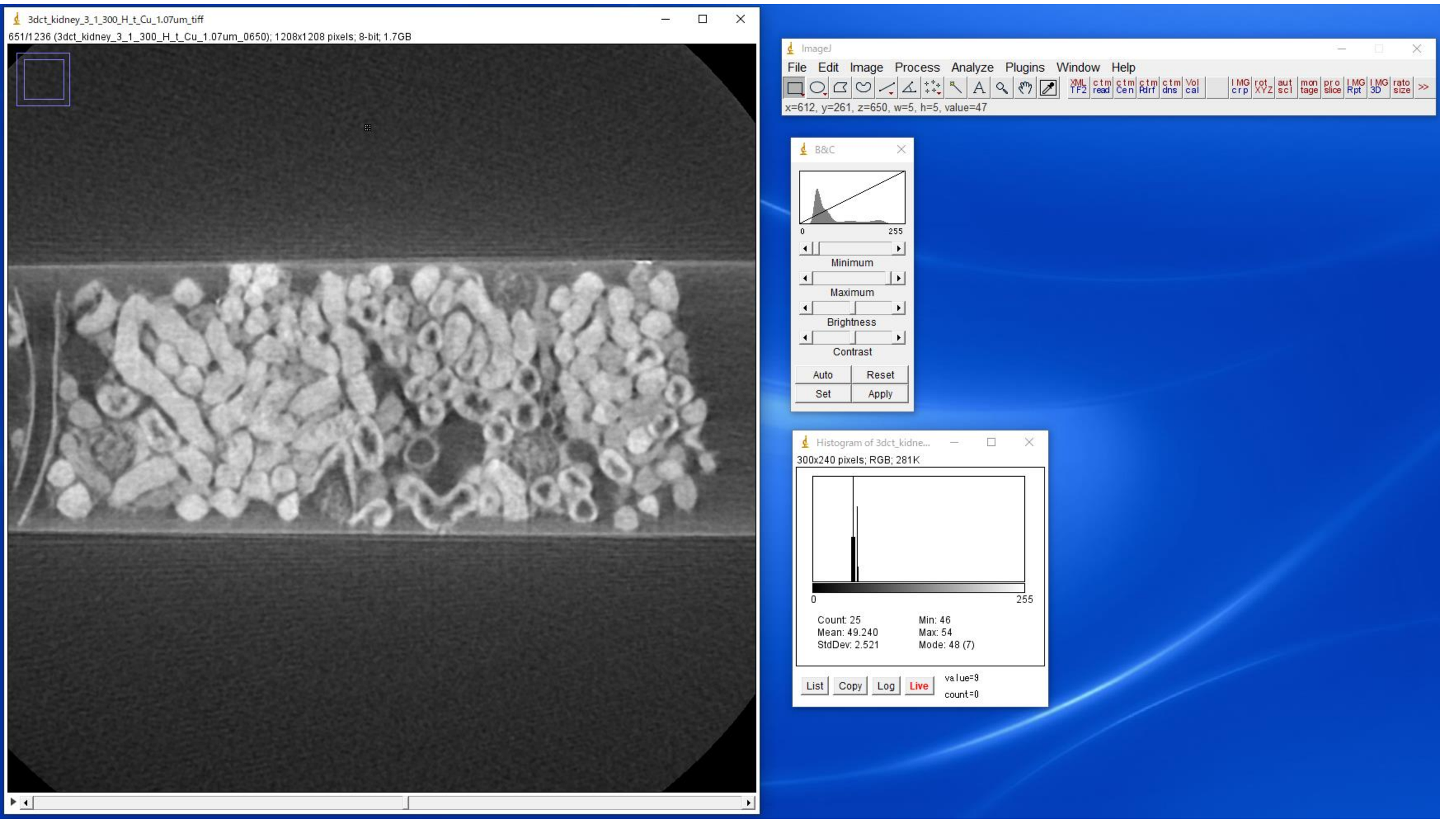

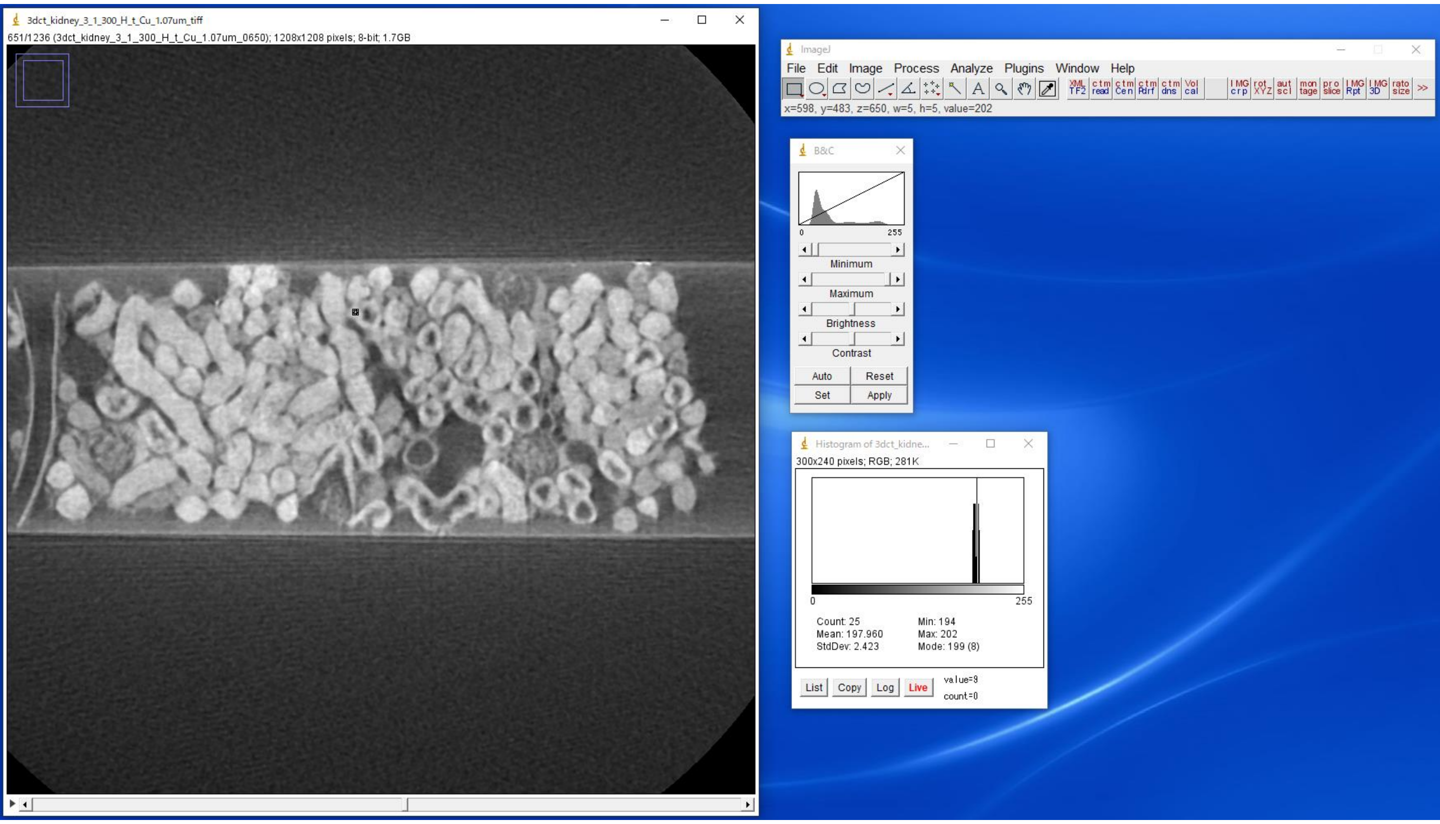

Supplementary Fig. 1. CNR measurement using the program *ImageJ* for CCD data.

Distal tubule measurement for 1_300_H_t nephron 3: air (top) and material (bottom) regions.

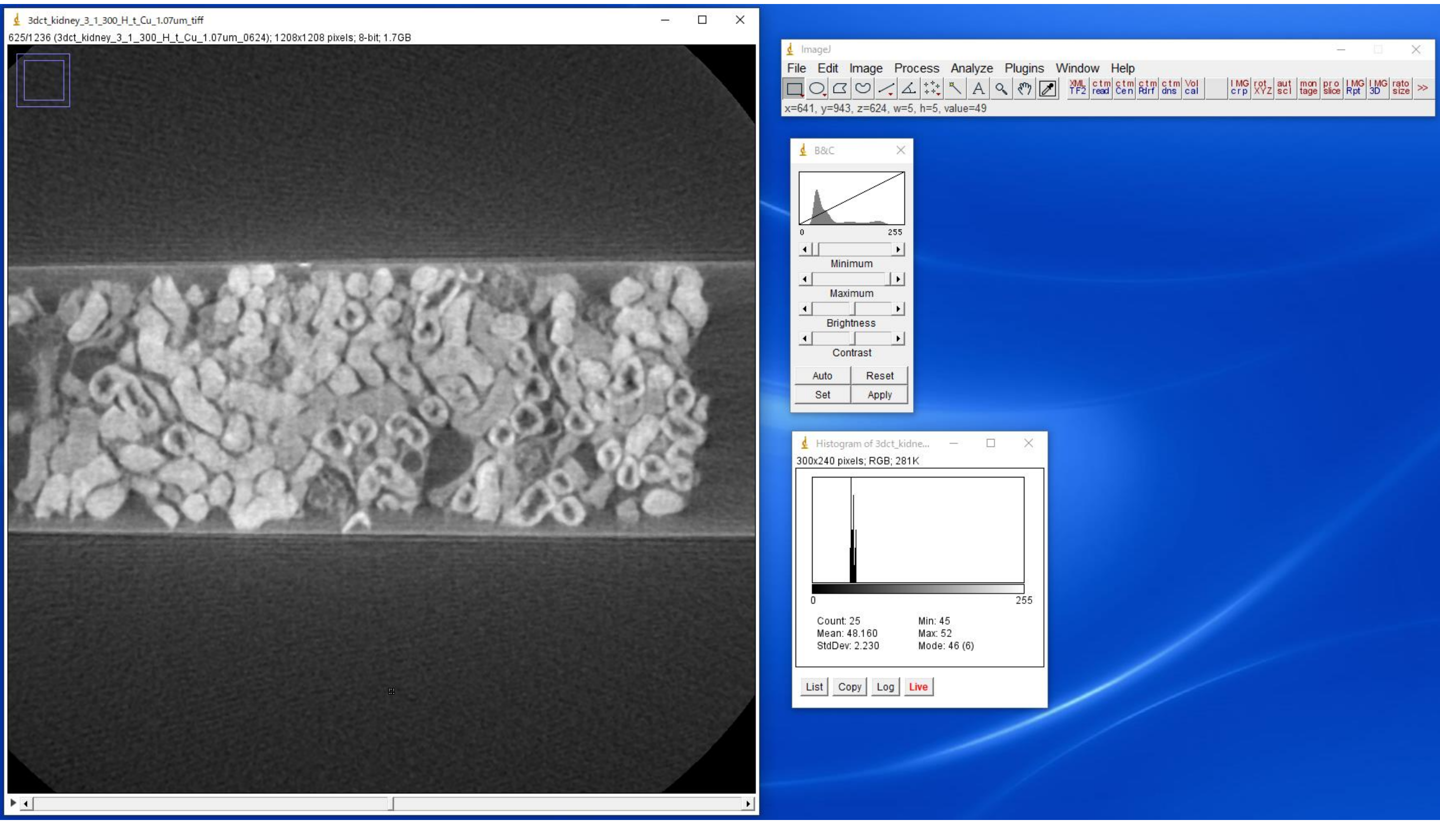

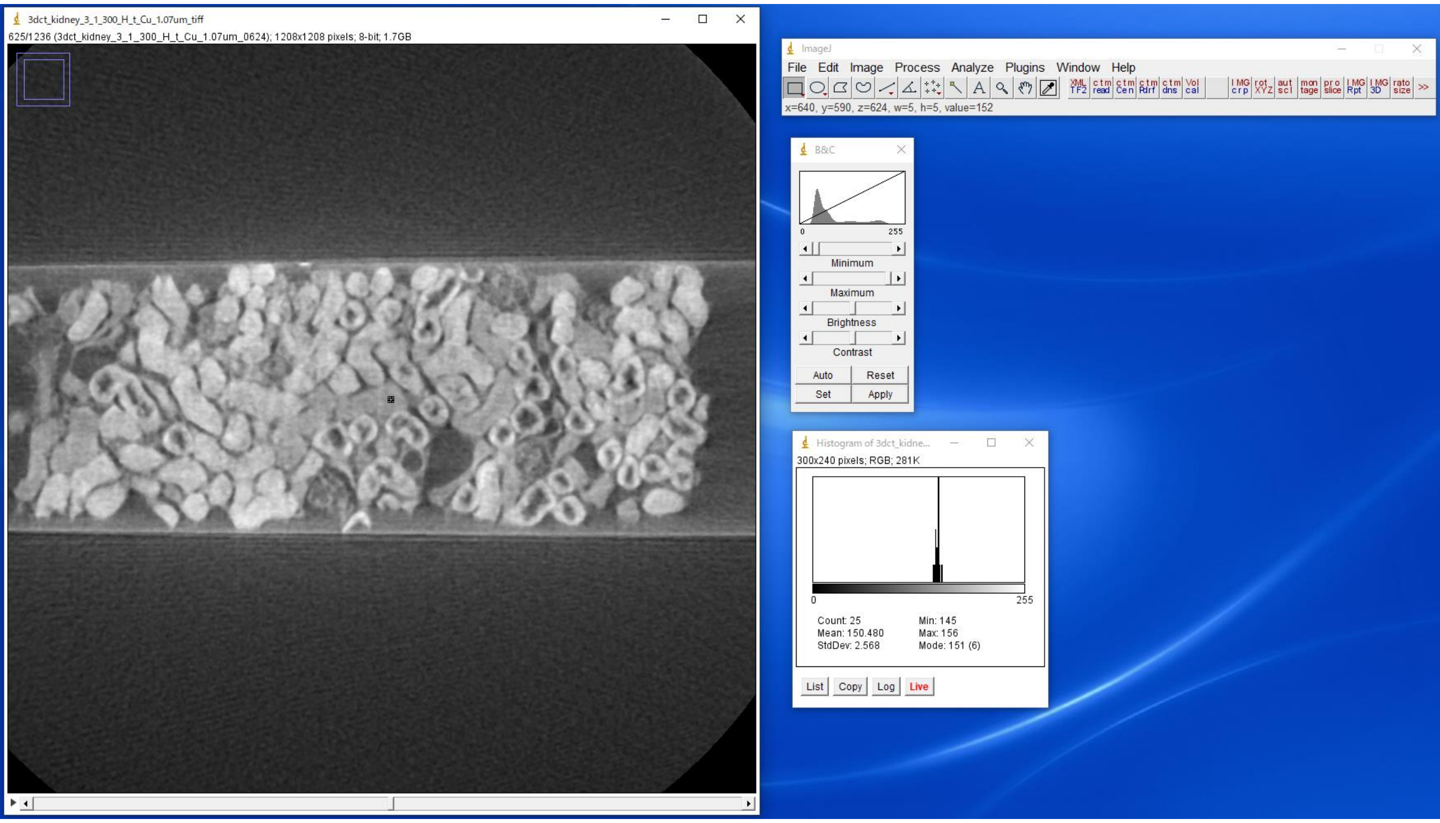

Supplementary Fig. 1. CNR measurement using the program *ImageJ* for CCD data.

Proximal tubule measurement for 1_300_H_t nephron 4: air (top) and material (bottom) regions.

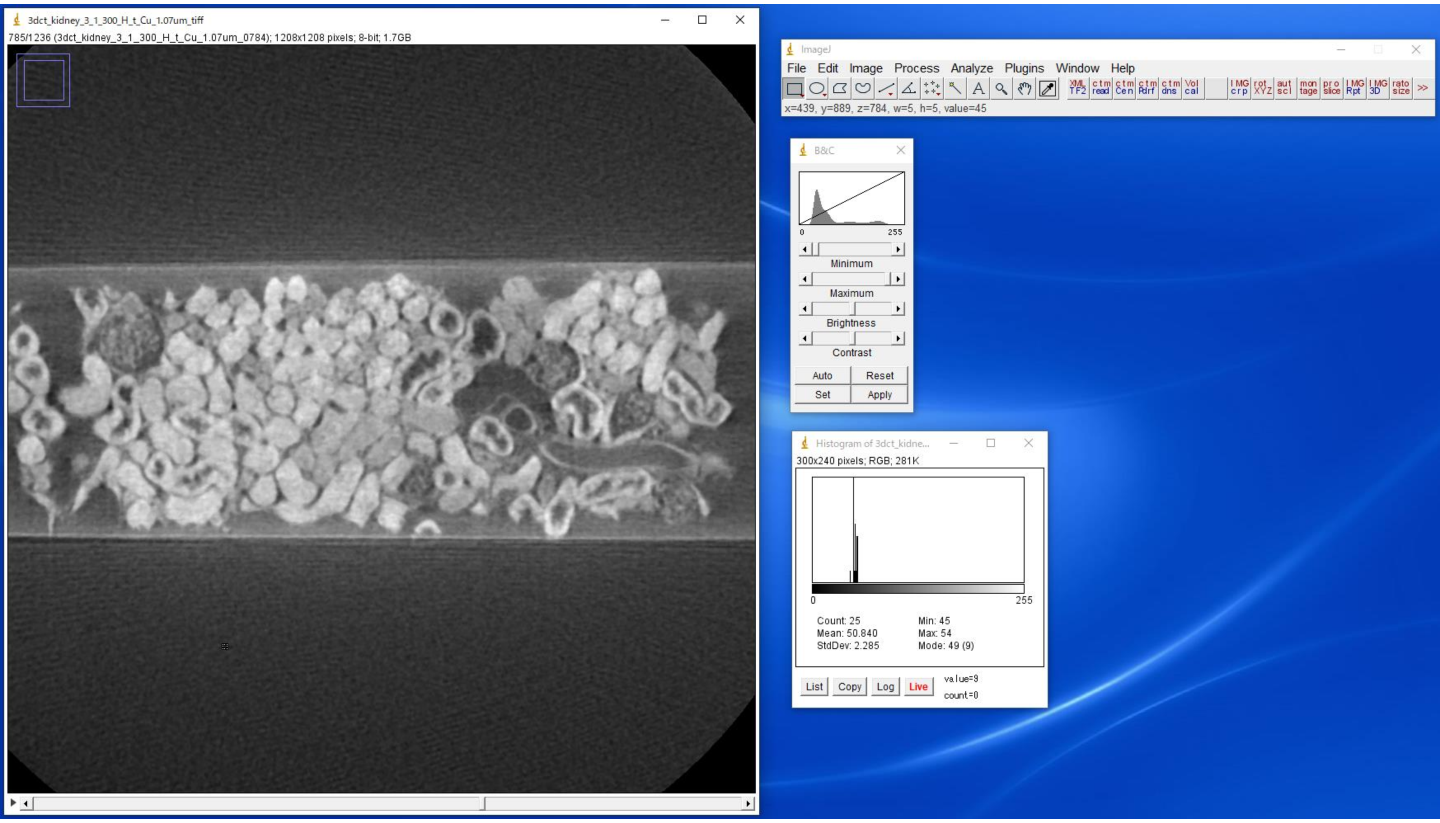

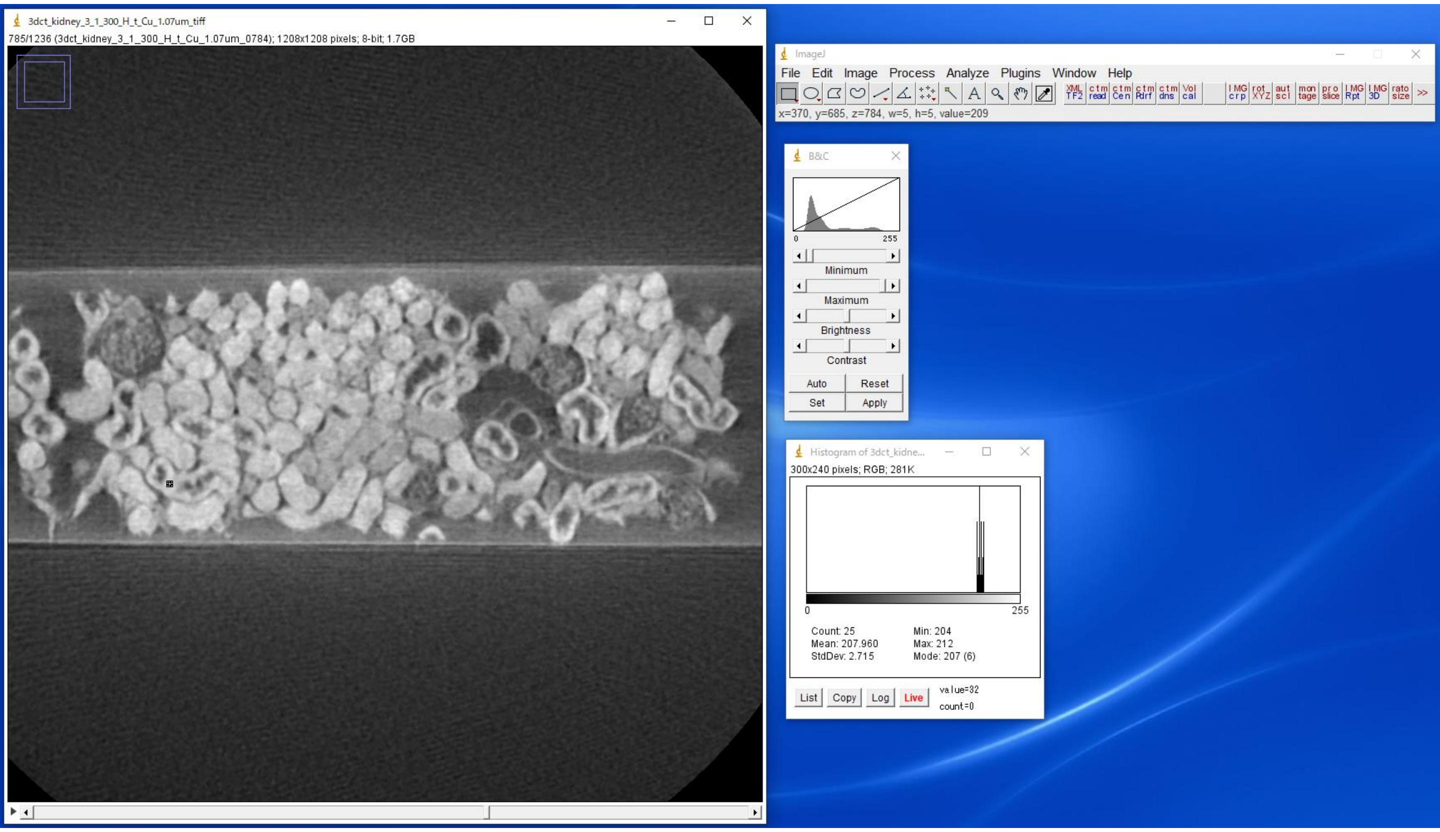

Supplementary Fig. 1. CNR measurement using the program *ImageJ* for CCD data.

Distal tubule measurement for 1_300_H_t nephron 4: air (top) and material (bottom) regions.

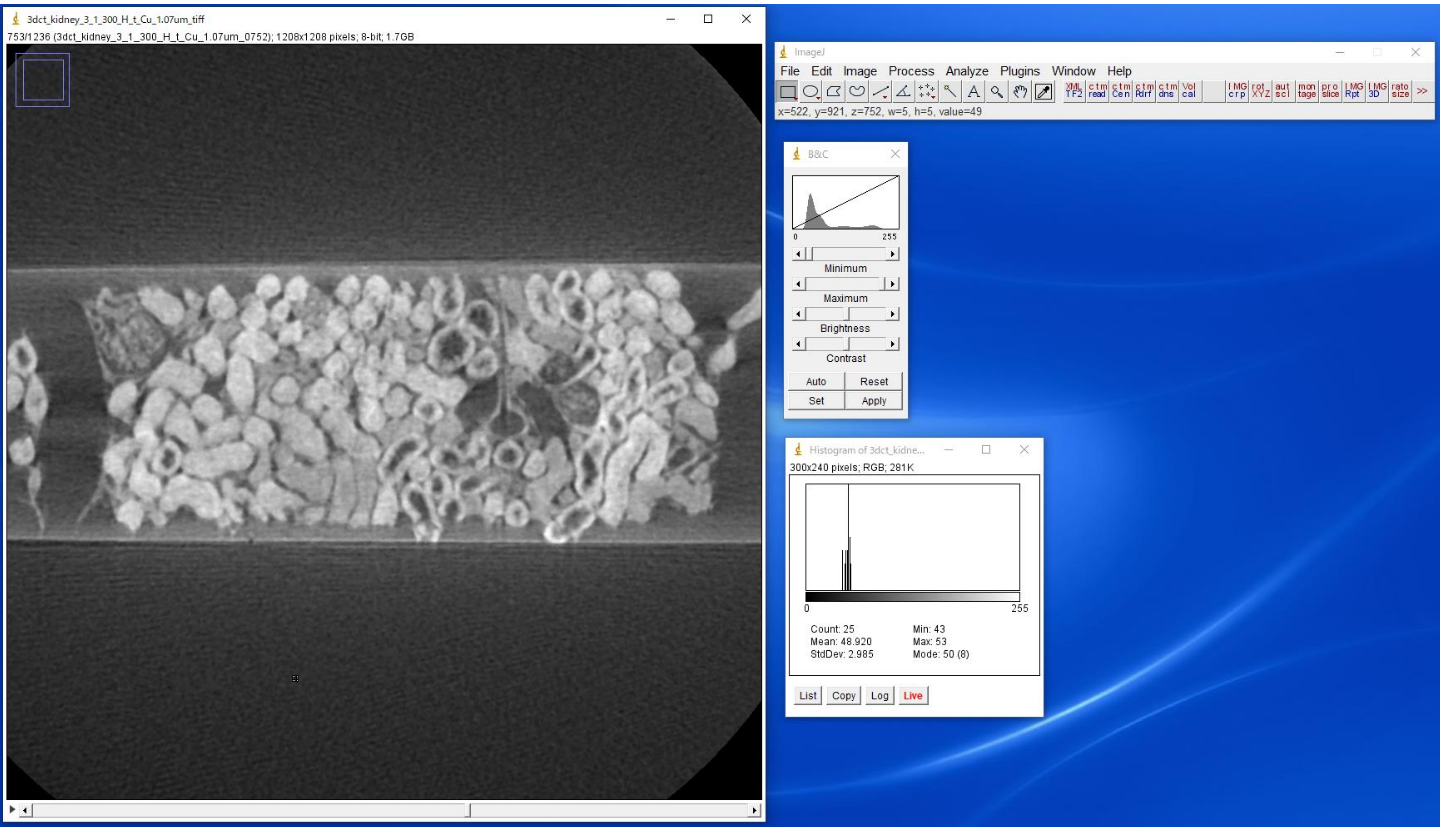

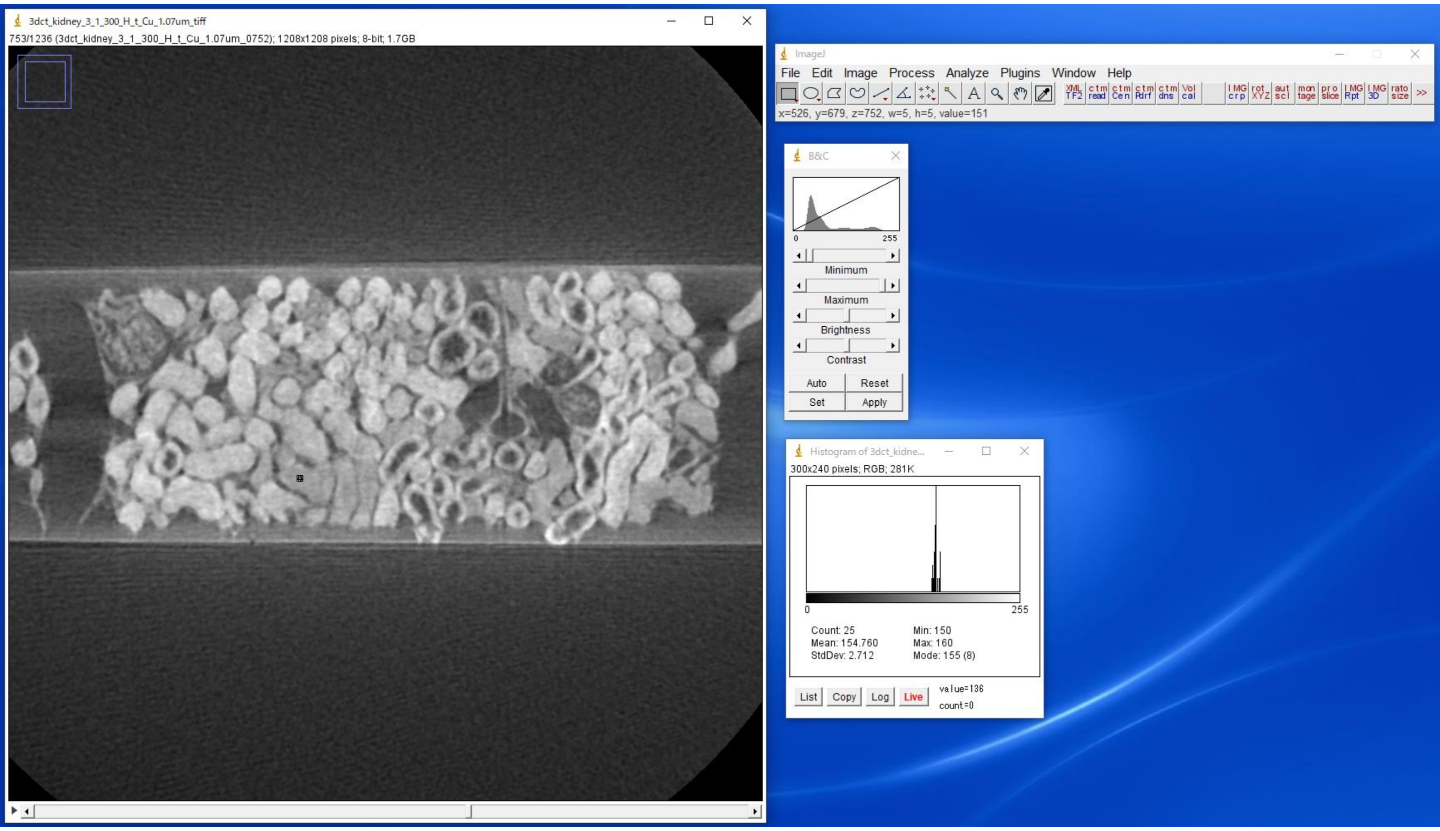

Supplementary Fig. 1. CNR measurement using the program *ImageJ* for CCD data.

Proximal tubule measurement for 1_300_H_t nephron 5: air (top) and material (bottom) regions.

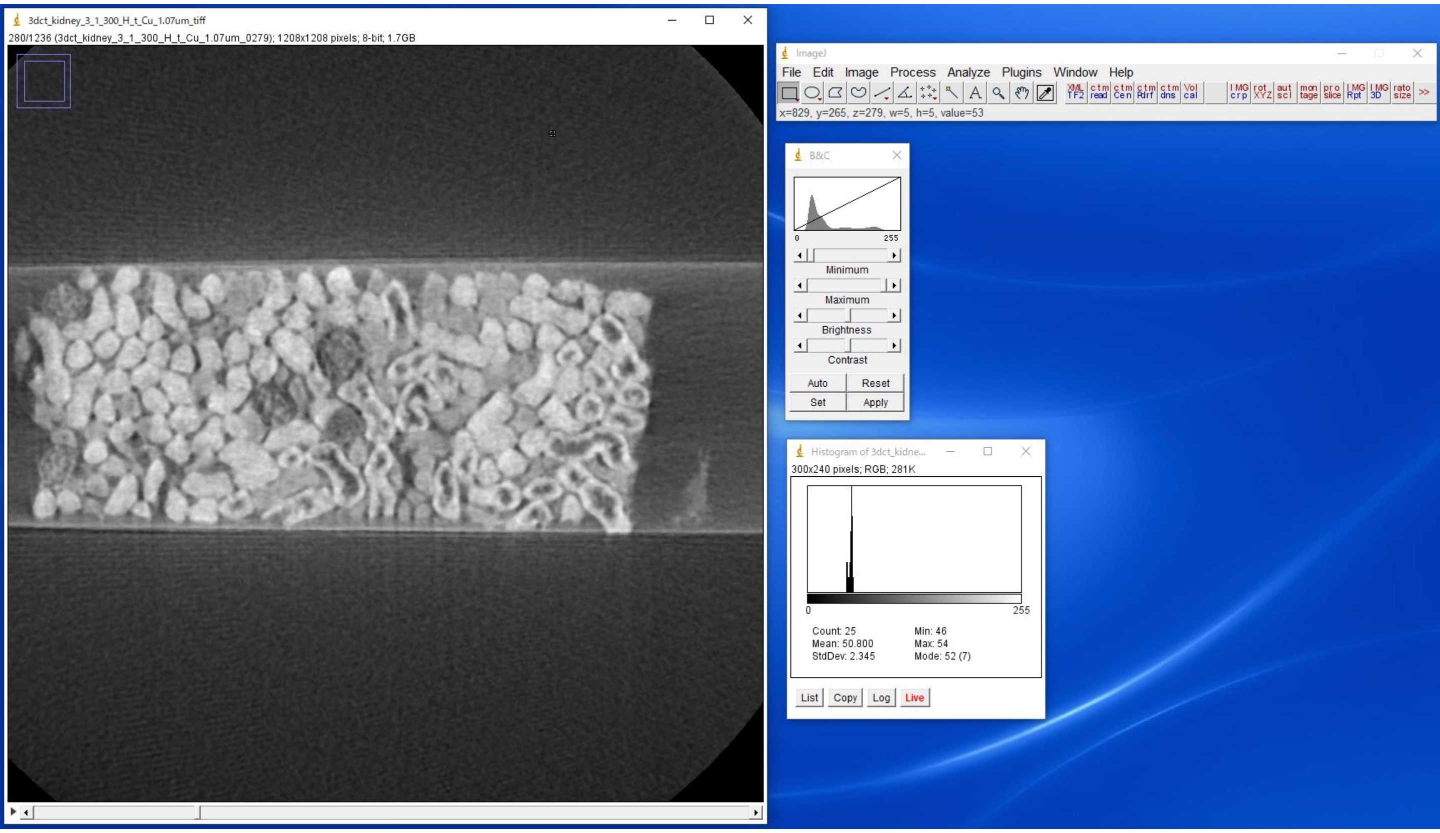

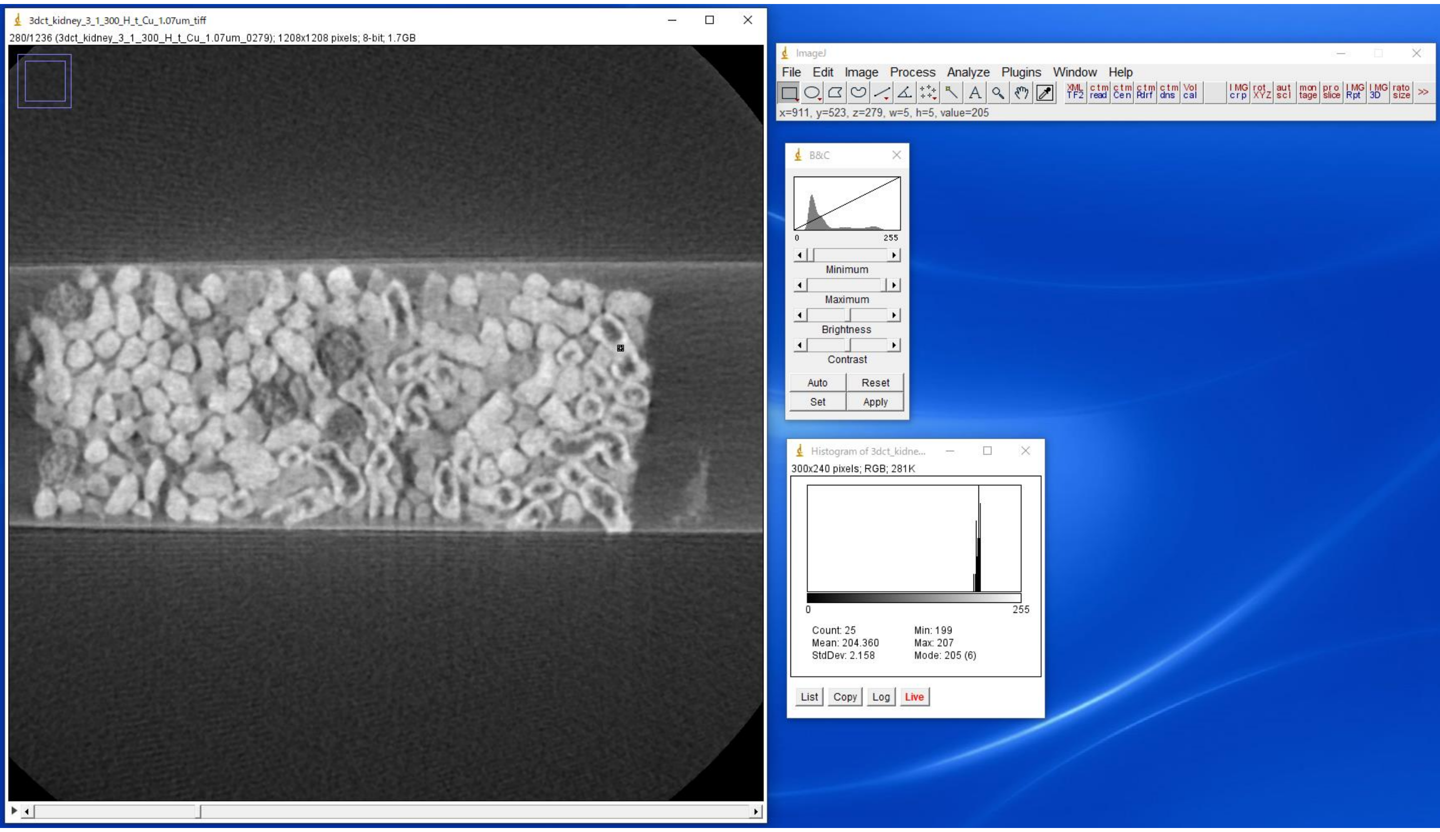

Supplementary Fig. 1. CNR measurement using the program *ImageJ* for CCD data.

Distal tubule measurement for 1_300_H_t nephron 5: air (top) and material (bottom) regions.

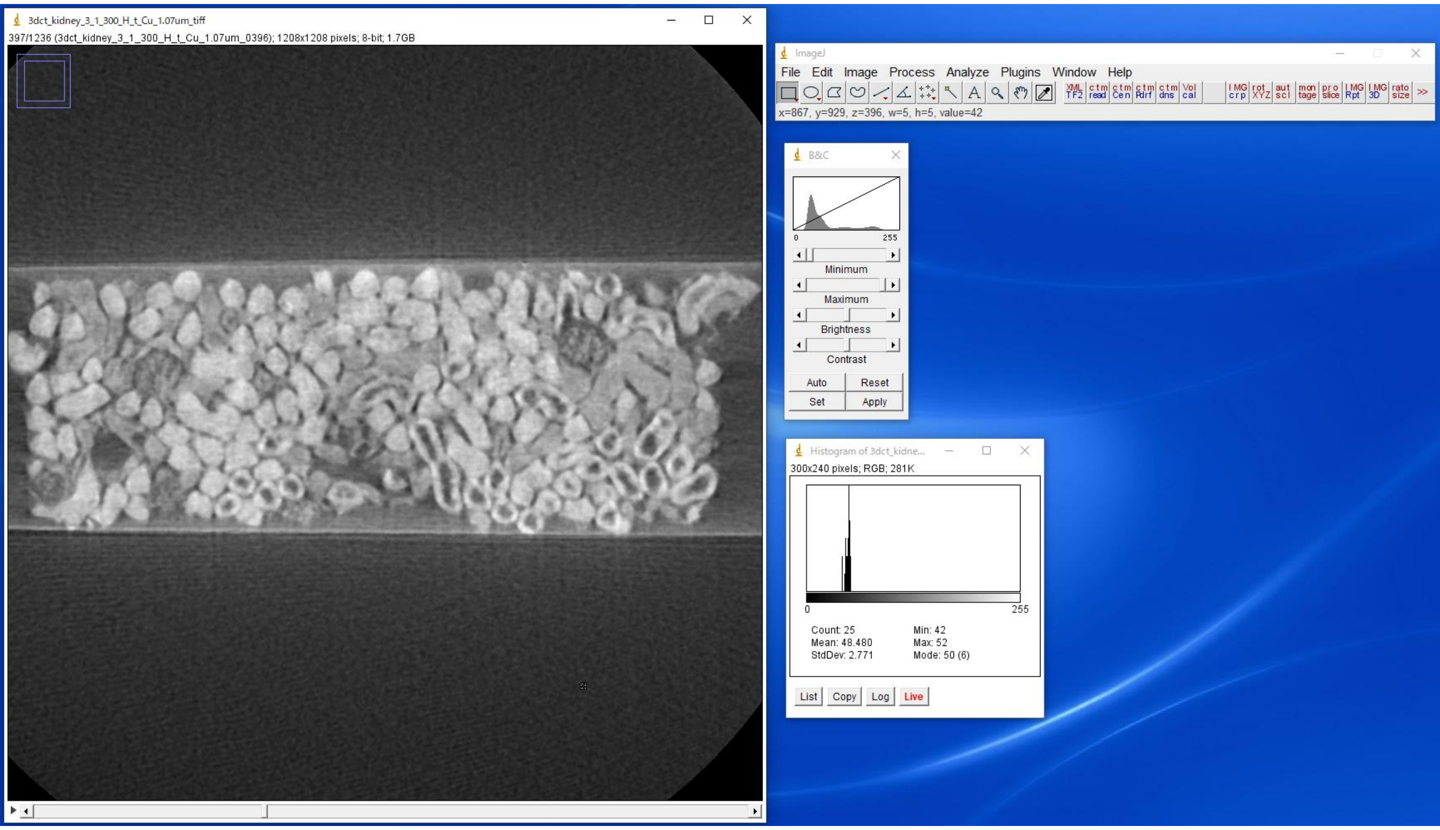

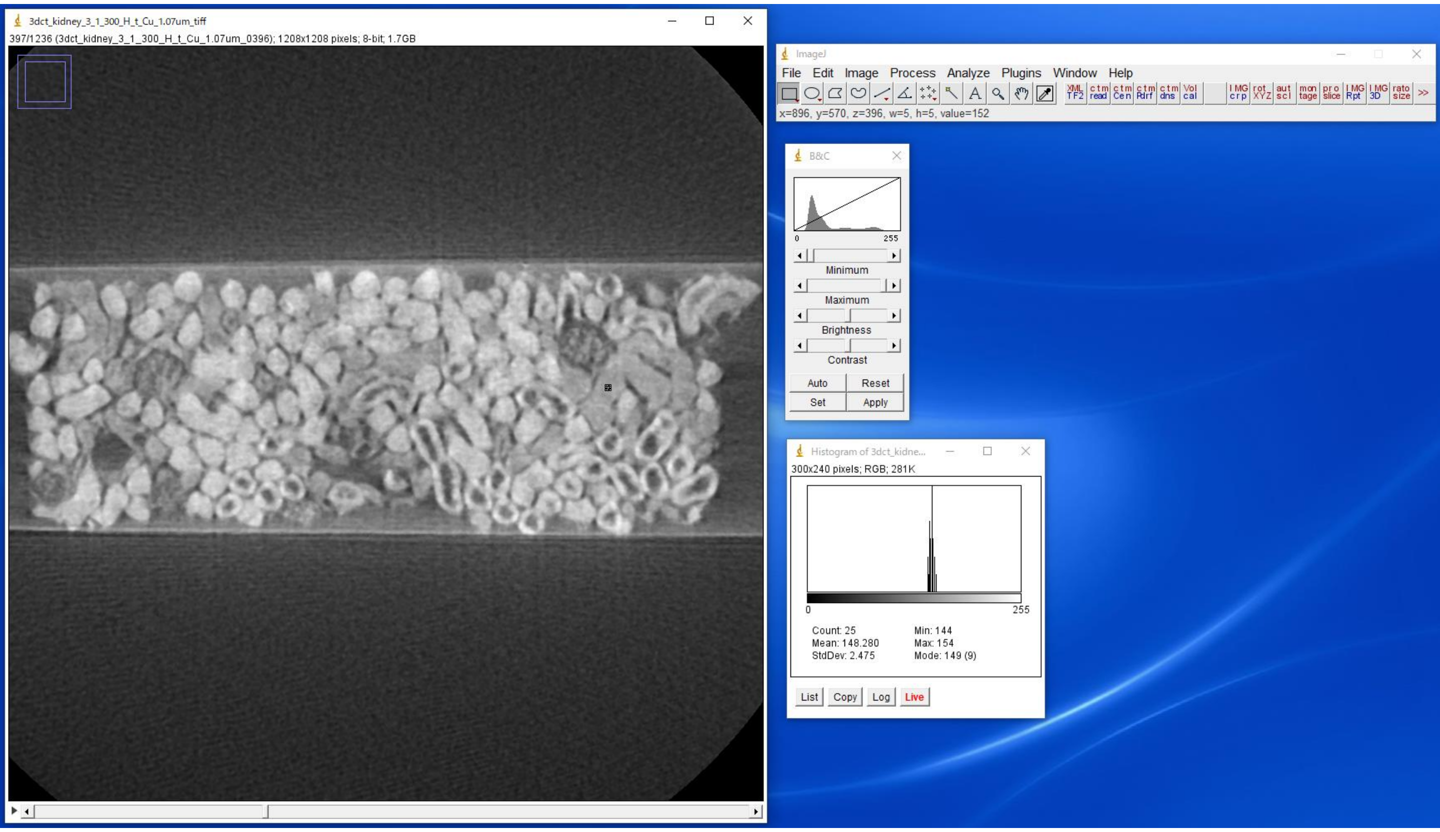

Supplementary Fig. 1. CNR measurement using the program *ImageJ* for CCD data.

Proximal tubule measurement for 2_300_H_b nephron 1: air (top) and material (bottom) regions.

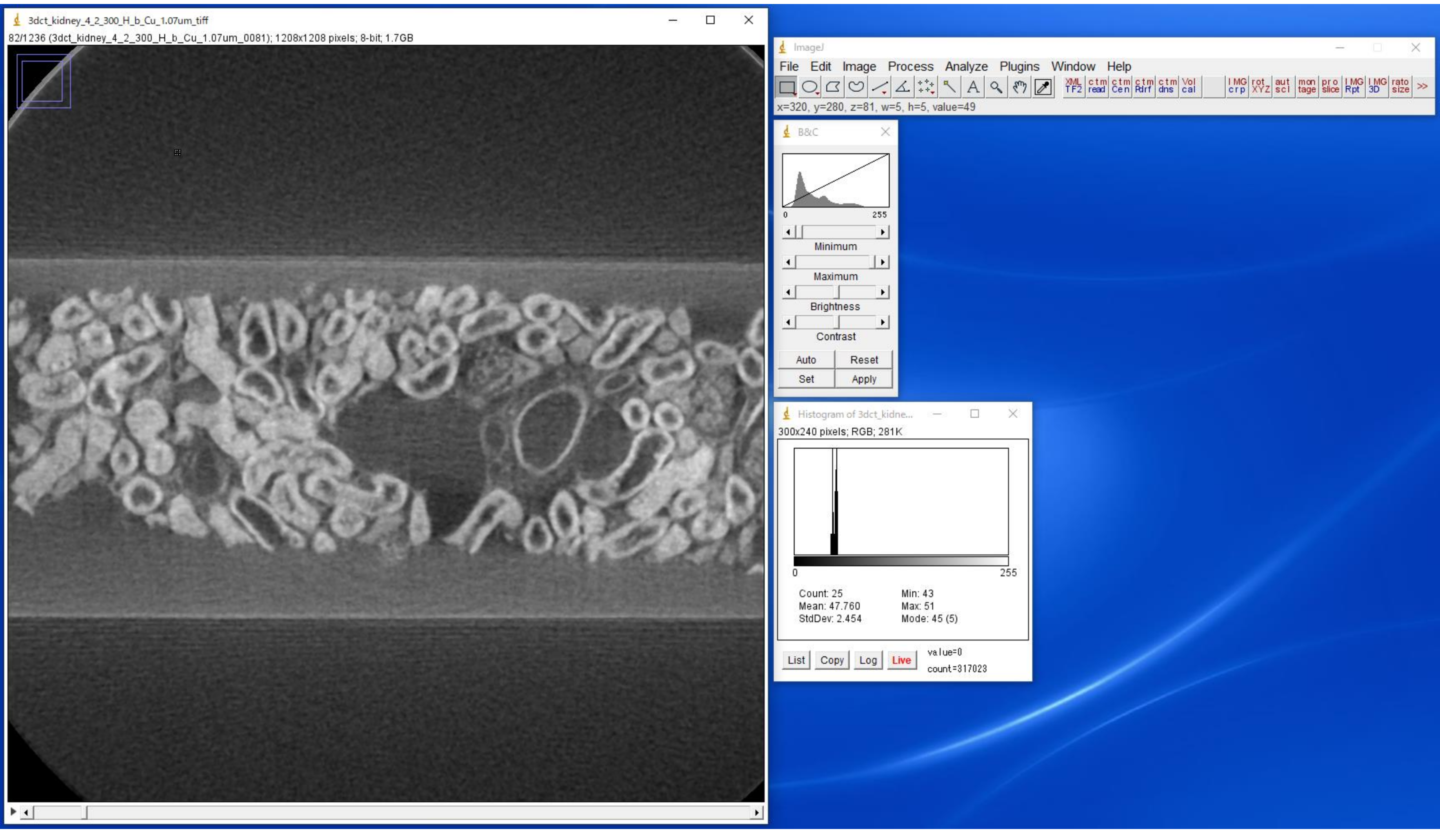

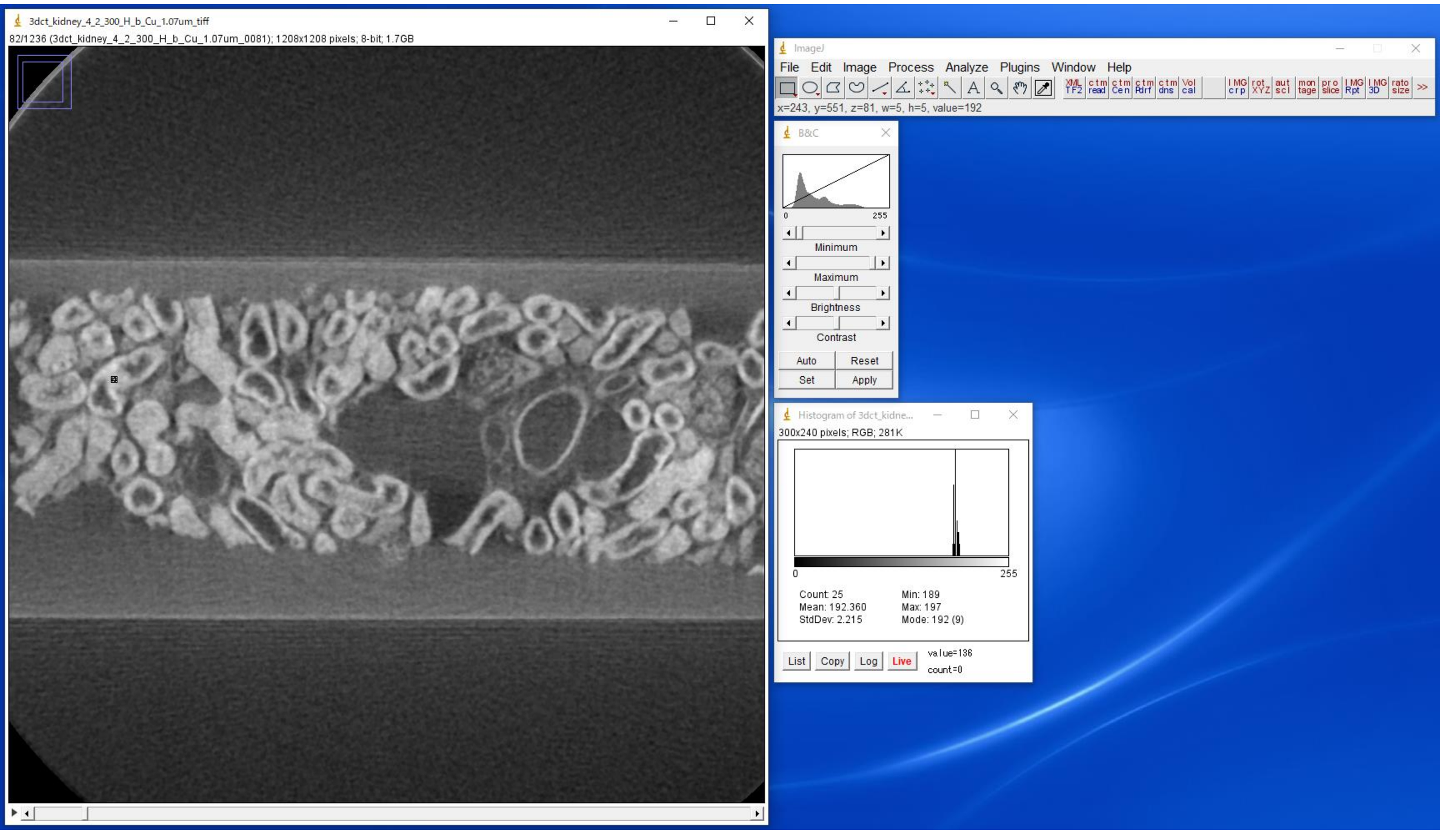

Supplementary Fig. 1. CNR measurement using the program *ImageJ* for CCD data.

Distal tubule measurement for 2_300_H_b nephron 1: air (top) and material (bottom) regions.

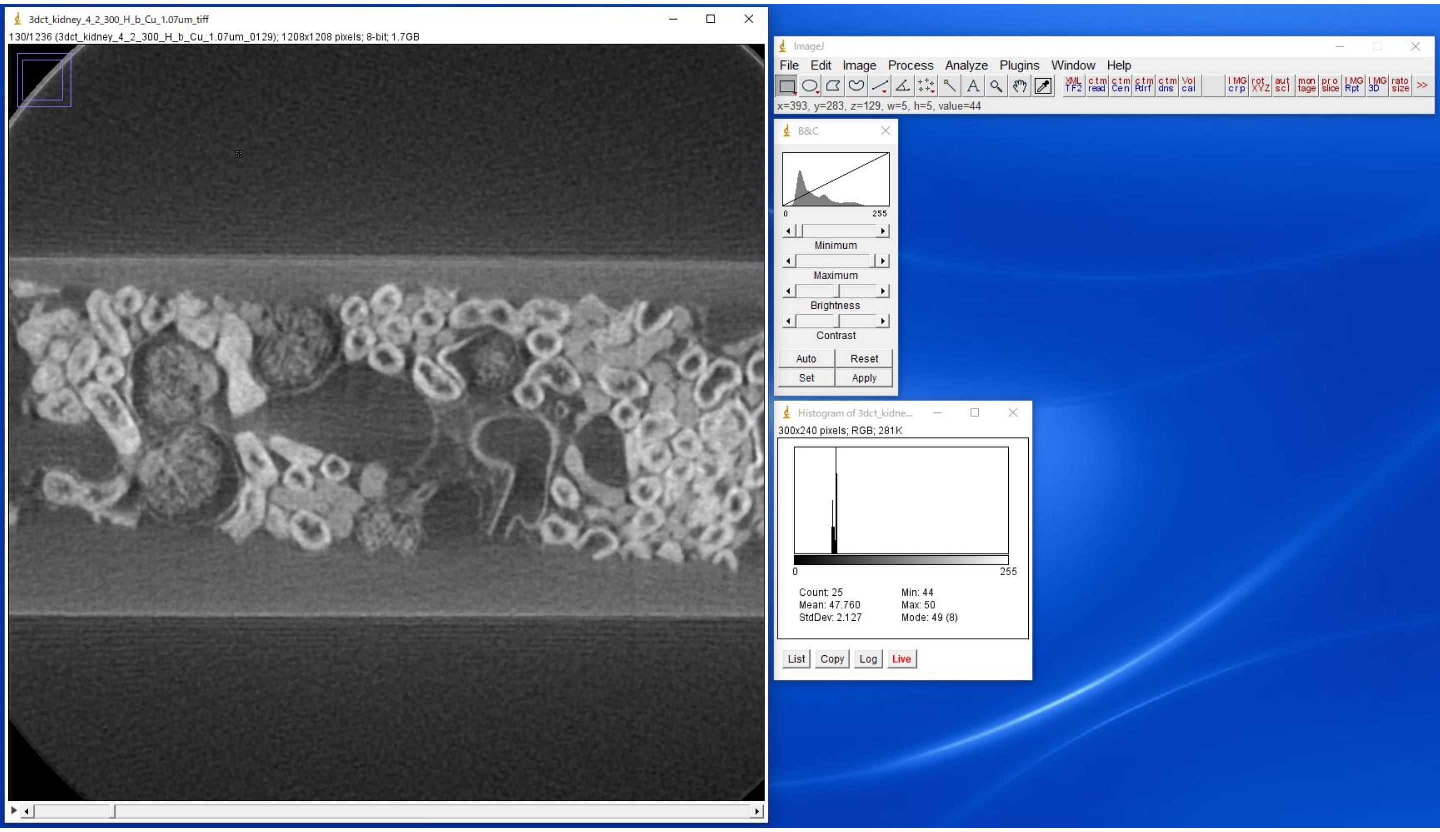

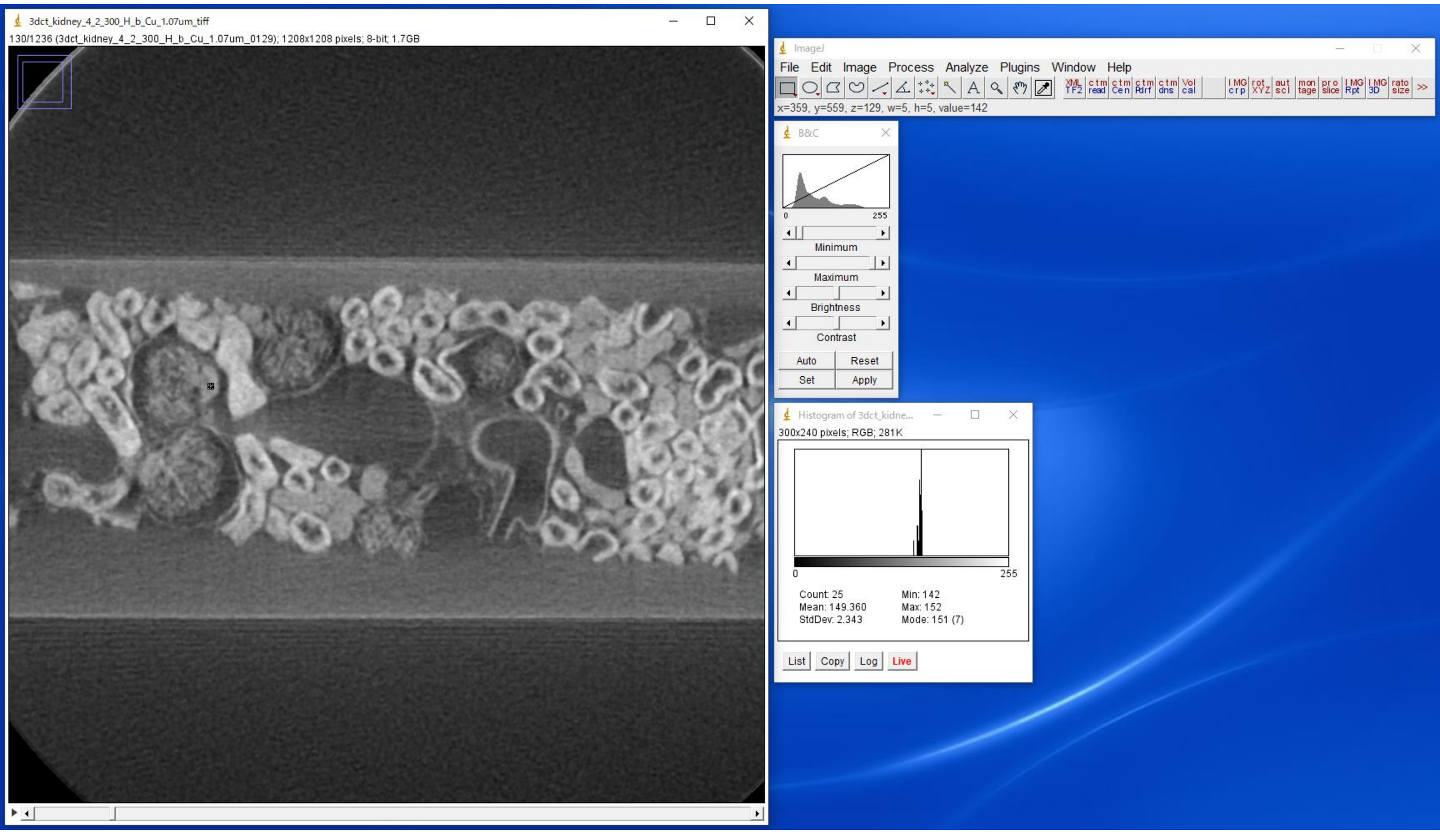

Supplementary Fig. 1. CNR measurement using the program *ImageJ* for CCD data.

Proximal tubule measurement for 2_300_H_b nephron 2: air (top) and material (bottom) regions.

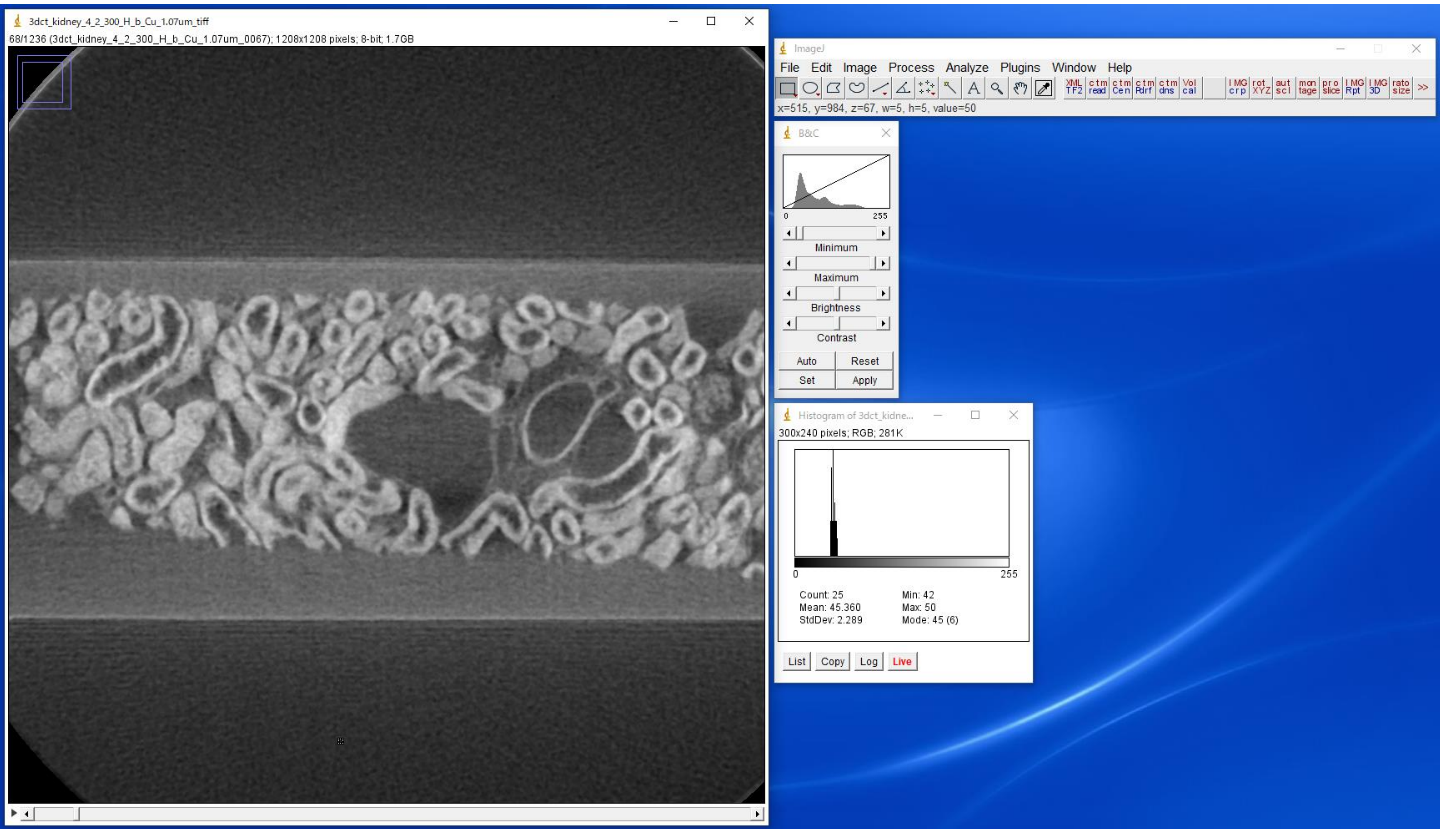

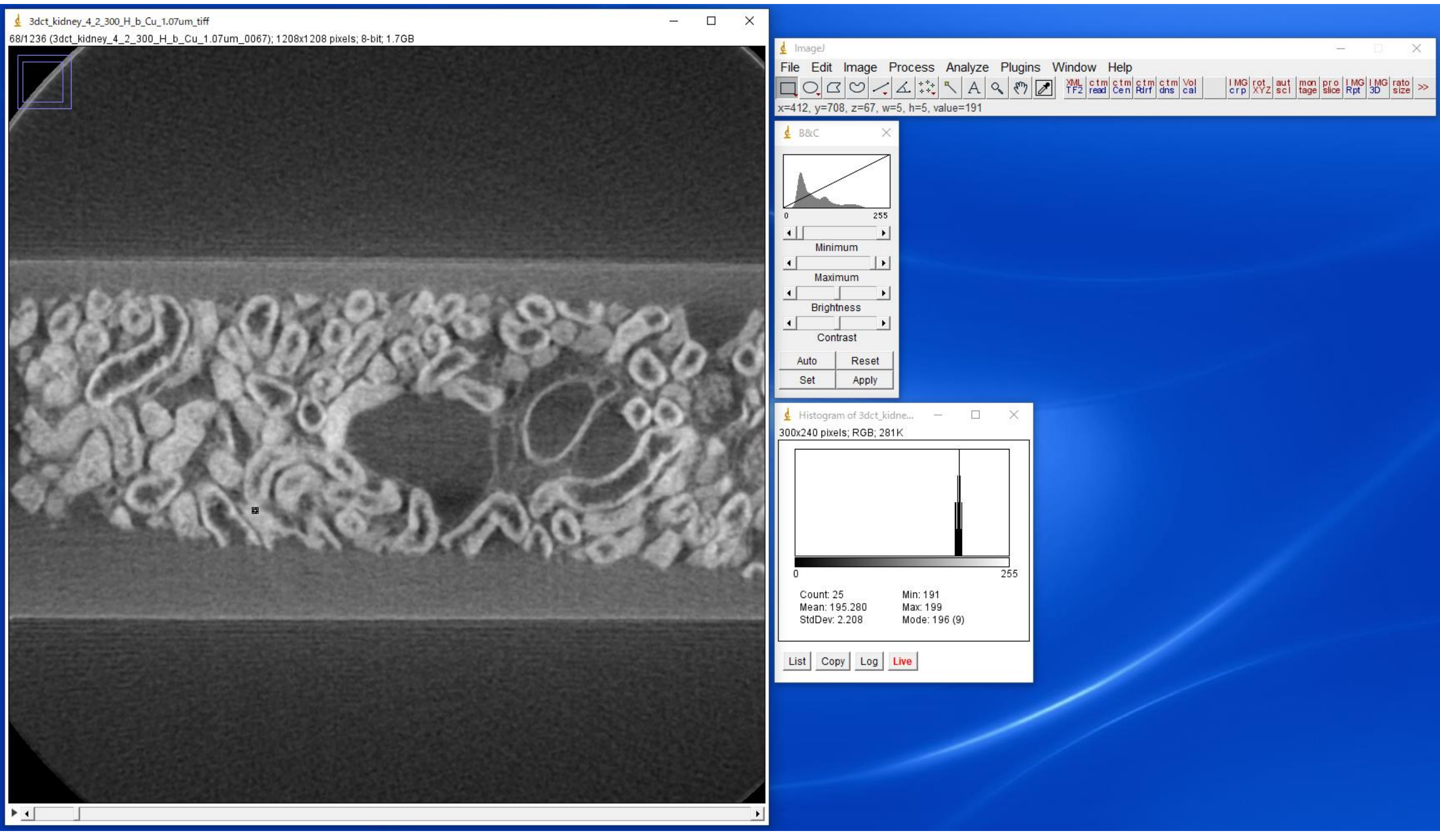

Supplementary Fig. 1. CNR measurement using the program *ImageJ* for CCD data.

Distal tubule measurement for 2_300_H_b nephron 2: air (top) and material (bottom) regions.

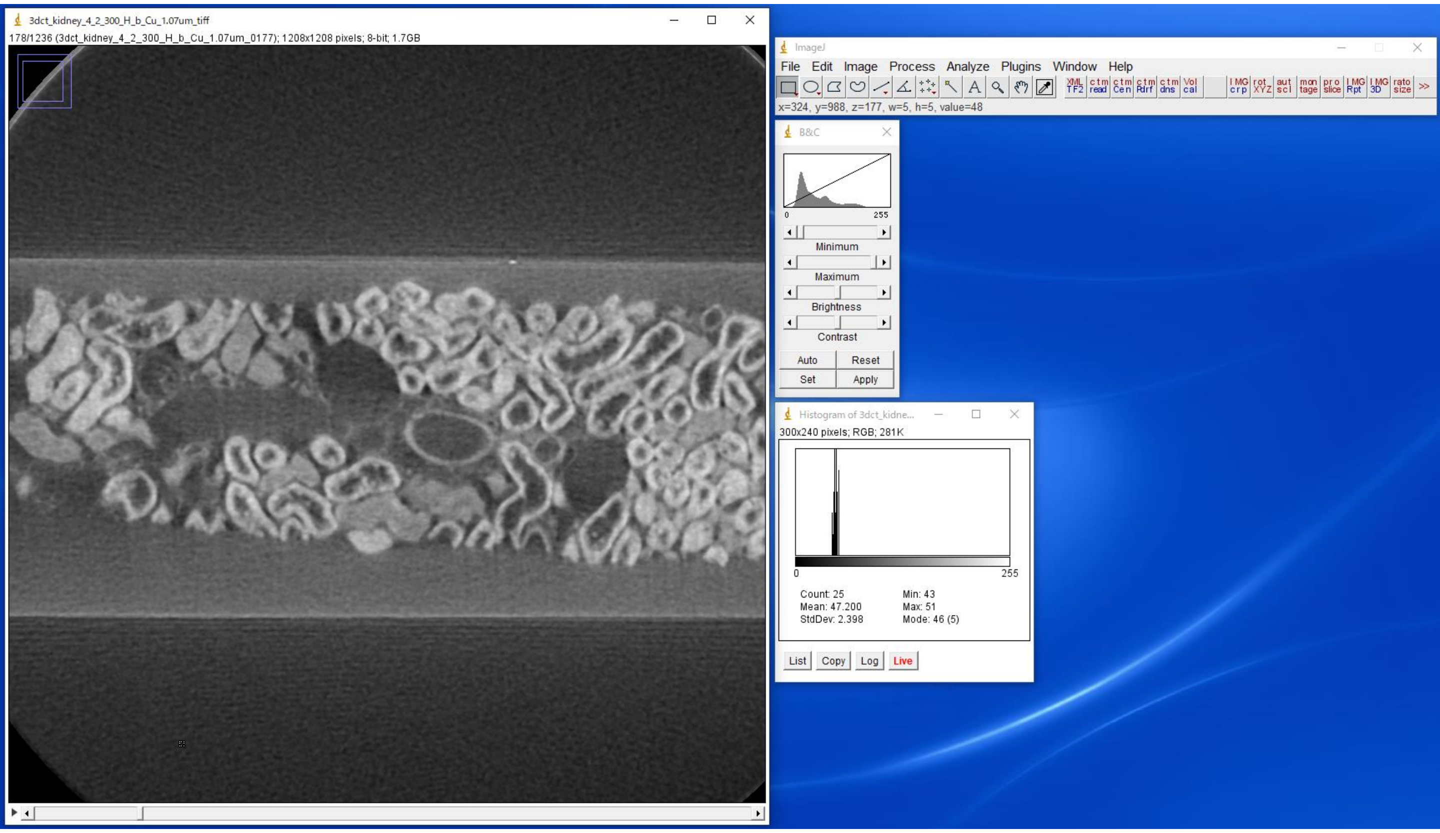

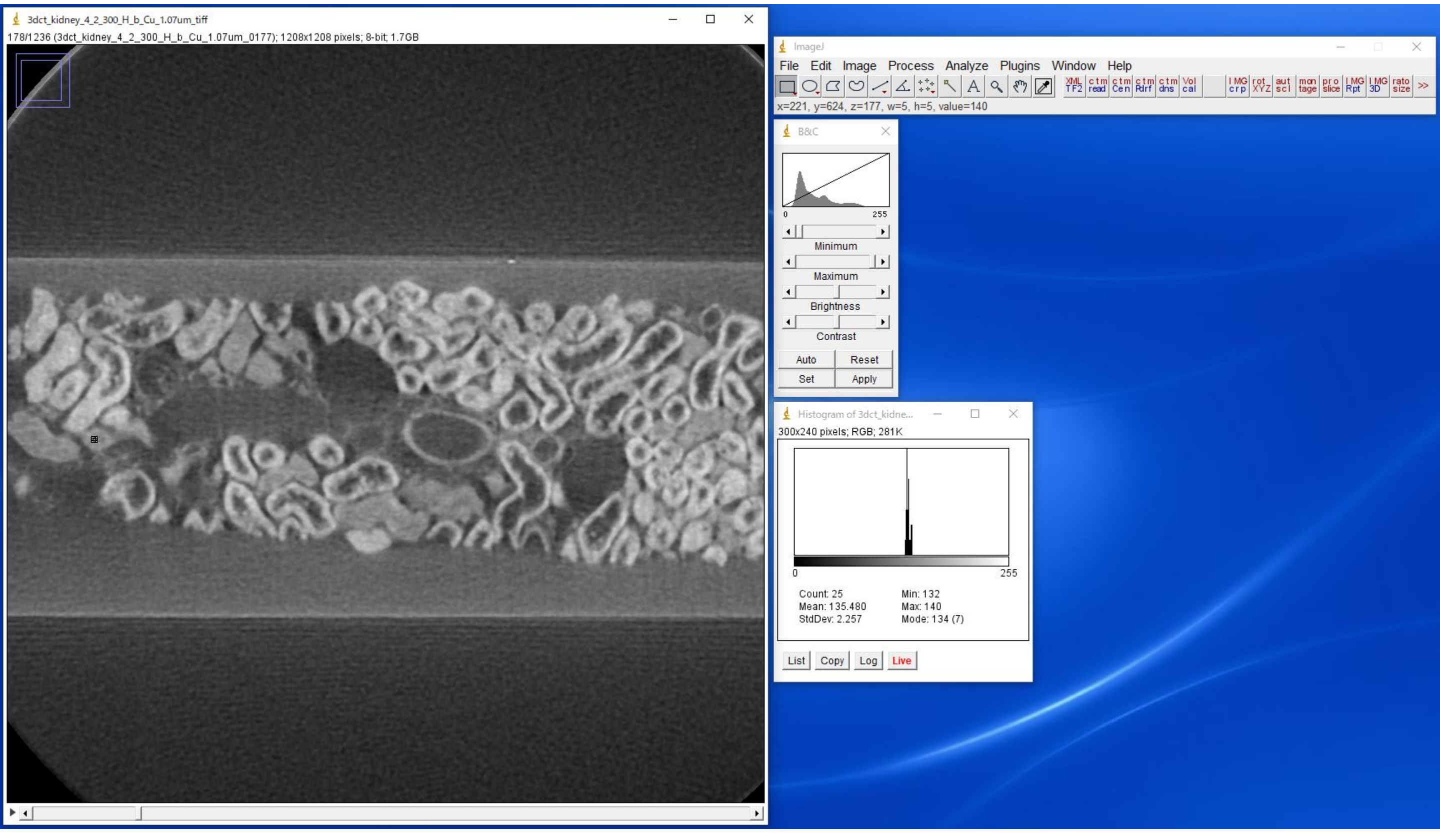

Supplementary Fig. 1. CNR measurement using the program *ImageJ* for CCD data.

Proximal tubule measurement for 2_300_H_b nephron 3: air (top) and material (bottom) regions.

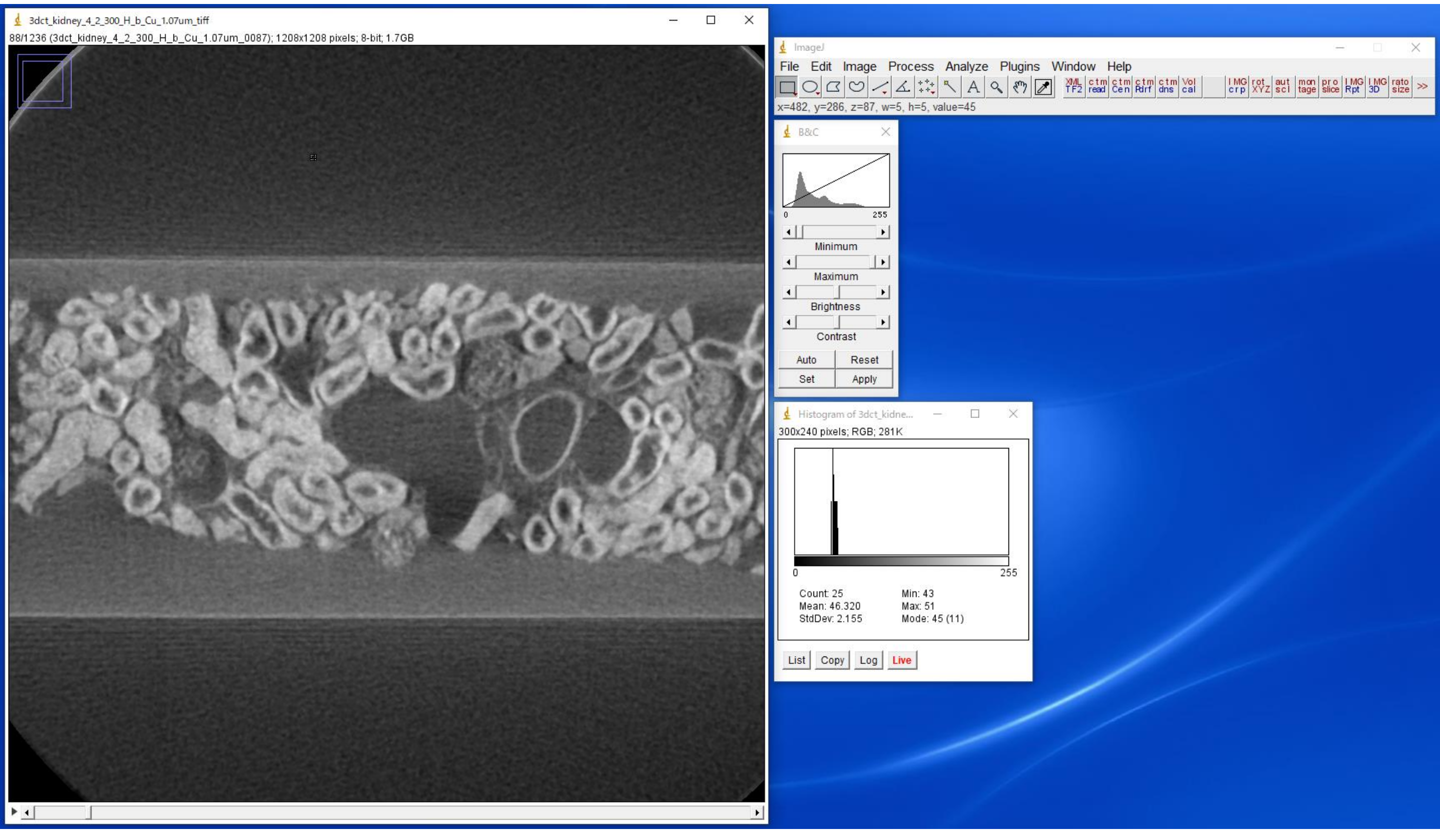

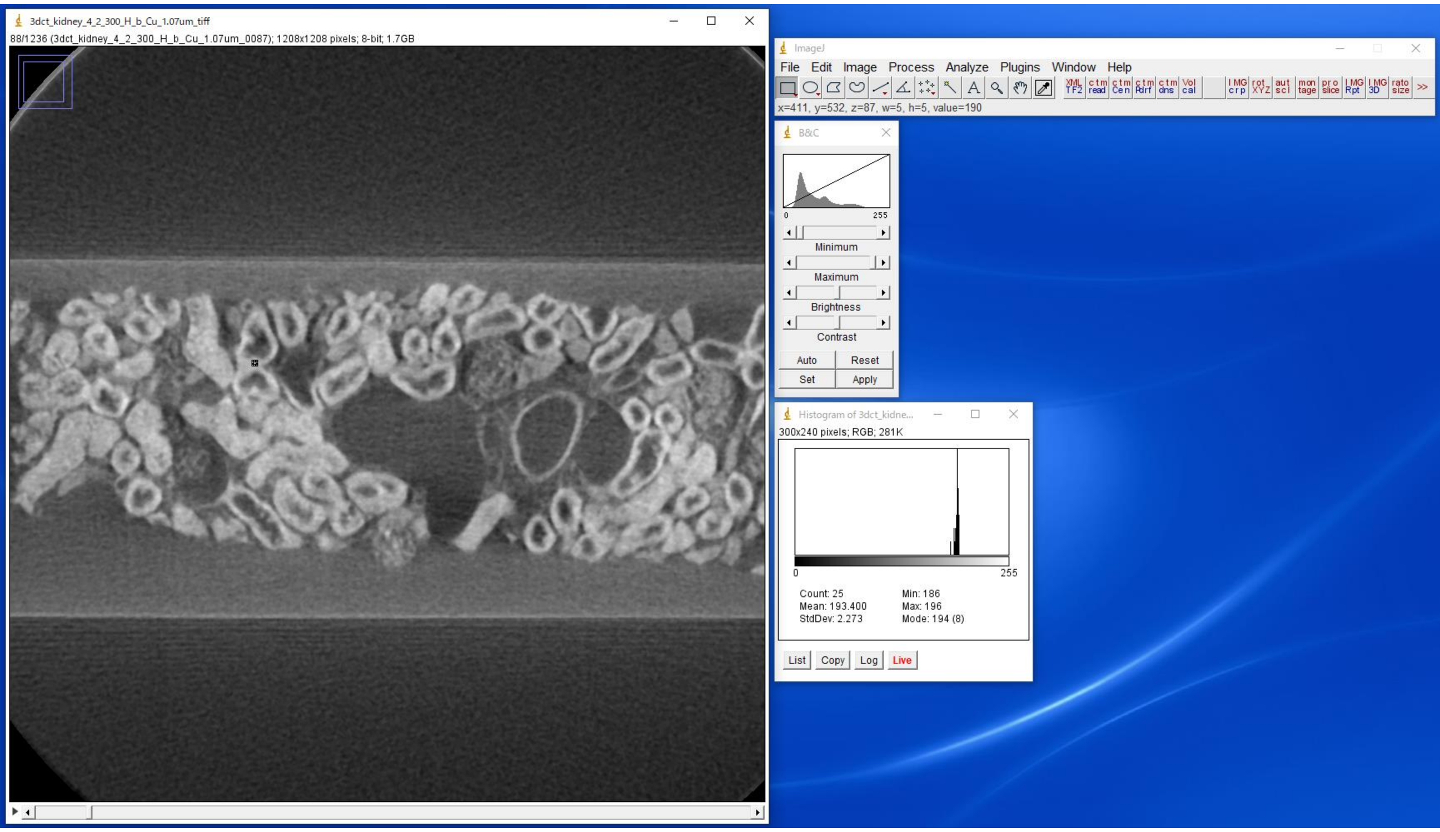

Supplementary Fig. 1. CNR measurement using the program *ImageJ* for CCD data.

Distal tubule measurement for 2_300_H_b nephron 3: air (top) and material (bottom) regions.

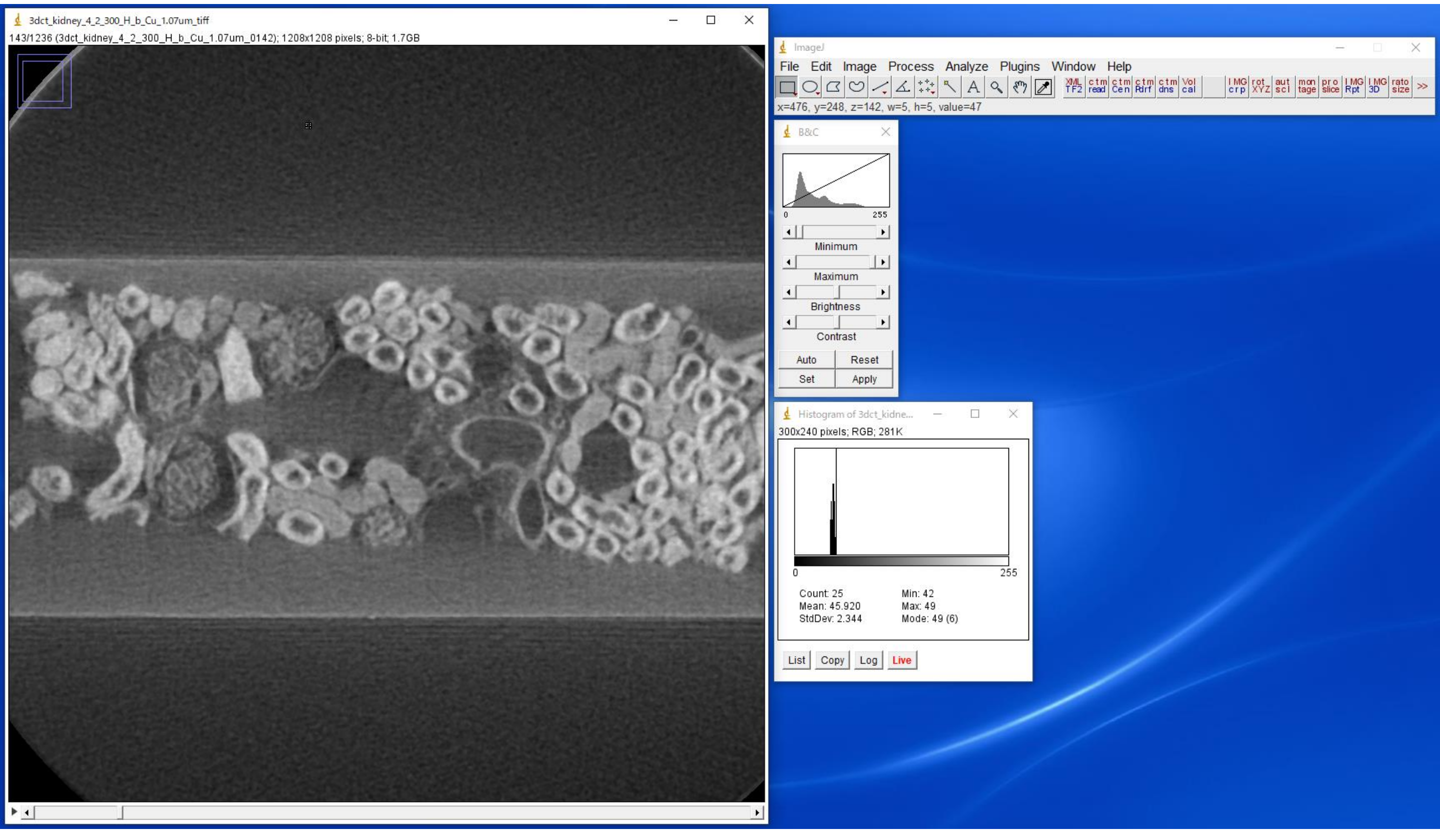

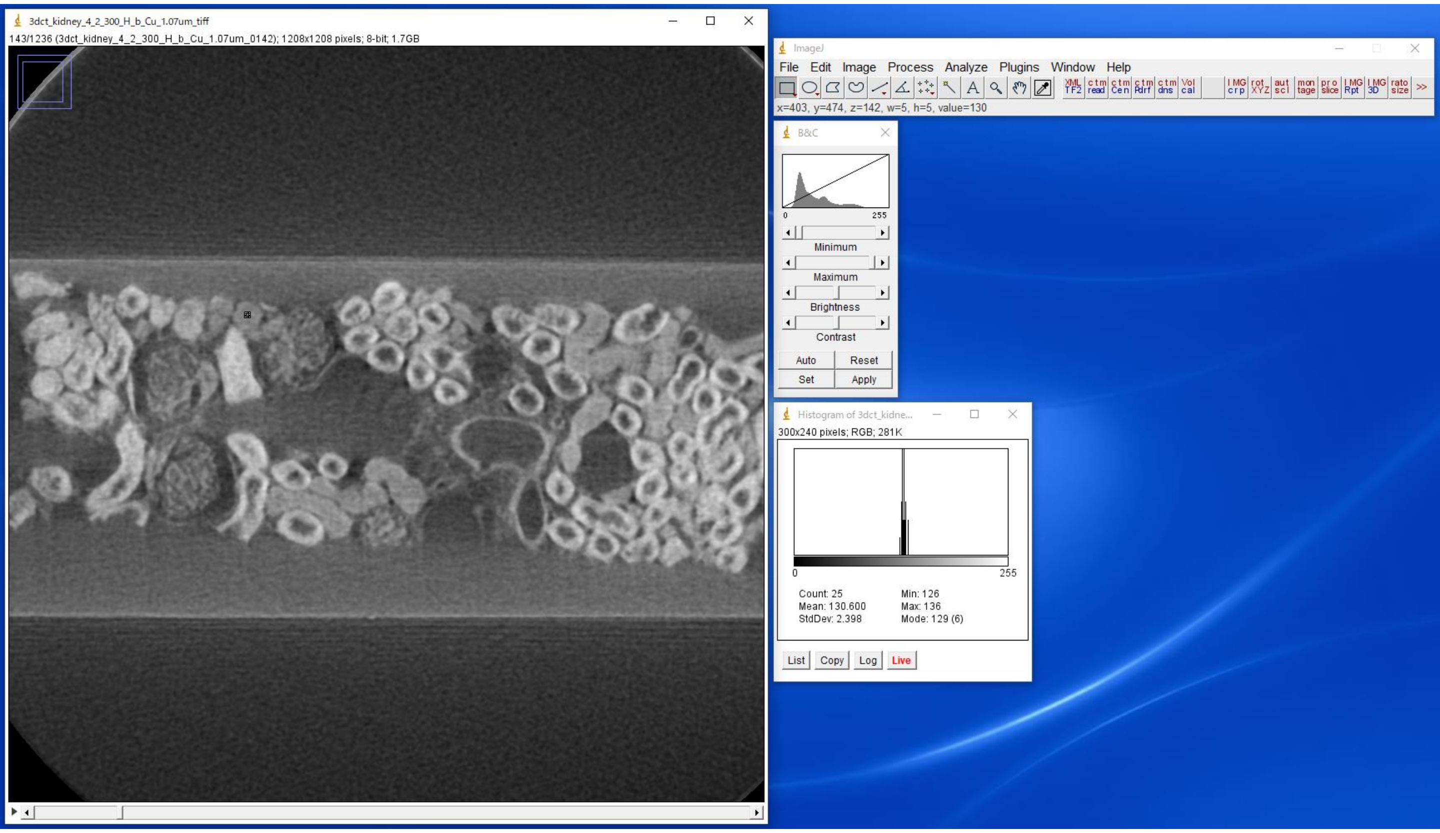

Supplementary Fig. 1. CNR measurement using the program *ImageJ* for CCD data.

Proximal tubule measurement for 2_300_H_b nephron 4: air (top) and material (bottom) regions.

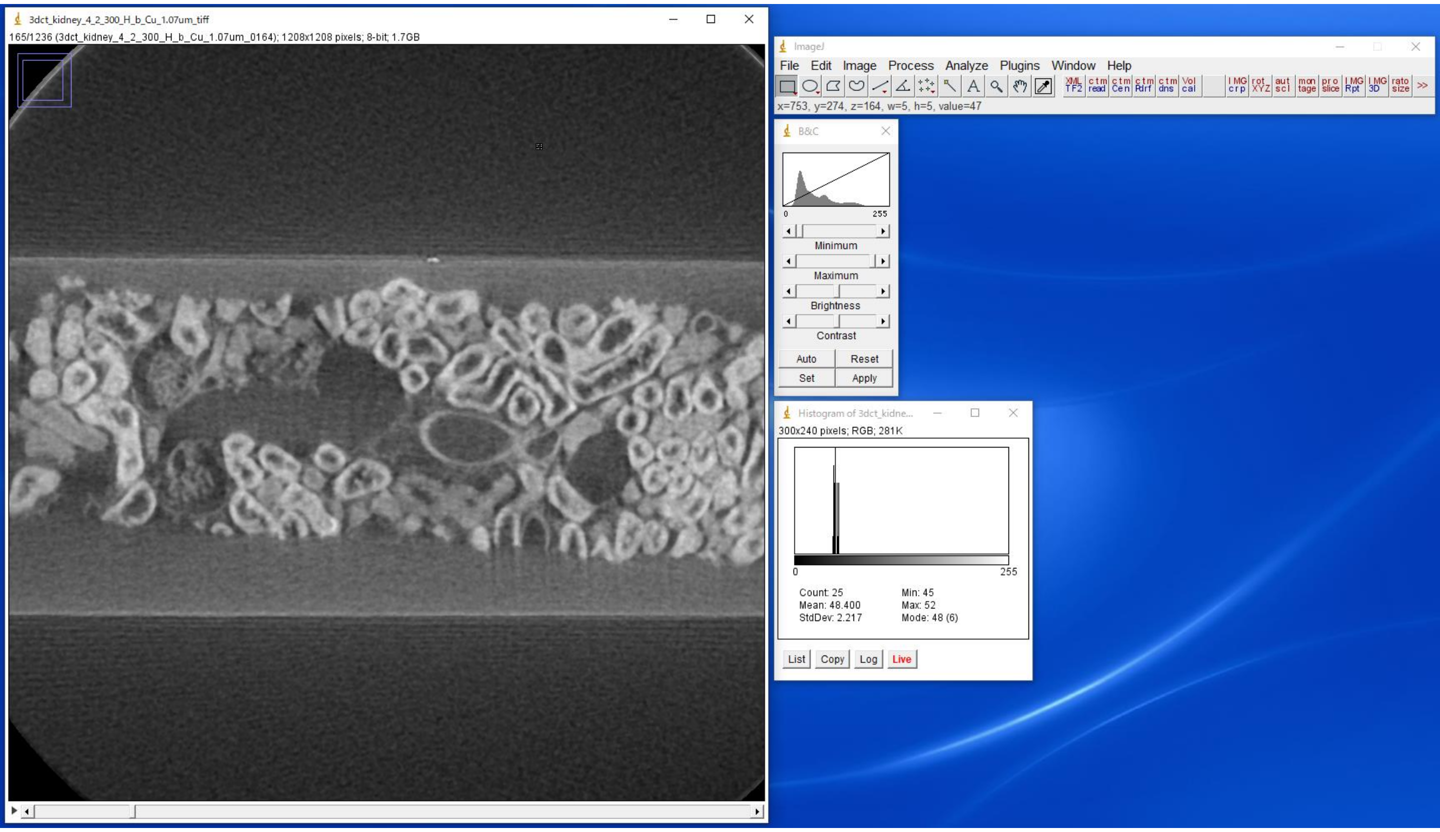

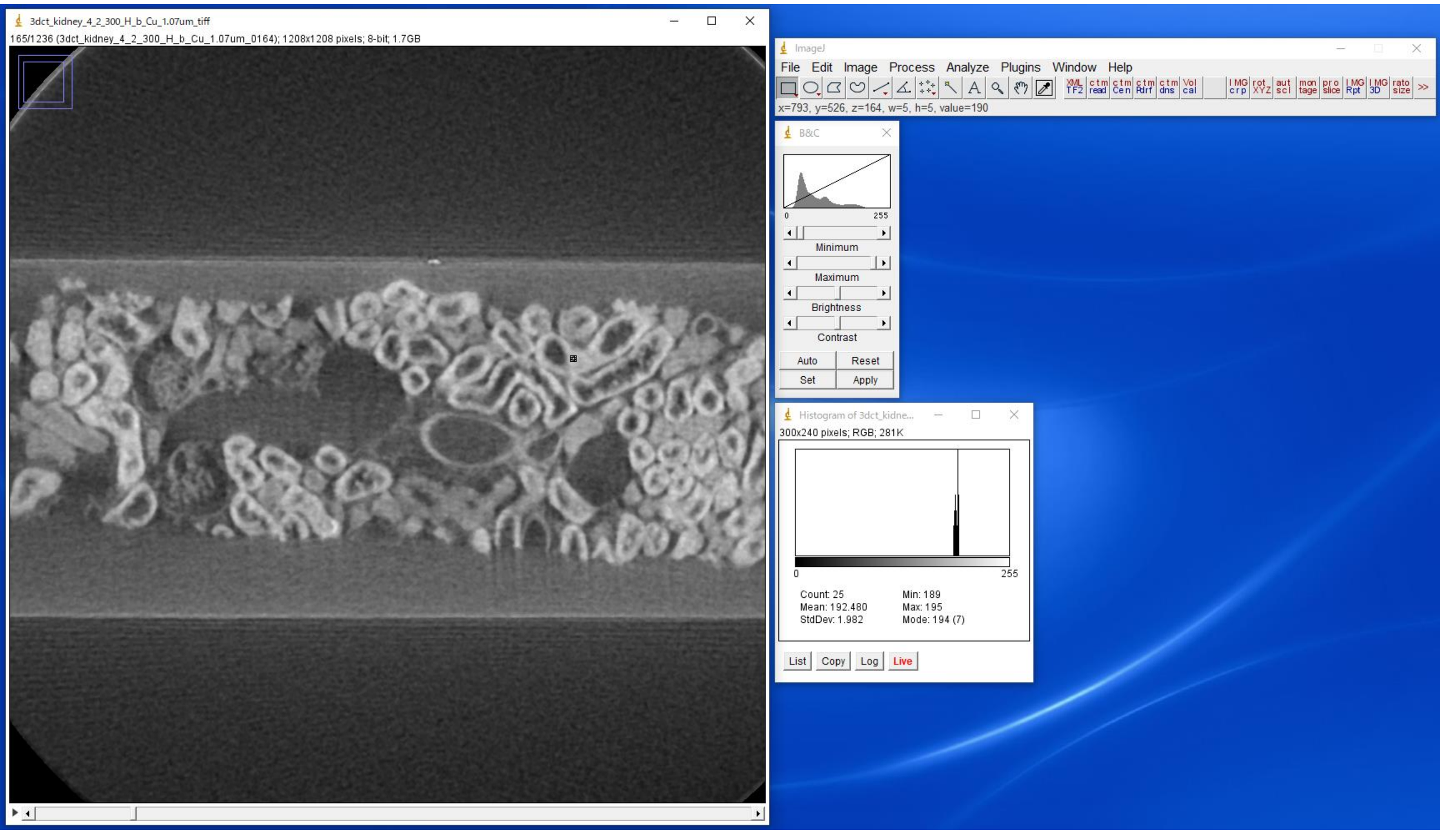

Supplementary Fig. 1. CNR measurement using the program *ImageJ* for CCD data.

Distal tubule measurement for 2_300_H_b nephron 4: air (top) and material (bottom) regions.

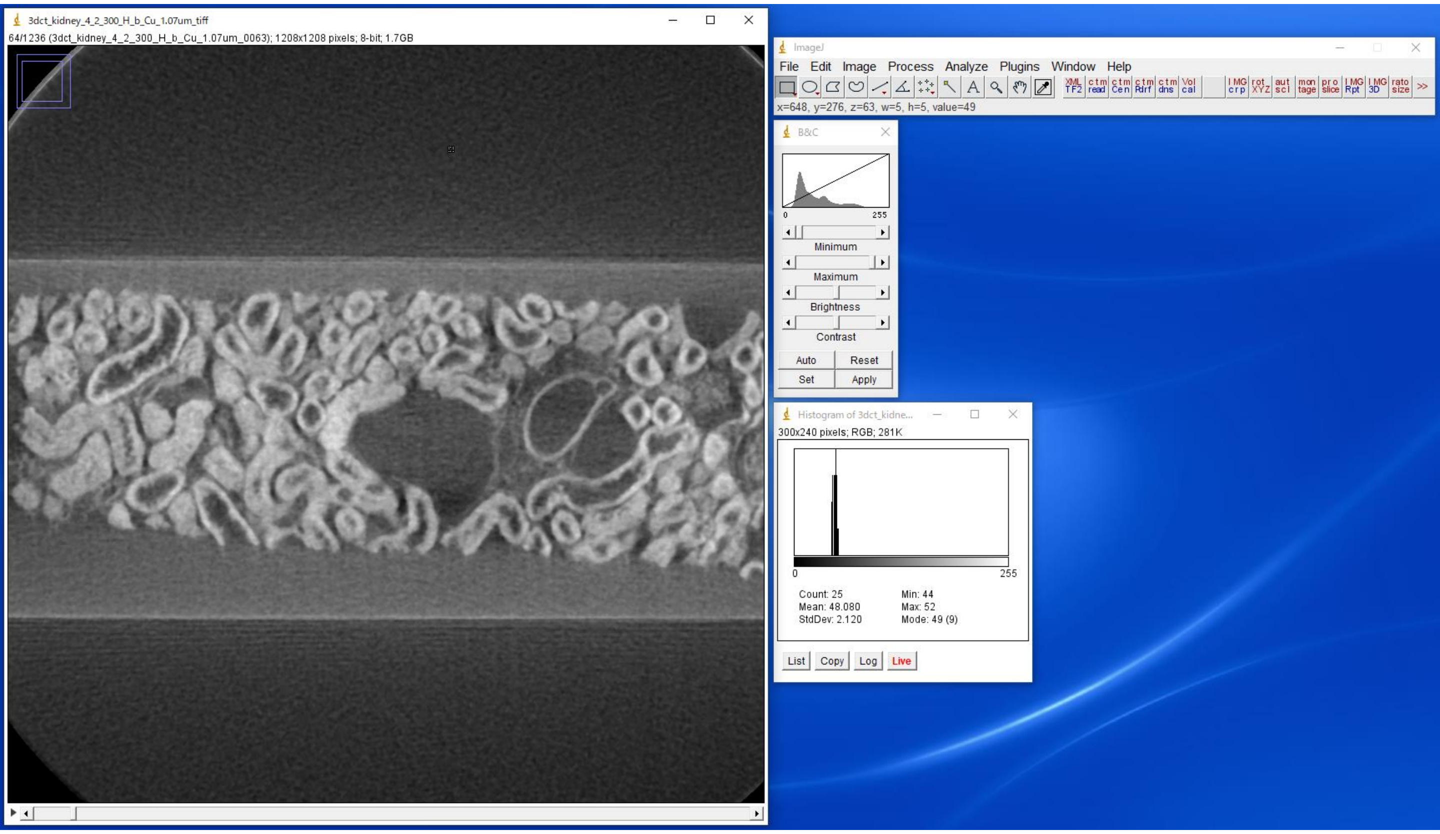

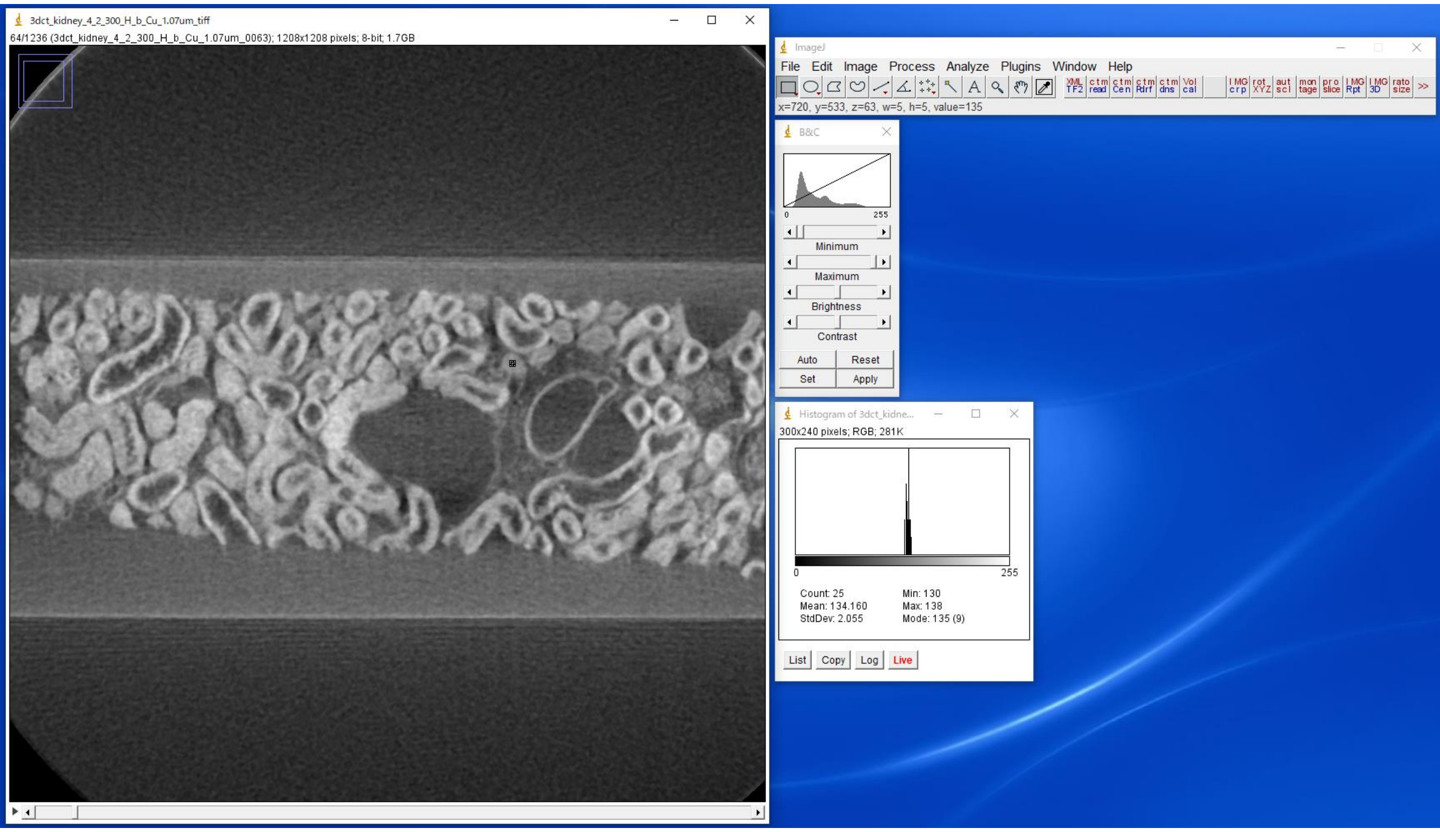

Supplementary Fig. 1. CNR measurement using the program *ImageJ* for CCD data.

Proximal tubule measurement for 2_300_H_b nephron 5: air (top) and material (bottom) regions.

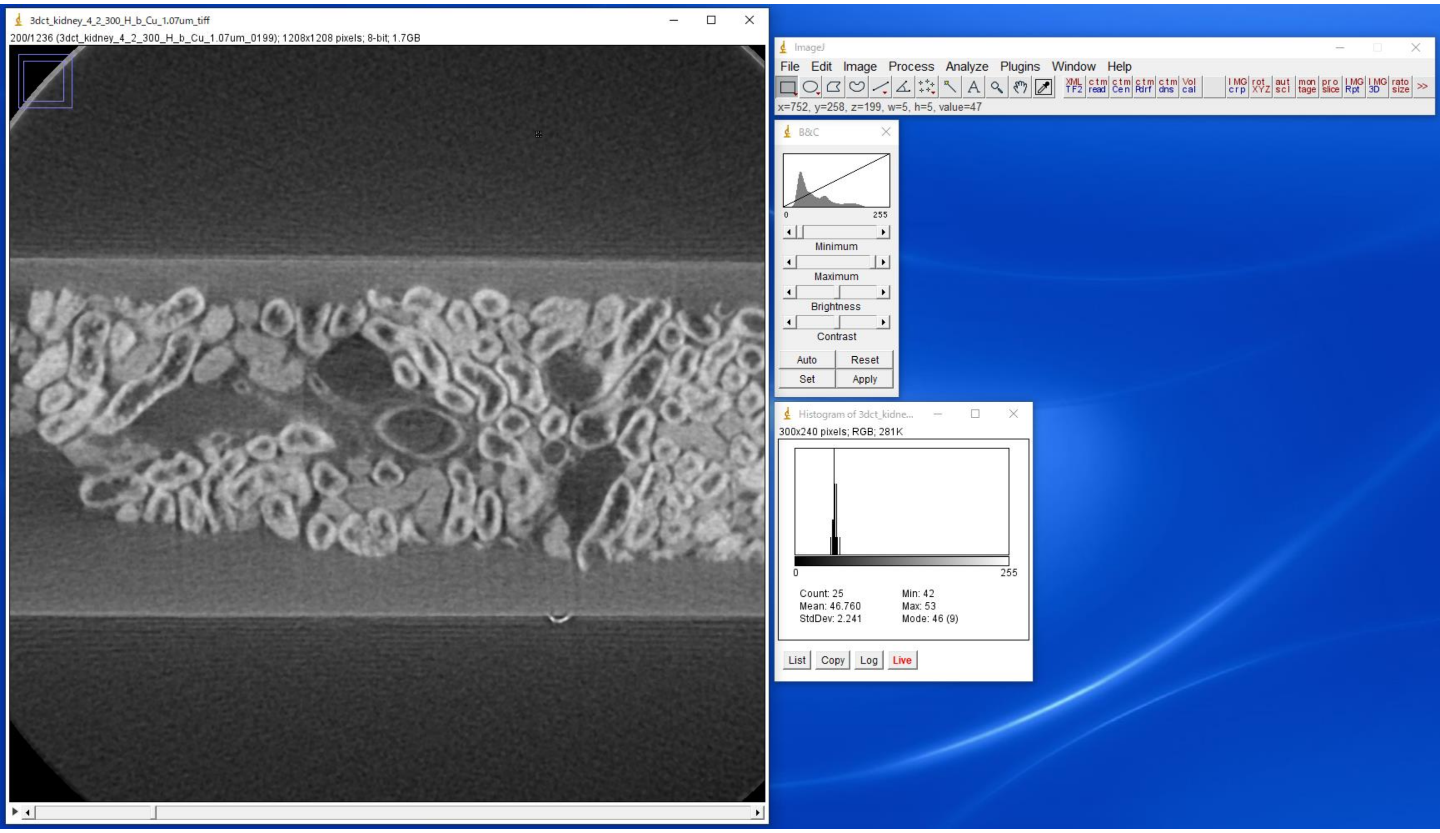

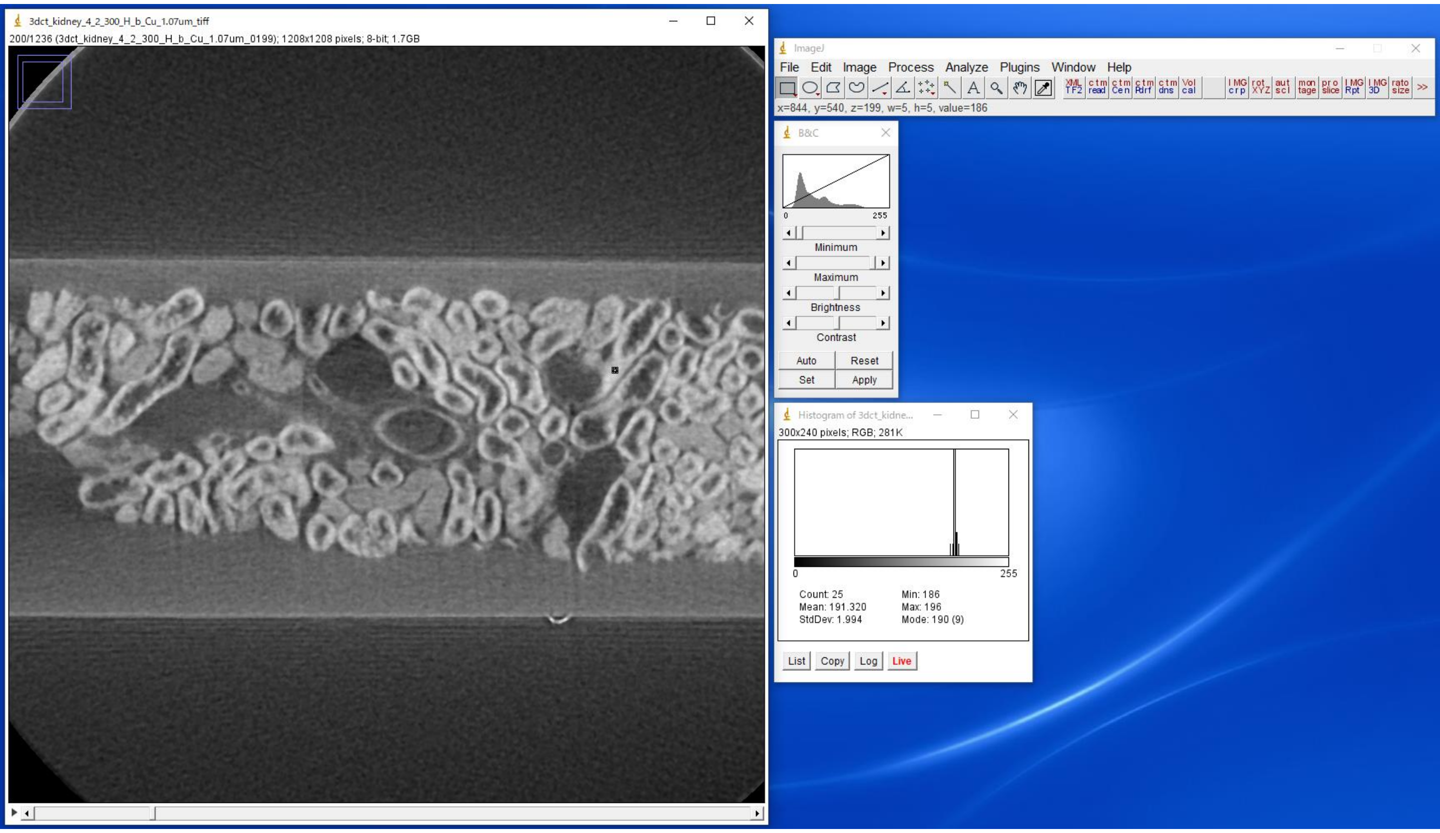

Supplementary Fig. 1. CNR measurement using the program *ImageJ* for CCD data.

Distal tubule measurement for 2_300_H_b nephron 5: air (top) and material (bottom) regions.

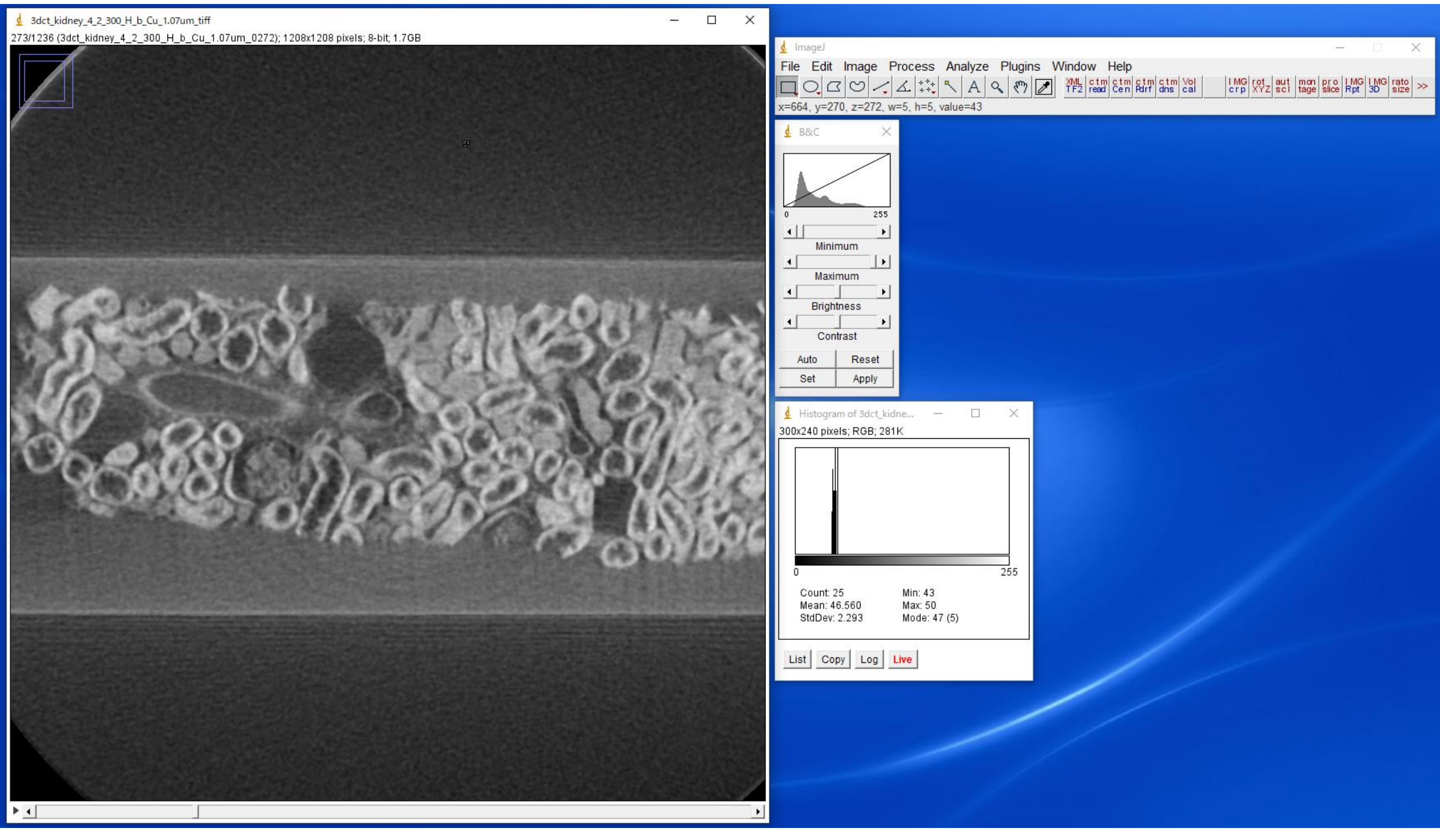

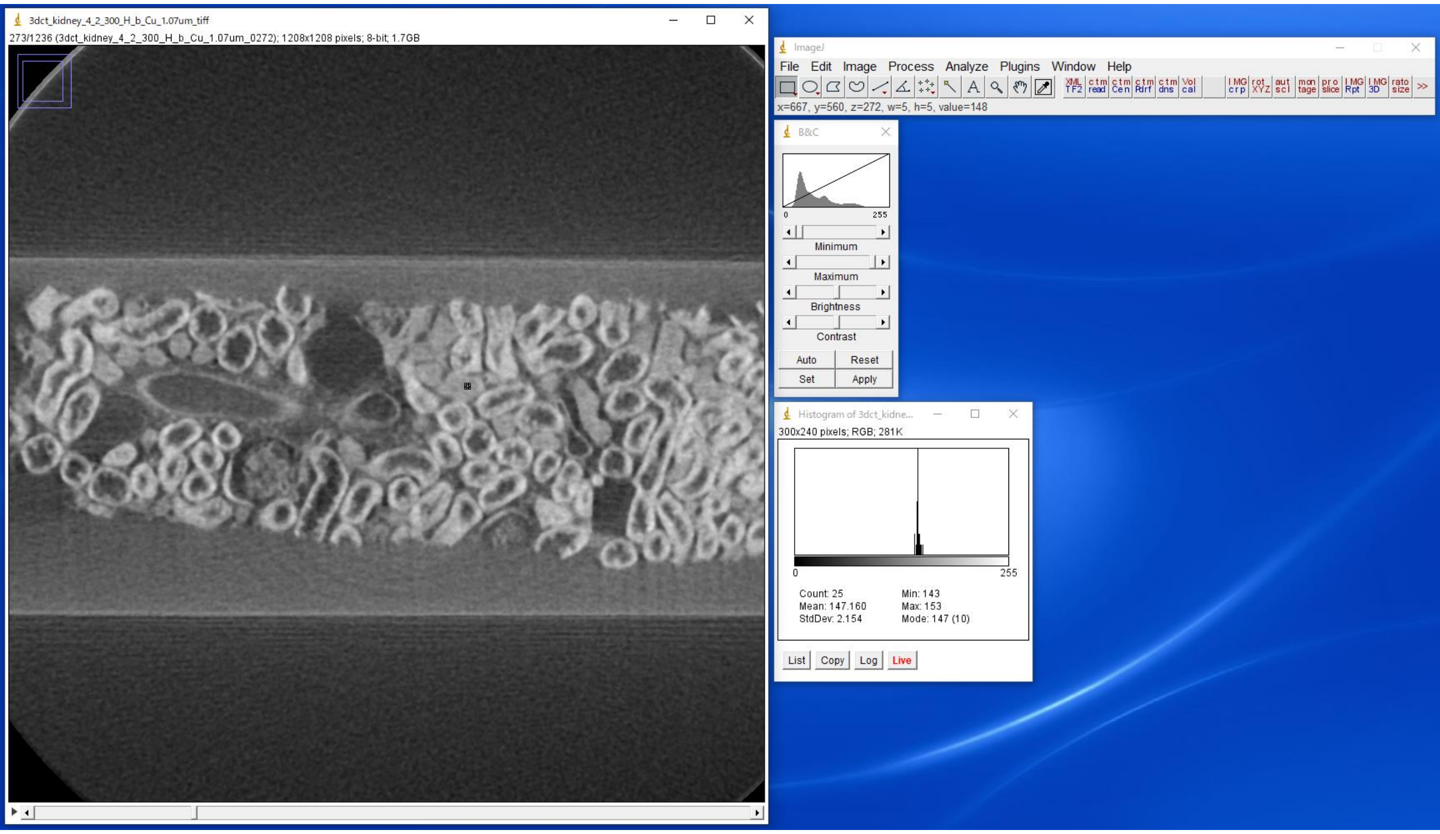

Supplementary Fig. 1. CNR measurement using the program *ImageJ* for CCD data.

Proximal tubule measurement for 2_500_H_b nephron 1: air (top) and material (bottom) regions.

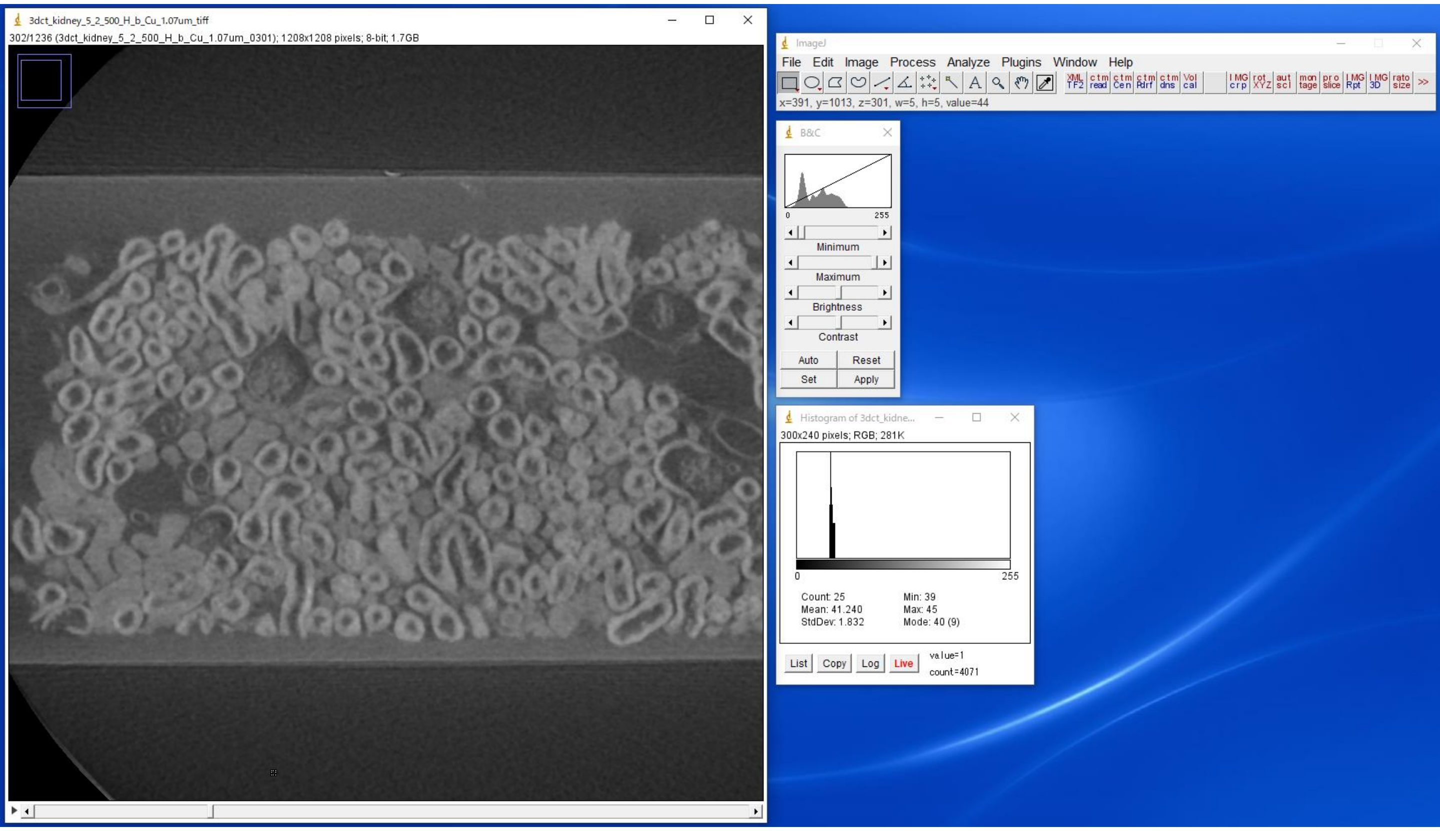

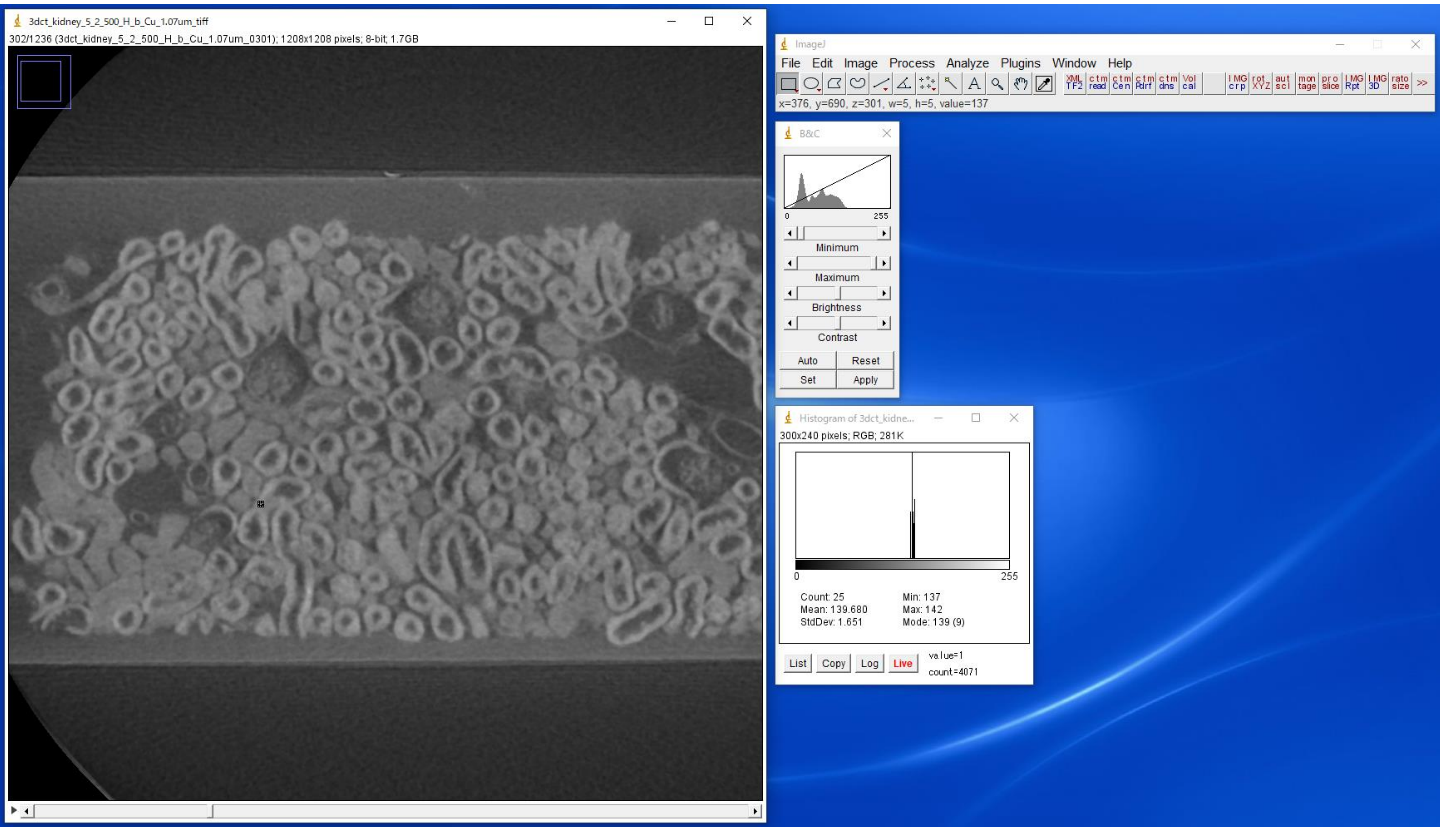

Supplementary Fig. 1. CNR measurement using the program *ImageJ* for CCD data.

Distal tubule measurement for 2_500_H_b nephron 1: air (top) and material (bottom) regions.

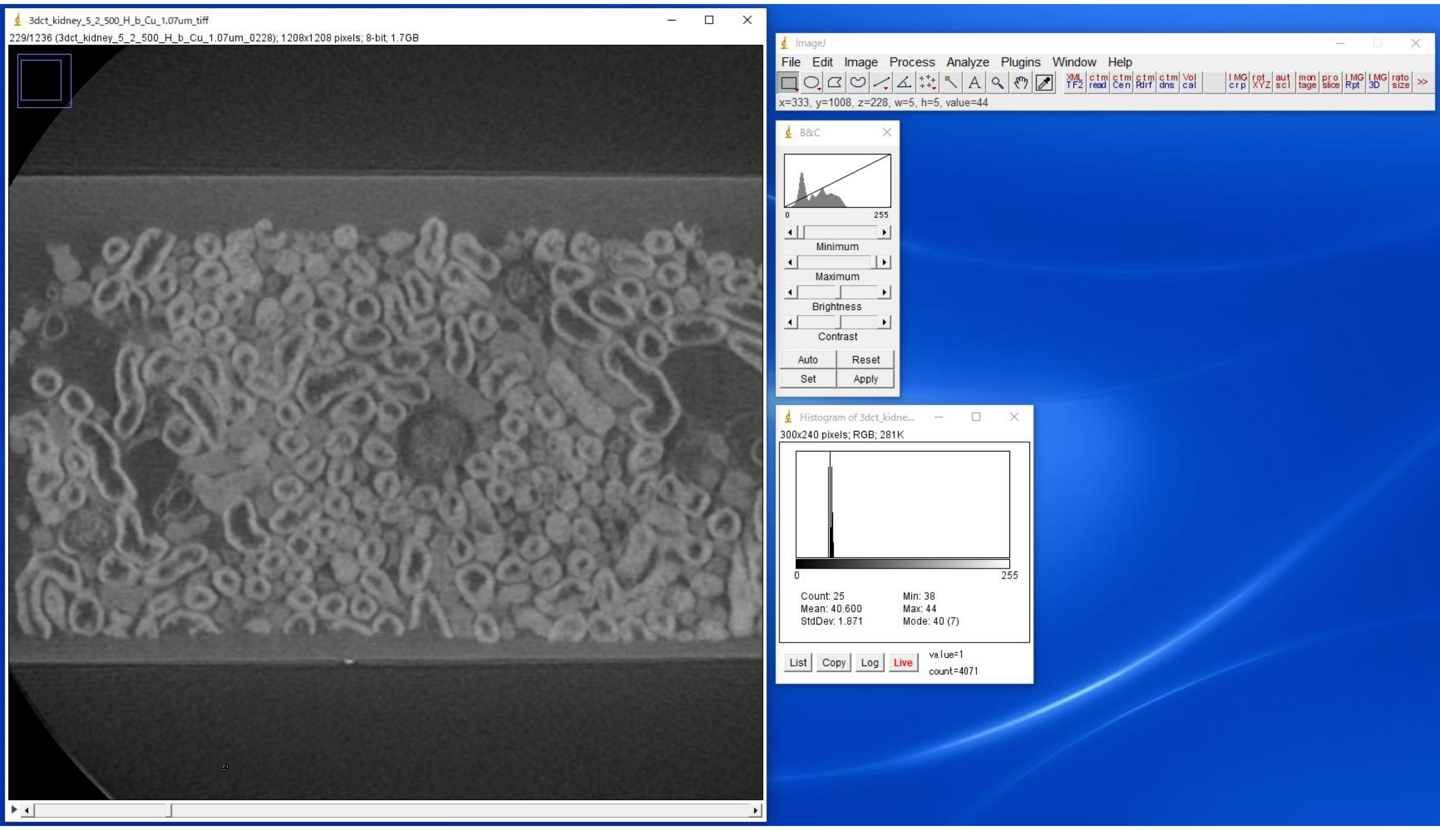

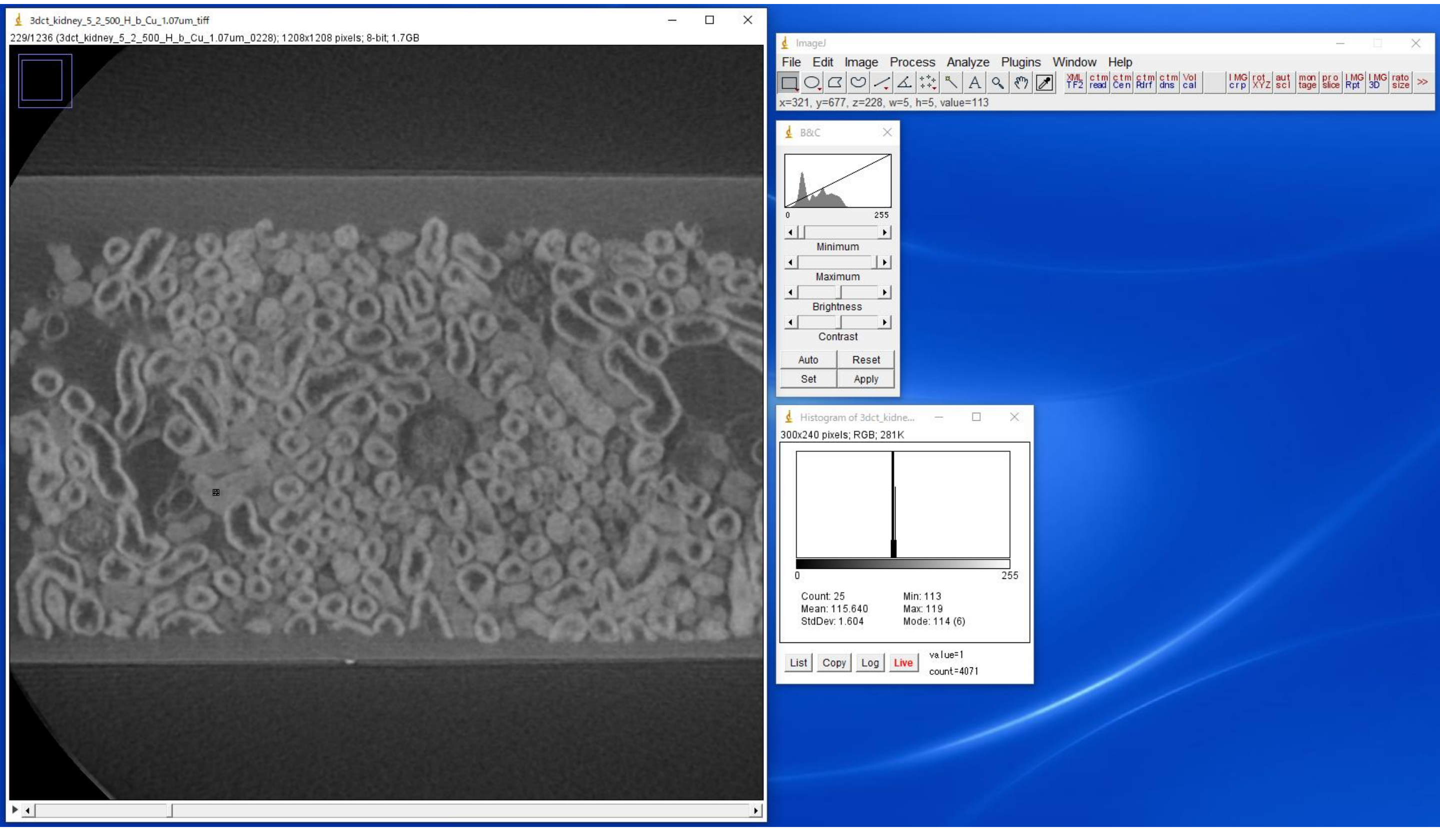

Supplementary Fig. 1. CNR measurement using the program *ImageJ* for CCD data.

Proximal tubule measurement for 2_500_H_b nephron 2: air (top) and material (bottom) regions.

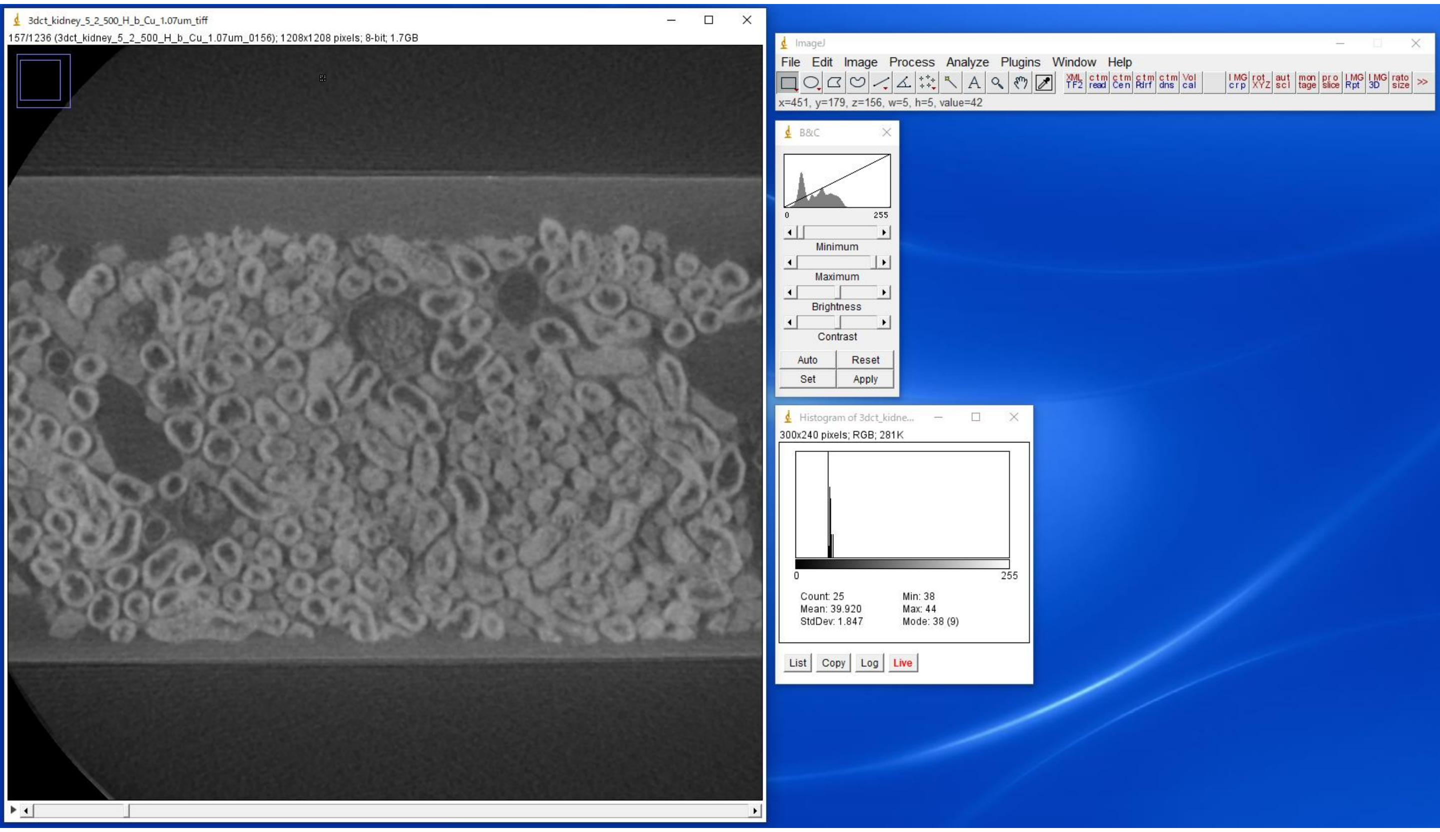

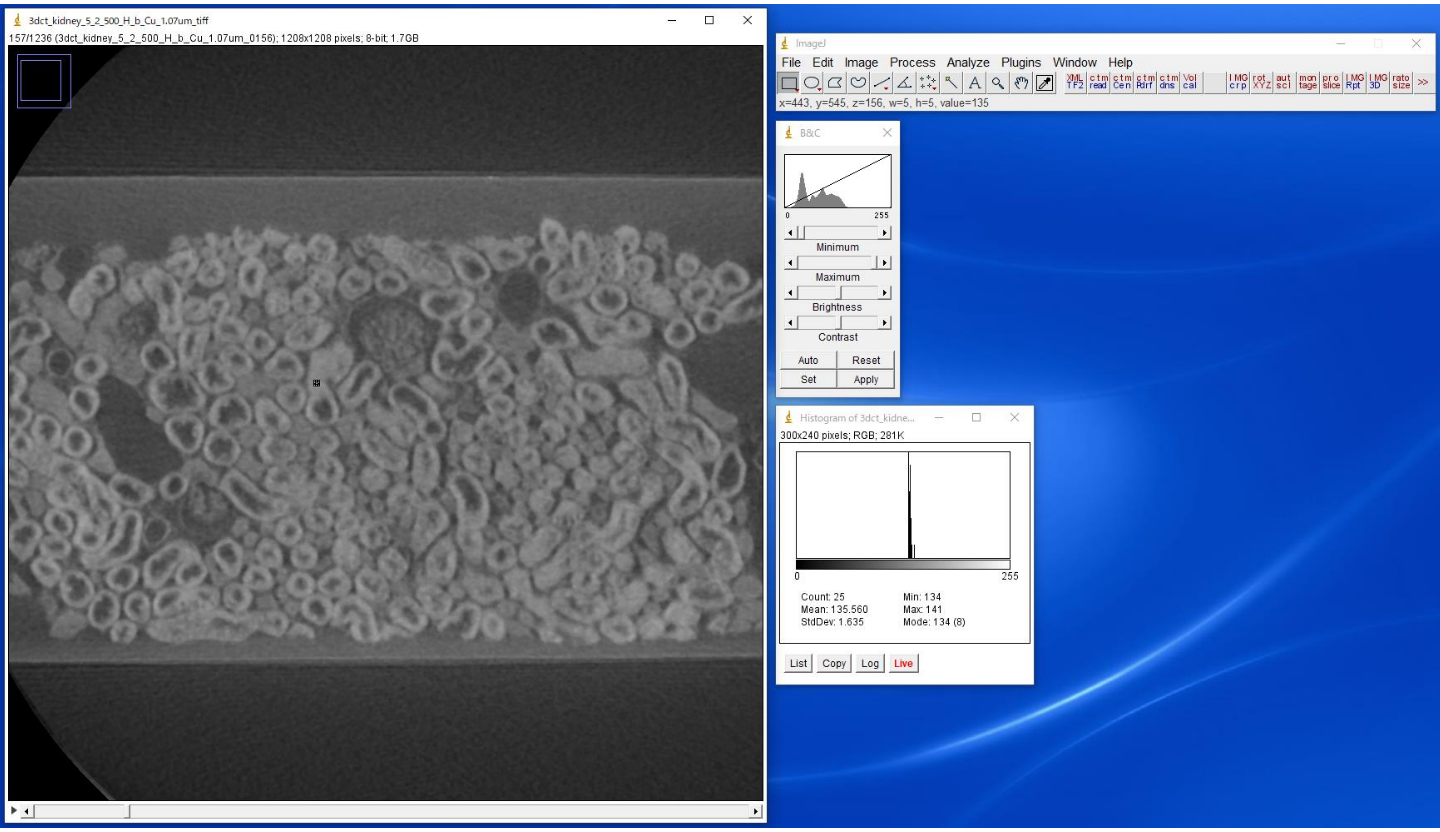

Supplementary Fig. 1. CNR measurement using the program *ImageJ* for CCD data.

Distal tubule measurement for 2_500_H_b nephron 2: air (top) and material (bottom) regions.

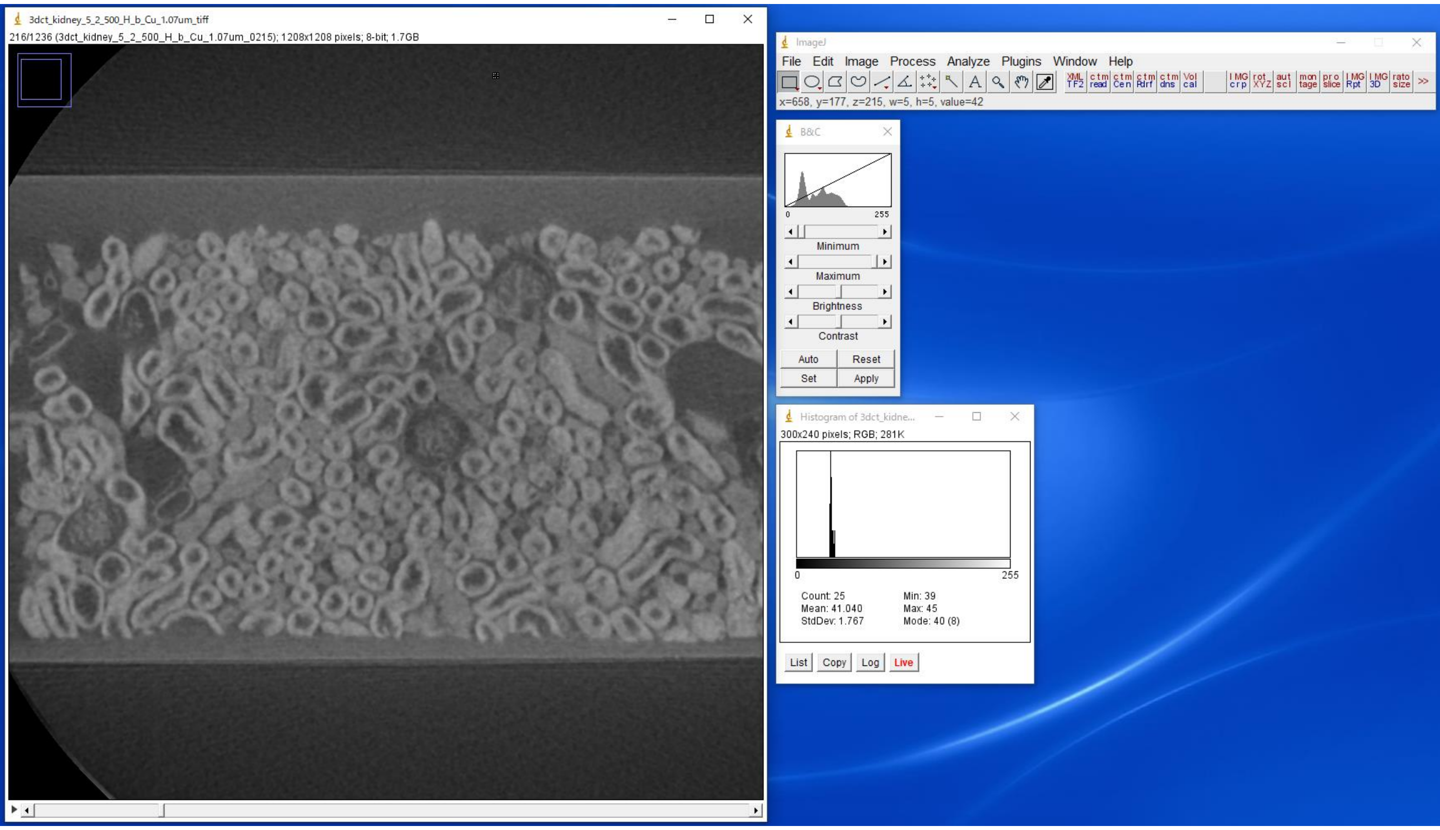

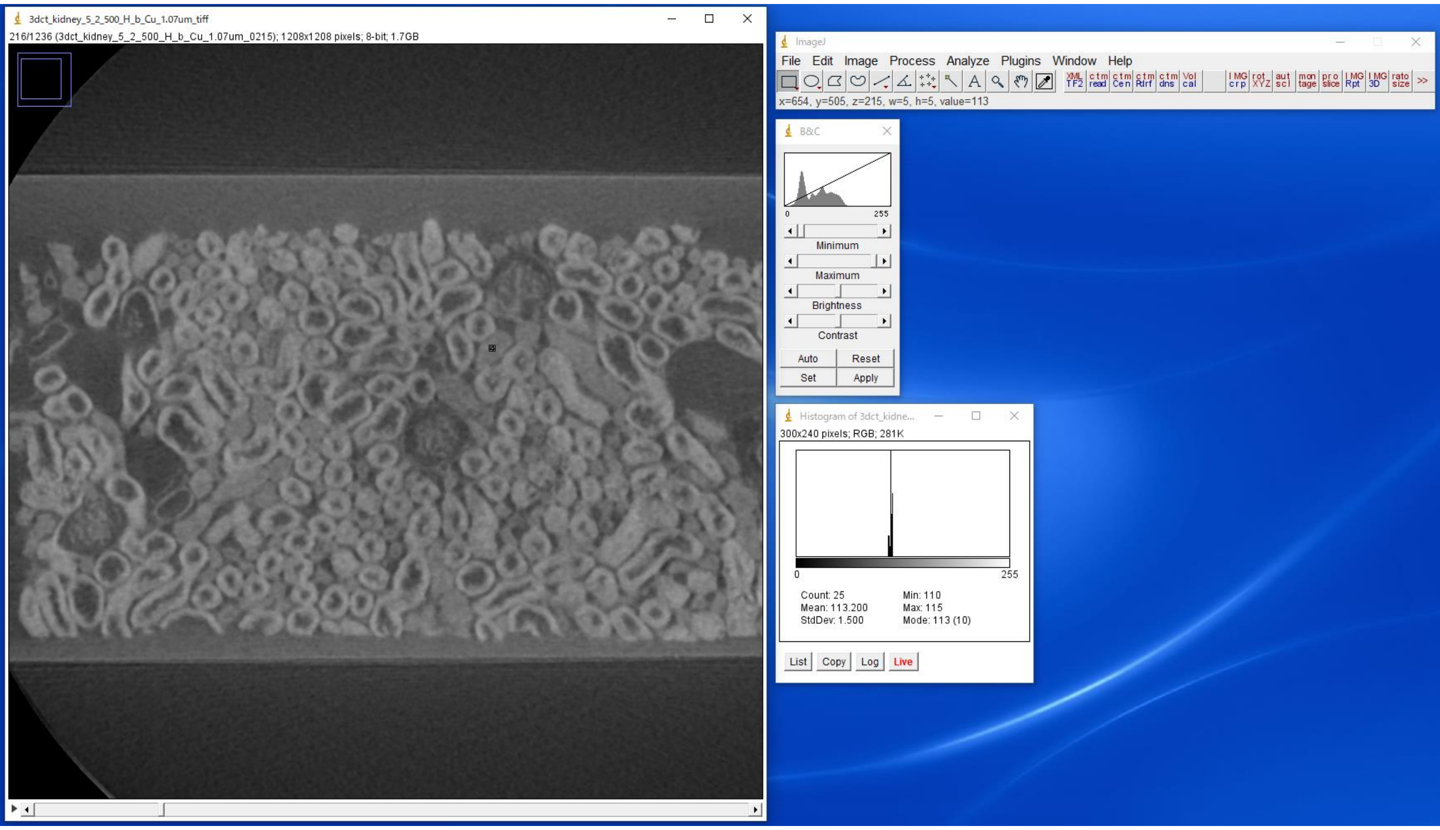

Supplementary Fig. 1. CNR measurement using the program *ImageJ* for CCD data.

Proximal tubule measurement for 2_500_H_b nephron 3: air (top) and material (bottom) regions.

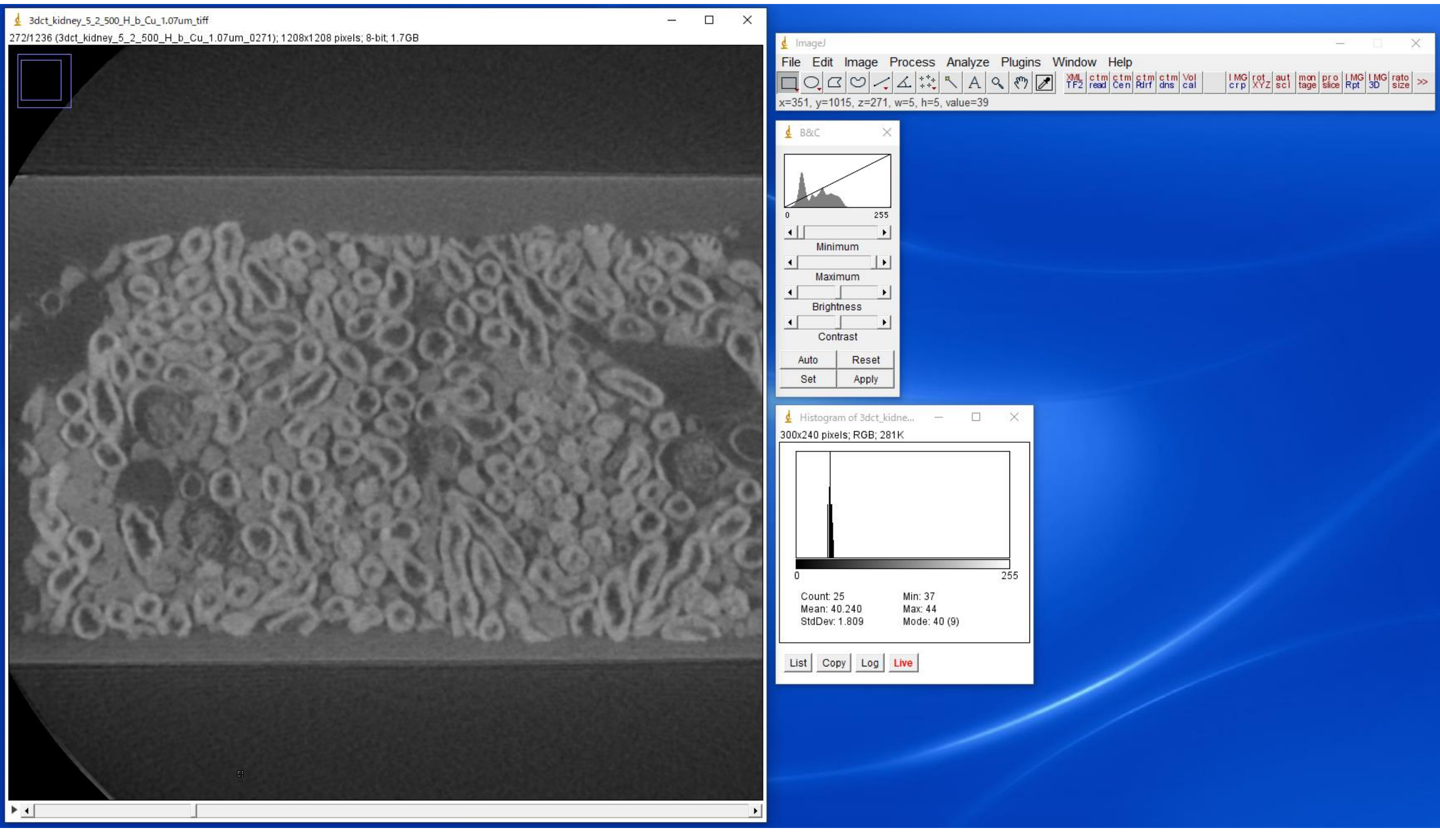

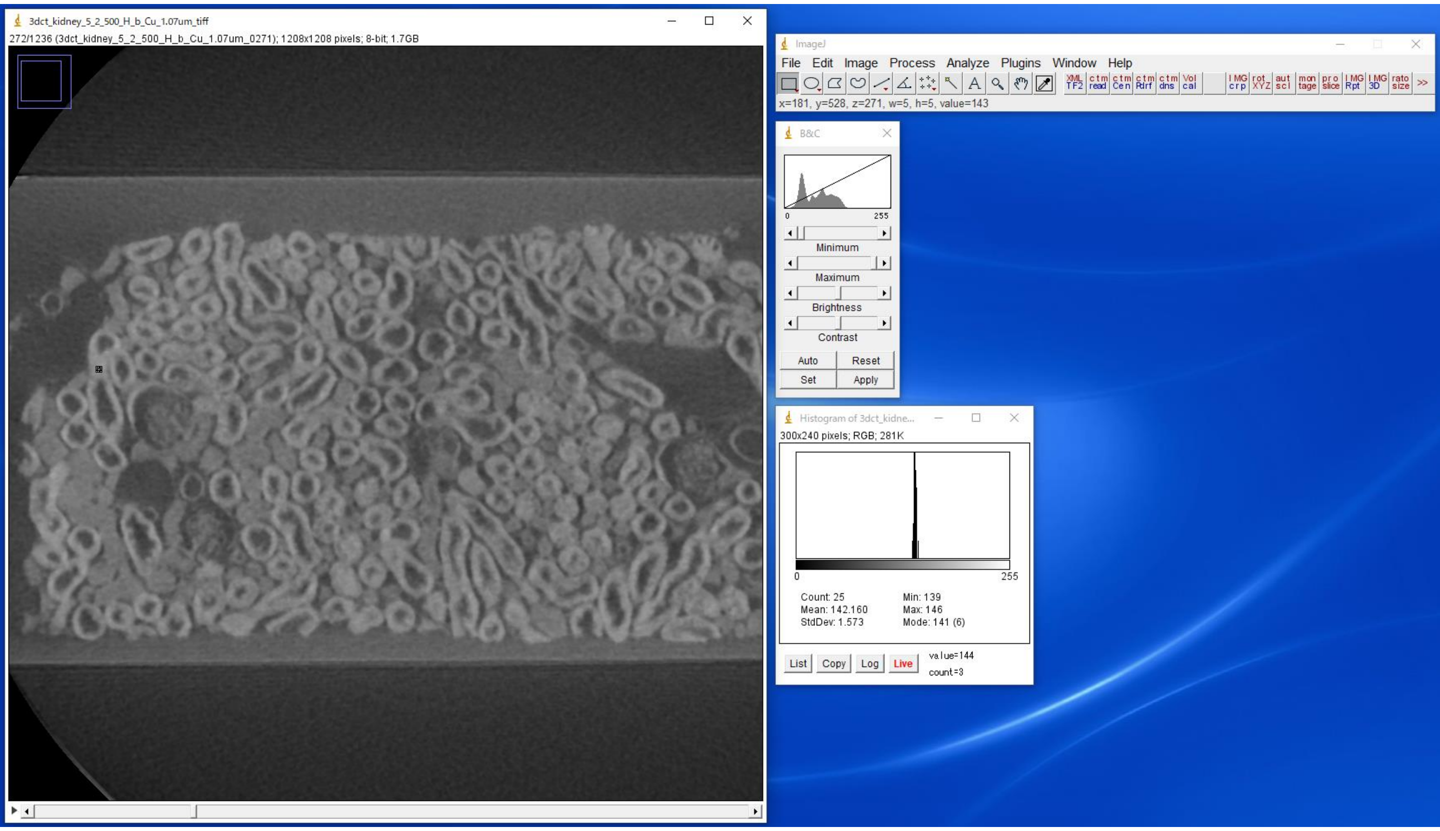

Supplementary Fig. 1. CNR measurement using the program *ImageJ* for CCD data.

Distal tubule measurement for 2_500_H_b nephron 3: air (top) and material (bottom) regions.

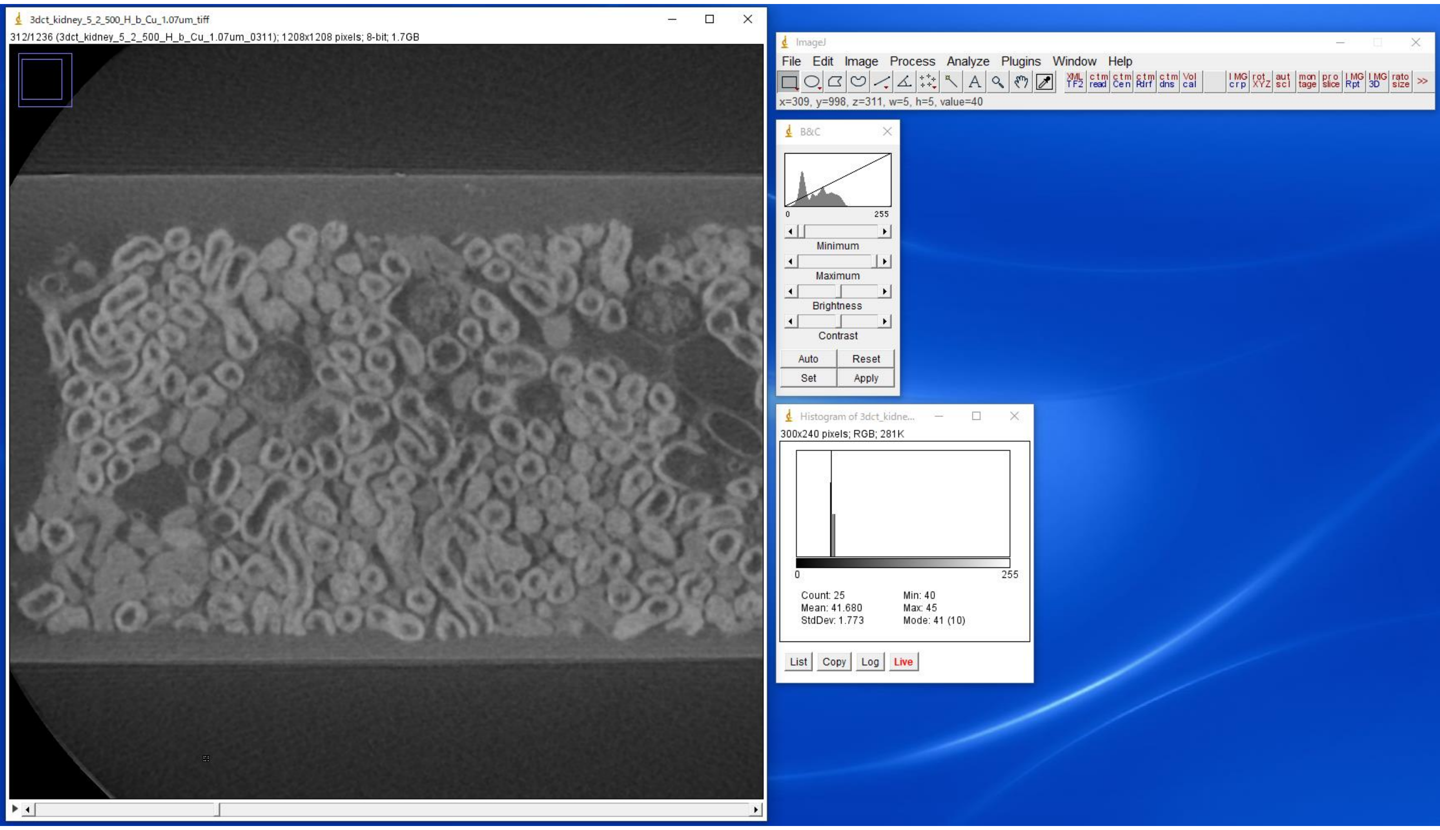

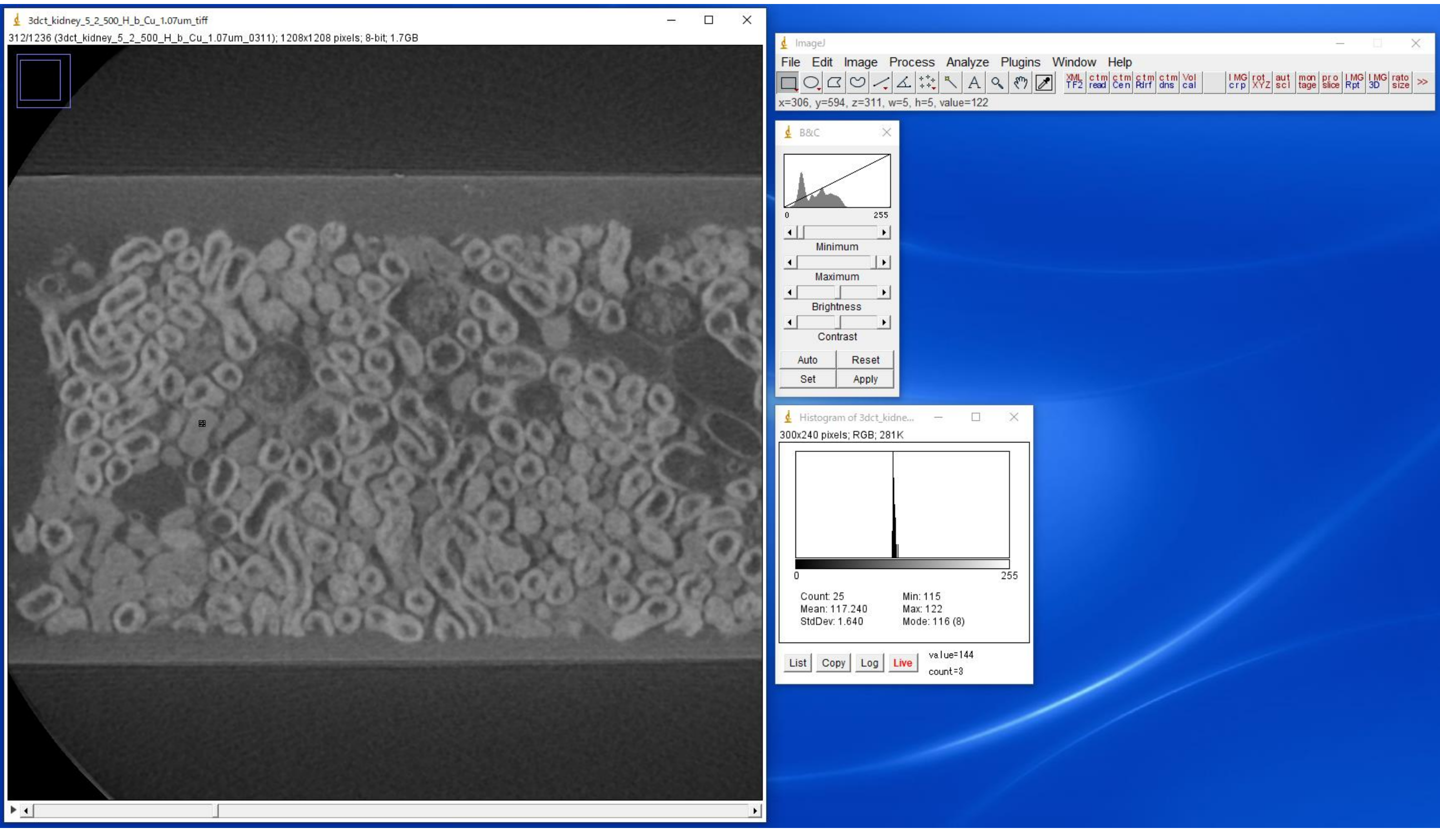

Supplementary Fig. 1. CNR measurement using the program *ImageJ* for CCD data.

Proximal tubule measurement for 2_500_H_b nephron 4: air (top) and material (bottom) regions.

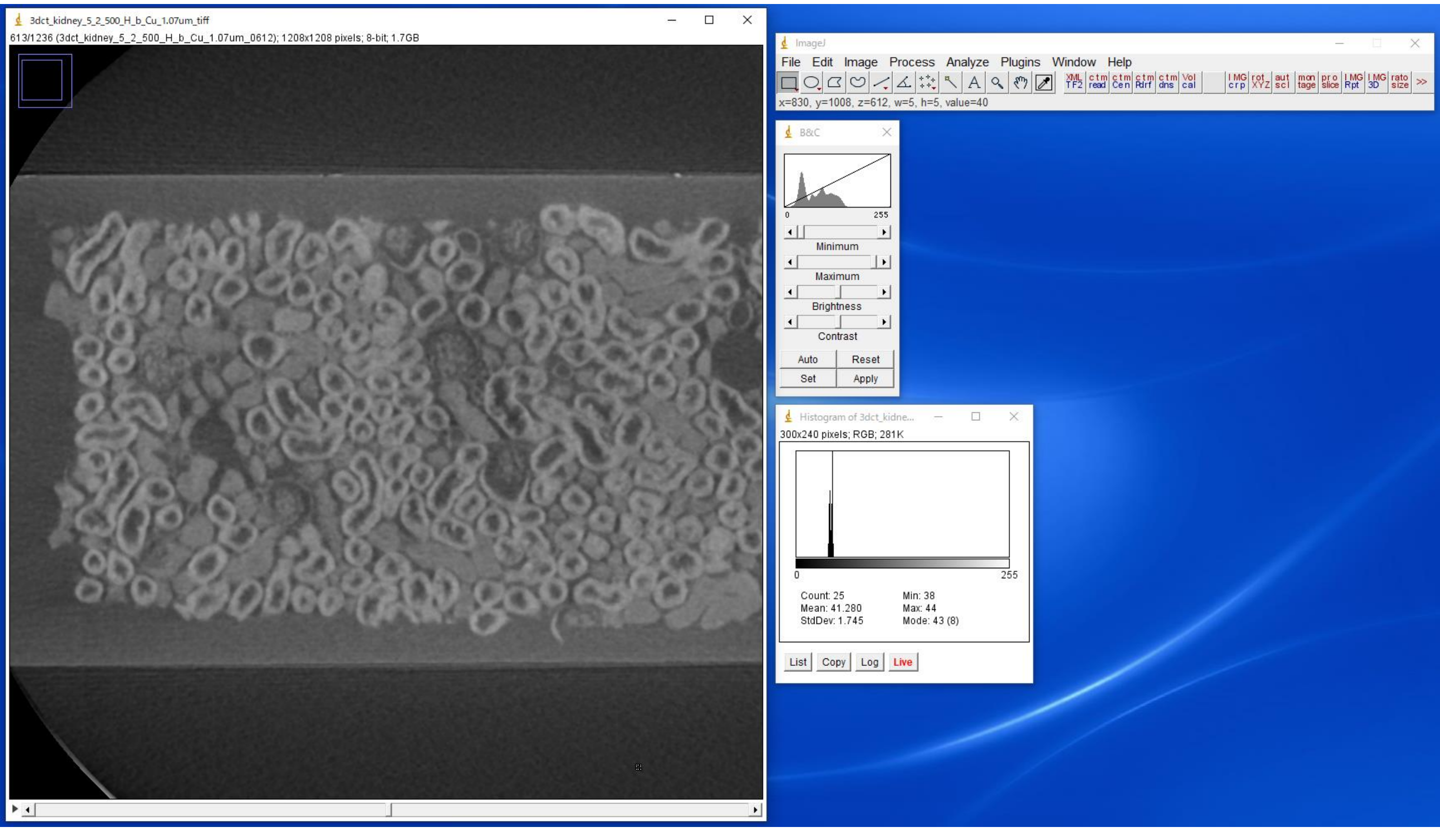

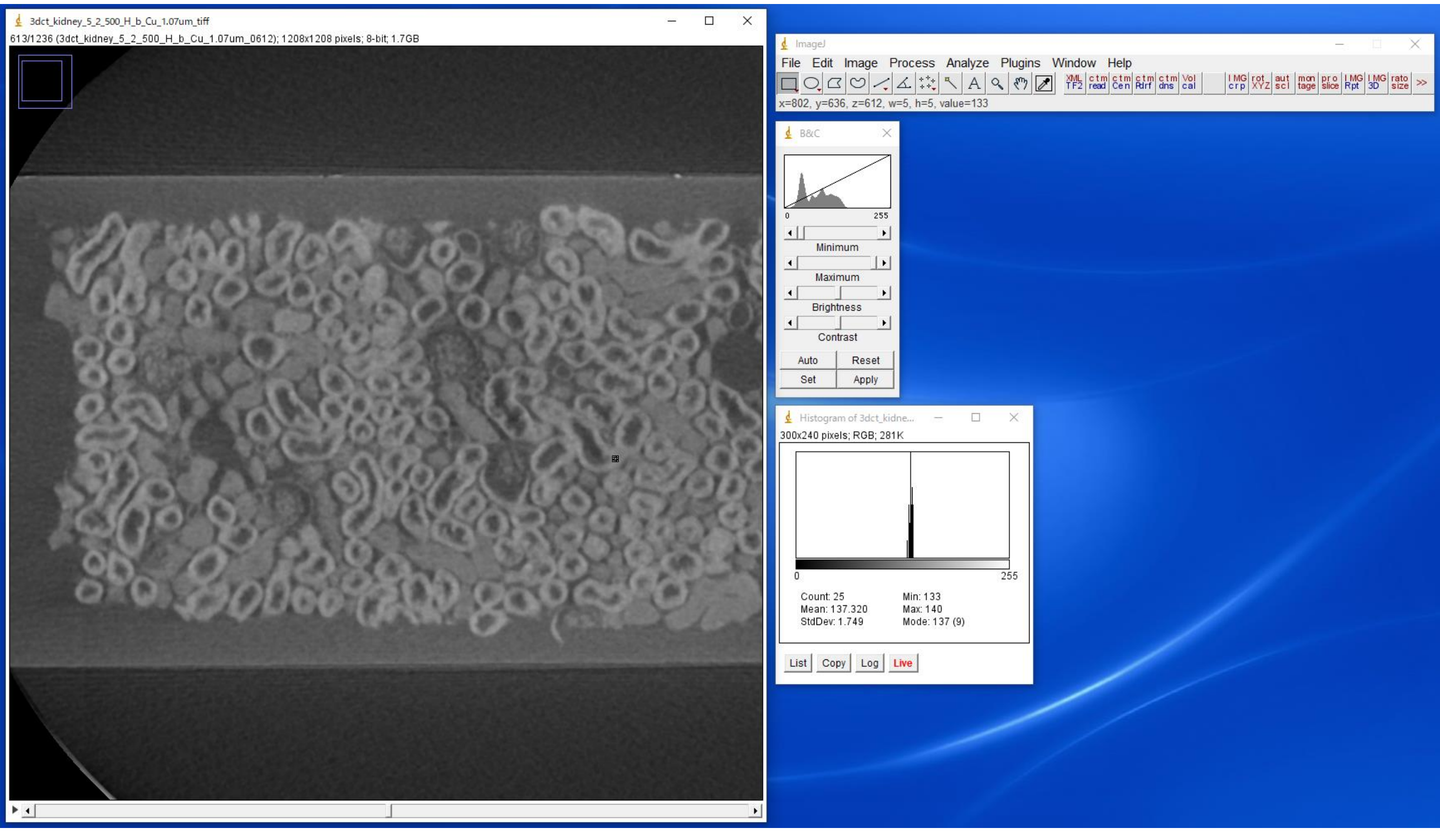

Supplementary Fig. 1. CNR measurement using the program *ImageJ* for CCD data.

Distal tubule measurement for 2_500_H_b nephron 4: air (top) and material (bottom) regions.

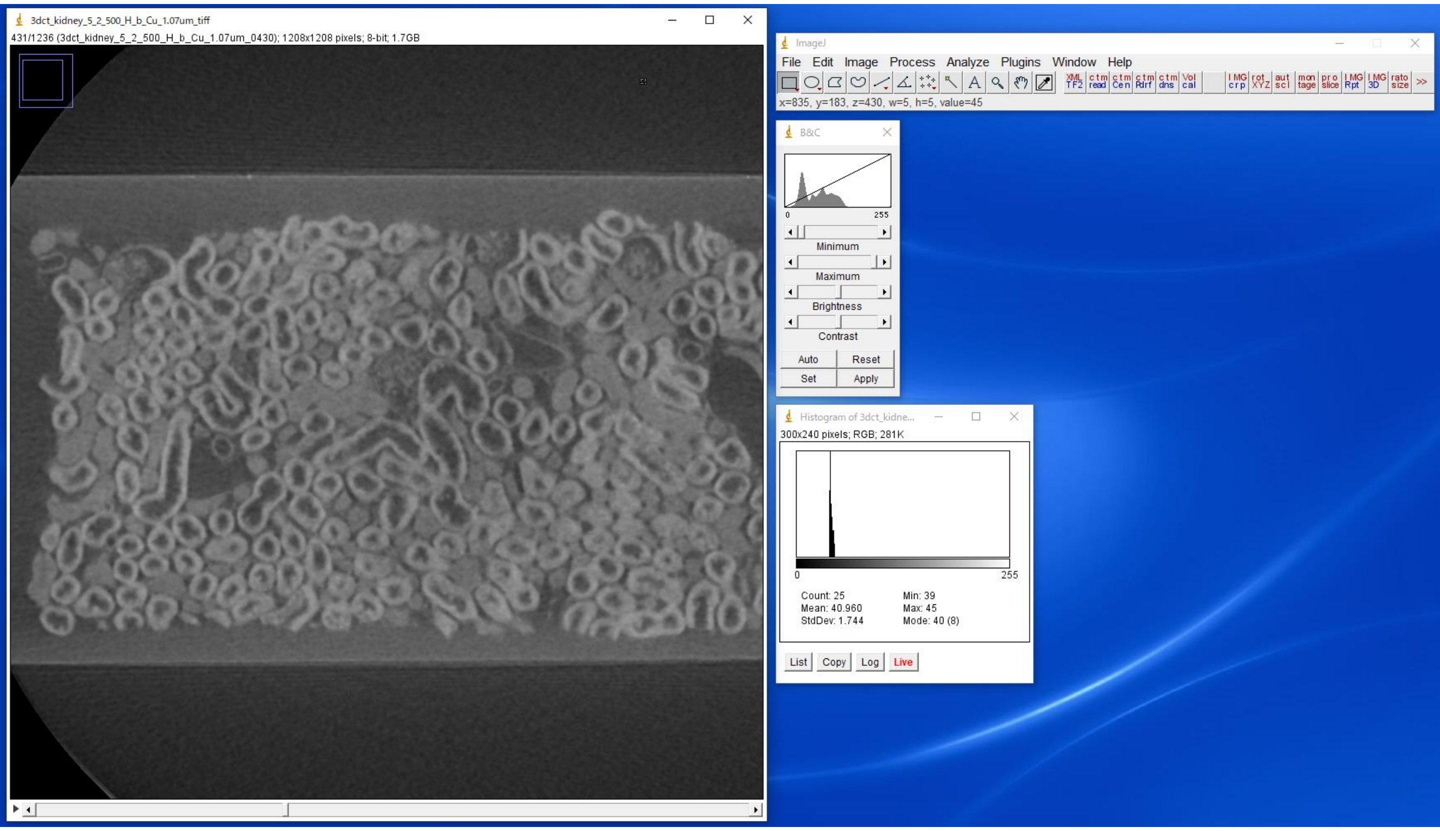

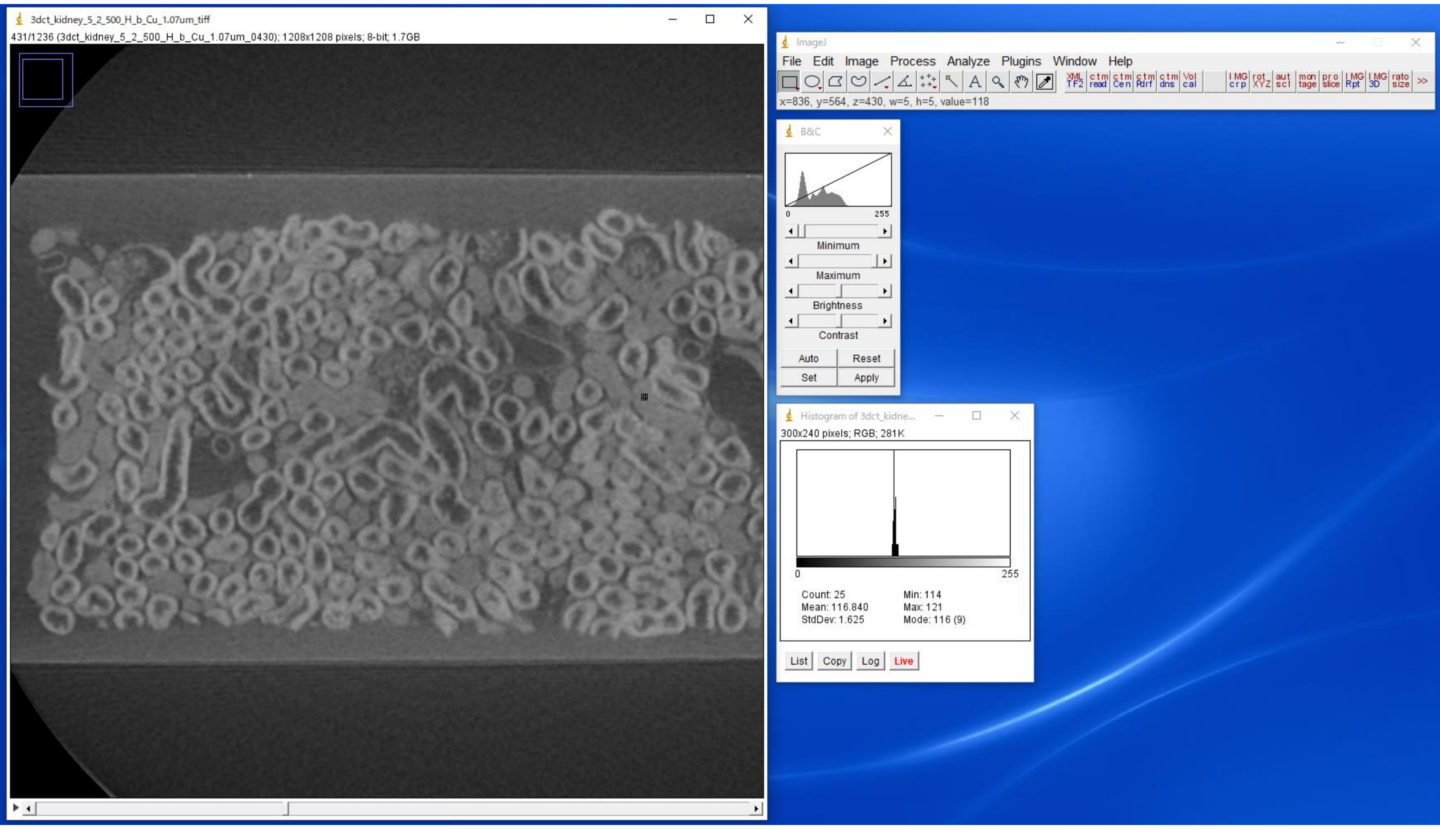

Supplementary Fig. 1. CNR measurement using the program *ImageJ* for CCD data.

Proximal tubule measurement for 2_500_H_b nephron 5: air (top) and material (bottom) regions.

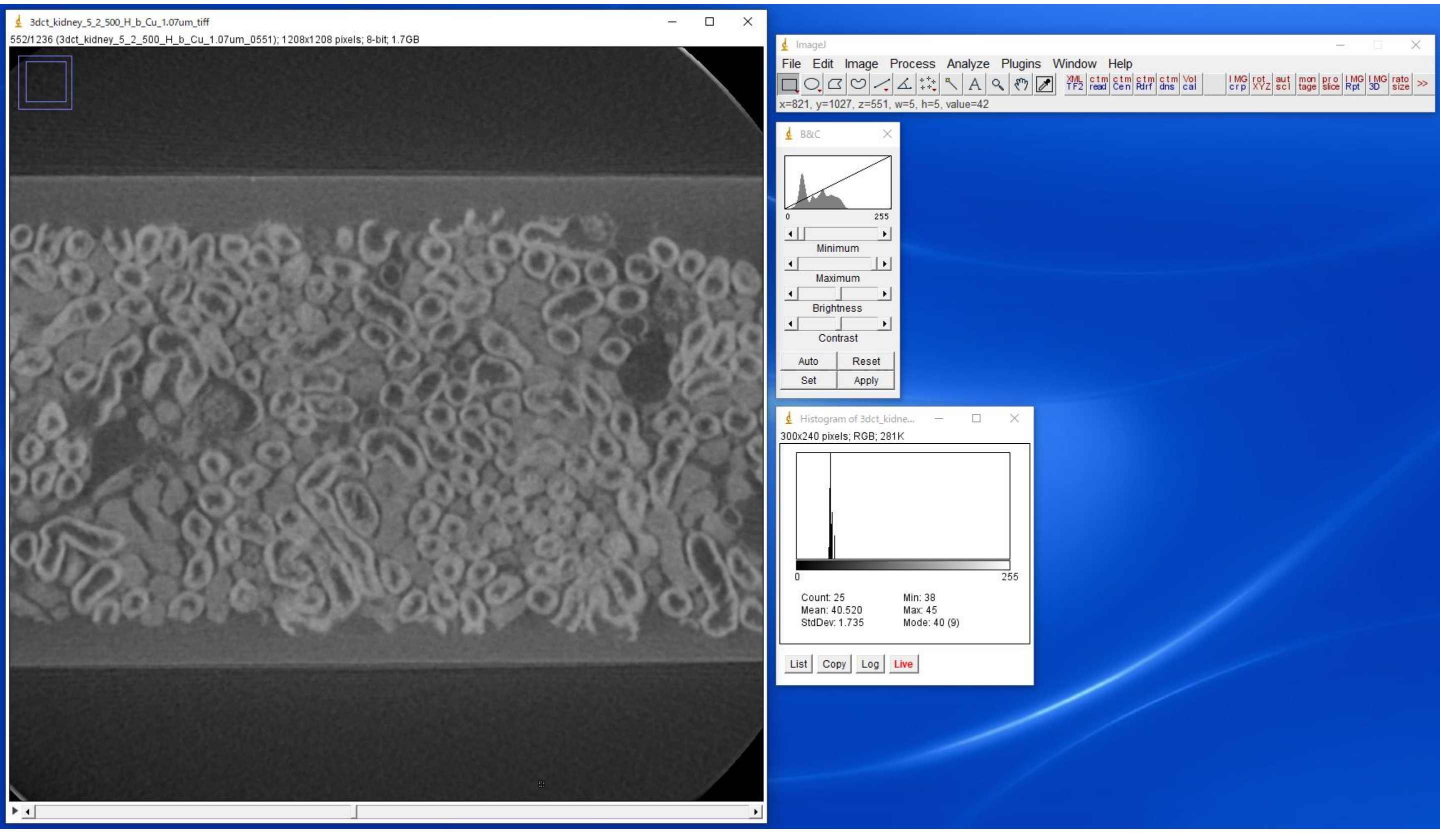

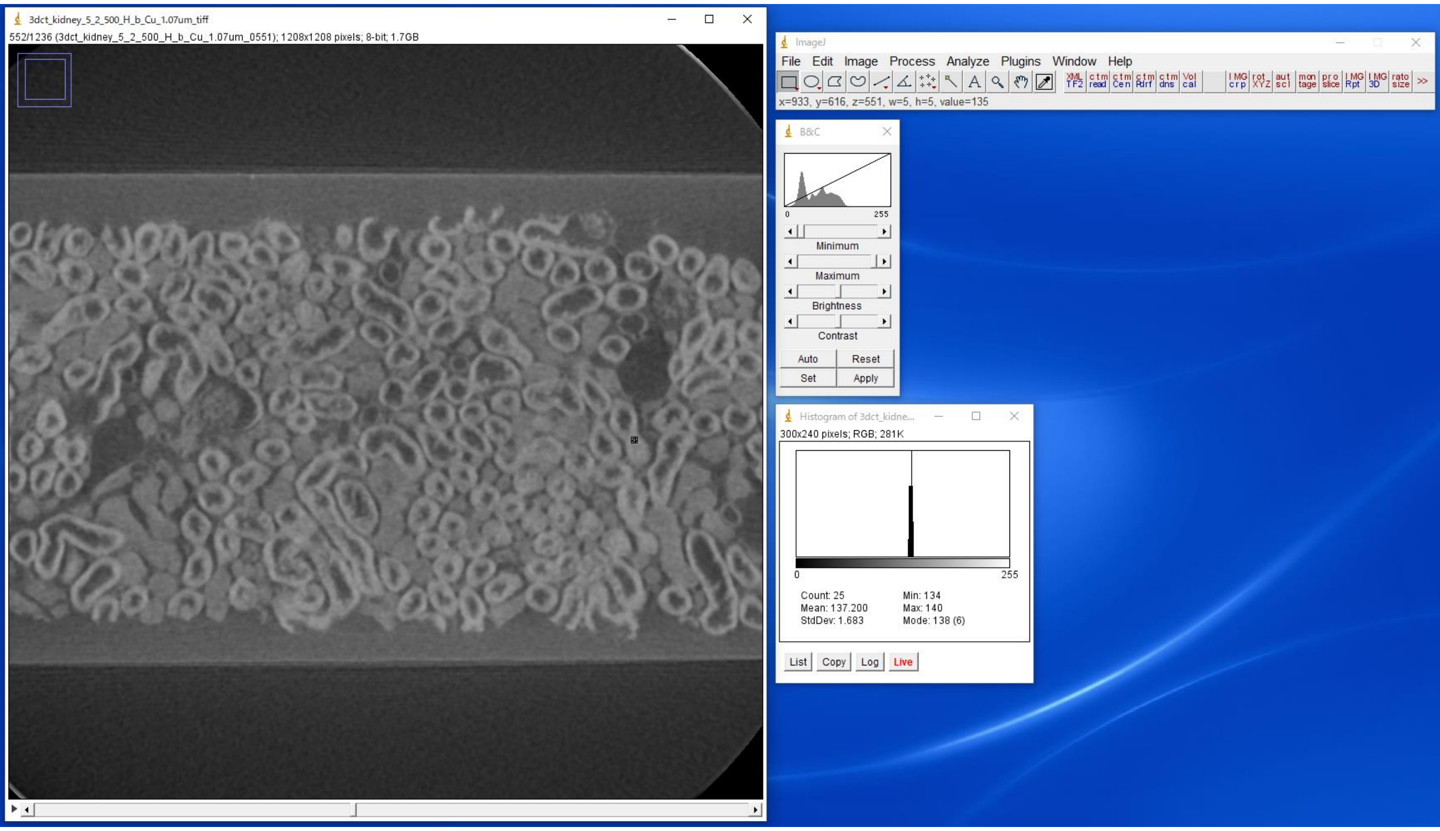

Supplementary Fig. 1. CNR measurement using the program *ImageJ* for CCD data.

Distal tubule measurement for 2_500_H_b nephron 5: air (top) and material (bottom) regions.

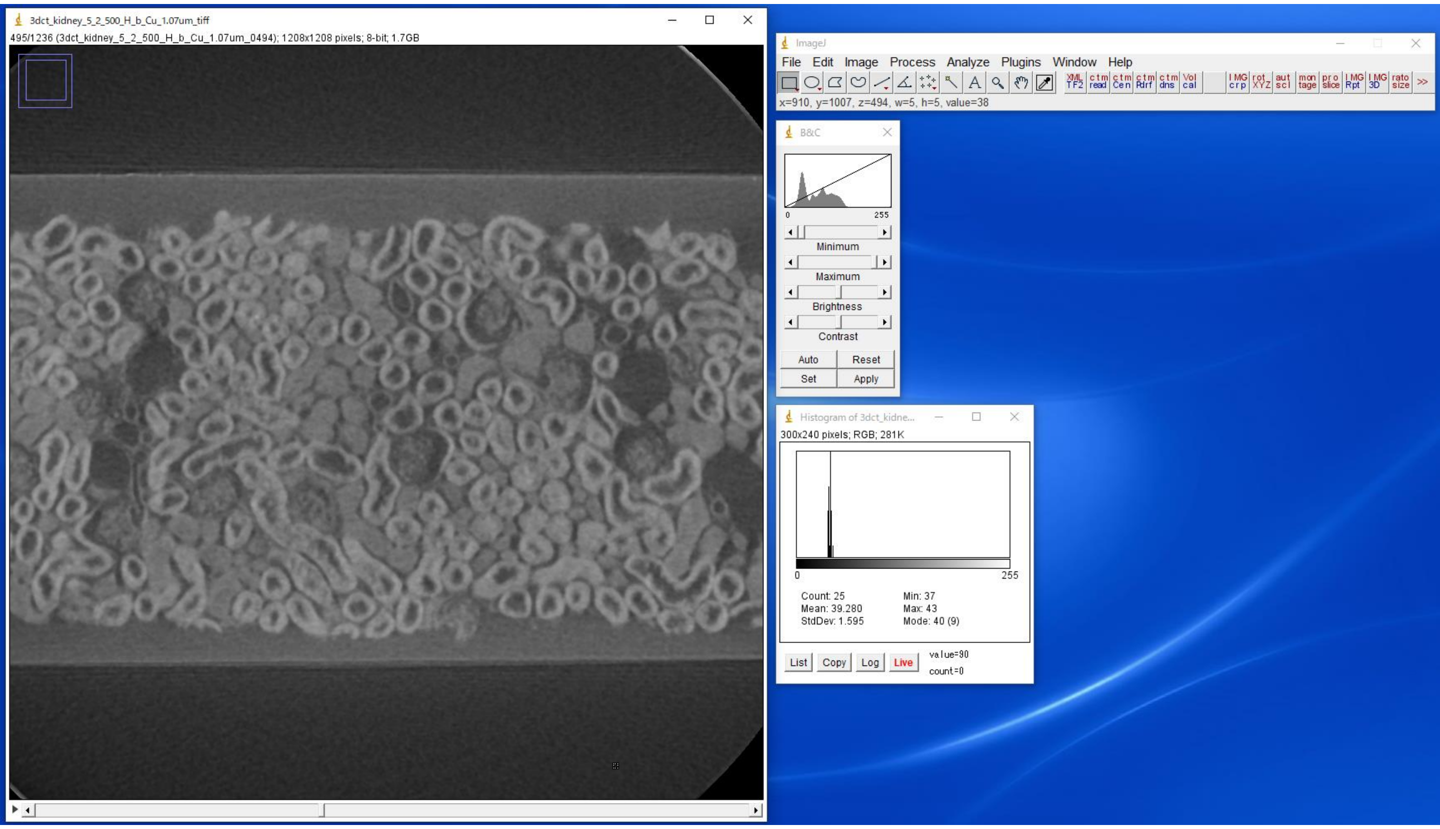

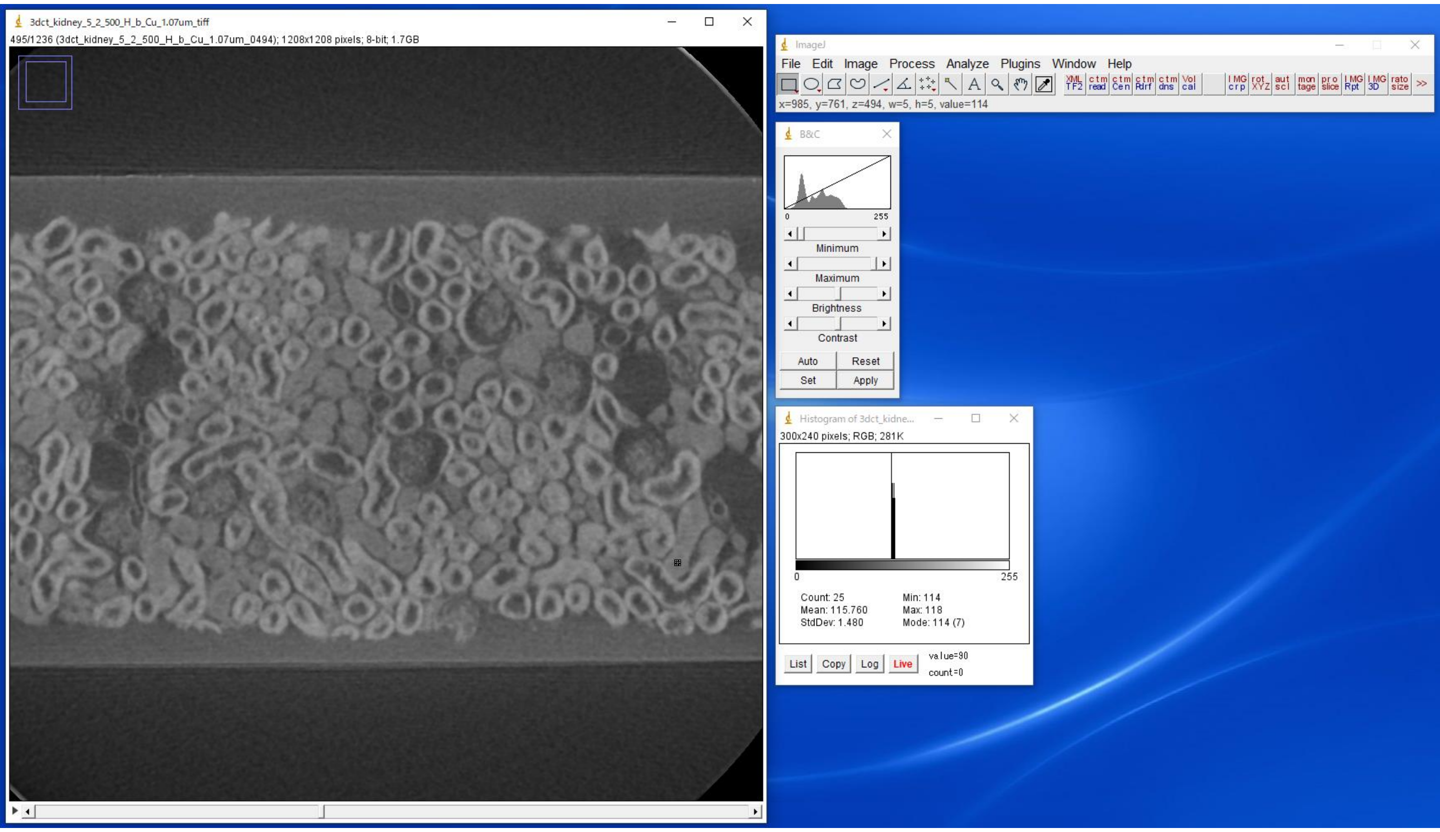

Supplement: dfac033_Supp [file dfac033_supp.zip › Supp_Fig_1.pdf]
